# First case report of splenomegaly with splenic infarction due to aortic graft infection

**DOI:** 10.1186/s12872-023-03259-y

**Published:** 2023-05-05

**Authors:** Yuntae Kim, Kazuhiro Ishikawa, Fujimi Kawai, Nobuyoshi Mori

**Affiliations:** 1 Department of Infectious Diseases, St. Luke’s International Hospital, 9-1, Akashi-Cho, Chuo-Ku, Tokyo Japan; 2 Library, Center for Academic Resources, St. Luke’s International University, Chuo-Ku, Tokyo Japan

**Keywords:** Aortic graft infection, Vascular graft infection, Bacteremia, Splenomegaly, Splenic infarction

## Abstract

**Background:**

Diagnosis of aortic graft infections (AGI) is challenging. Here, we report a case of AGI with splenomegaly and splenic infarction.

**Case presentation:**

A 46-year-old man who underwent total arch replacement for Stanford type A acute aortic dissection one year prior presented to our department with fever, night sweat, and a 20-kg weight loss over several months. Contrast-enhanced computed tomography (CT) revealed splenic infarction with splenomegaly, fluid collection, and thrombus around the stent graft. Positron emission tomography-CT (PET-CT) revealed abnormal ^18^F-fluorodeoxyglucose uptake in the stent graft and spleen. Transesophageal echocardiography revealed no vegetations. The patient was diagnosed with an AGI and underwent graft replacement. Blood and tissue cultures in the stent graft yielded *Enterococcus faecalis*. After the surgery, the patient was successfully treated with antibiotics.

**Conclusions:**

Splenic infarction and splenomegaly are the clinical findings of endocarditis but are rare in graft infection. These findings could be helpful to diagnose graft infections, which is often challenging.

**Supplementary Information:**

The online version contains supplementary material available at 10.1186/s12872-023-03259-y.

## Background

Aortic graft infection (AGI) is a rare but potentially fatal condition. The rate of AGI following aortic grafting has generally been reported to be between 1 and 3% [1]. Diagnosis of AGI is challenging. Computed tomography (CT) often reveals fluid collection more than three months after insertion and peri-graft gas more than seven weeks after insertion [2]. On the other hand, splenomegaly is a known finding in infective endocarditis [3], but there have been no case reports of splenomegaly with intravascular infection. In addition, emboli to the brain, lung, or spleen occur in 30% of patients as the presenting feature [4]. We report the first case of splenomegaly and splenic infarction in a patient with an AGI.

## Case presentation

The patient was a 46-year-old man who had undergone vascular grafting of the ascending aorta and aortic arch for Stanford type A aortic dissection one year before coming to our department. He had experienced occasional fevers and night sweats for 6 months prior to his presentation to our department (4 months after surgery). He later presented weight loss of 10 kg and a fever of 39 °C, and visited the emergency room the day before coming to our department. At the time of his emergency visit, his CRP was only slightly elevated, with no obvious focus of fever, and his general condition was relatively good; therefore, blood cultures were taken, and follow-up was done as an outpatient. However, from blood cultures (4/4), gram-positive cocci were detected, and the patient was referred to our department for consultation and hospitalized the next day.

**Fig. 1:**
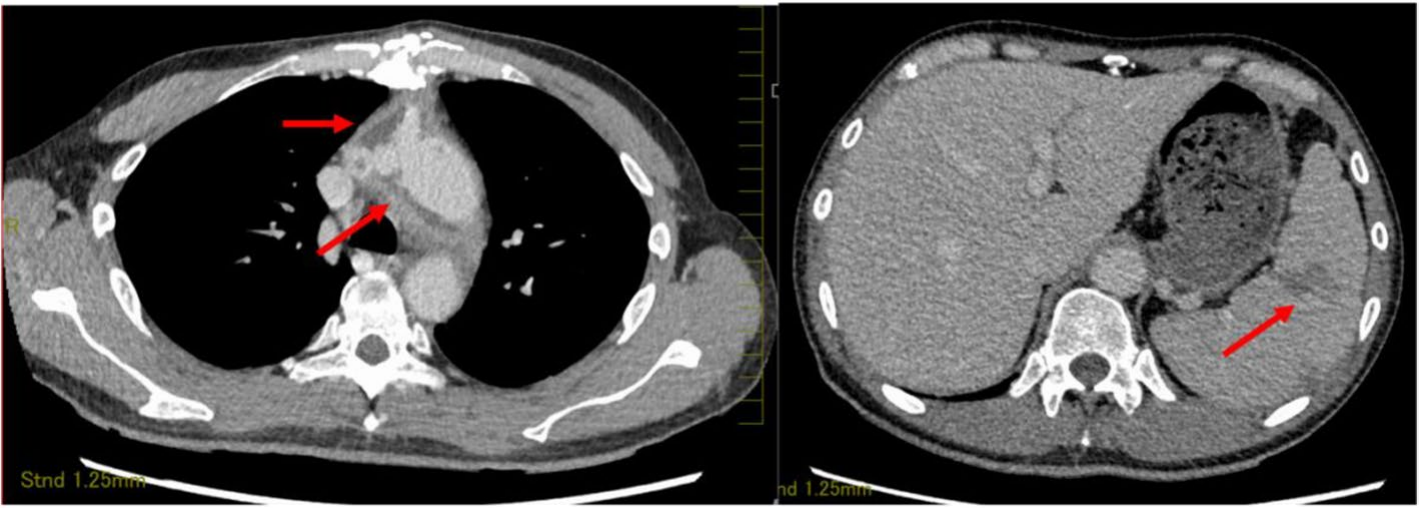
Contrast-enhanced computed tomography of aortic aorta (left), spleen, and liver (right). Contrast computed tomography (CT) revealed fluid correction around the graft vessel (left, arrow), hepatomegaly, splenomegaly (spleen index = 720), and multiple thrombi (right, arrow)

On admission, his vital signs were as follows: consciousness level clear, temperature 39.3 ℃, blood pressure 116/74 mmHg, pulse rate 96/min, respiratory rate 21/min, oxygen saturation 98% on room air. No abnormalities were found on head and neck examination. Heart and lung sounds were normal. Abdominal examination revealed no tenderness or hepatomegaly, but showed splenomegaly. There were no petechiae, Janeway lesions, Osler nodes, swollen or inflamed skin and joints, or lymphadenopathy. Laboratory data revealed 7, 700/μL (neutrophil 81.0%, lymphocyte 12.4%, monocyte 4.5%) of white blood cells; 7.9 mg/dL of C-reactive protein CRP; ferritin, 353 ng/mL; rheumatoid factor, 19 IU/mL; C3, 83 mg/dL; C4, 28 mg/dL; anti-nuclear antibody < 40 titers; PR3-ANCA, 2.7 IU/mL; MPO-ANCA, 1.4 IU/mL; and soluble Interleukin 2 receptor 1130 U/mL. T-SPOT.TB was negative. Urinary dip test revealed protein 3 + and blood 1 + . Contrast-enhanced computed tomography (CT) revealed fluid correction around the aortic graft, hepatomegaly (spleen index = 720), and splenomegaly with multiple thrombi (Fig. 1).

**Fig. 2:**
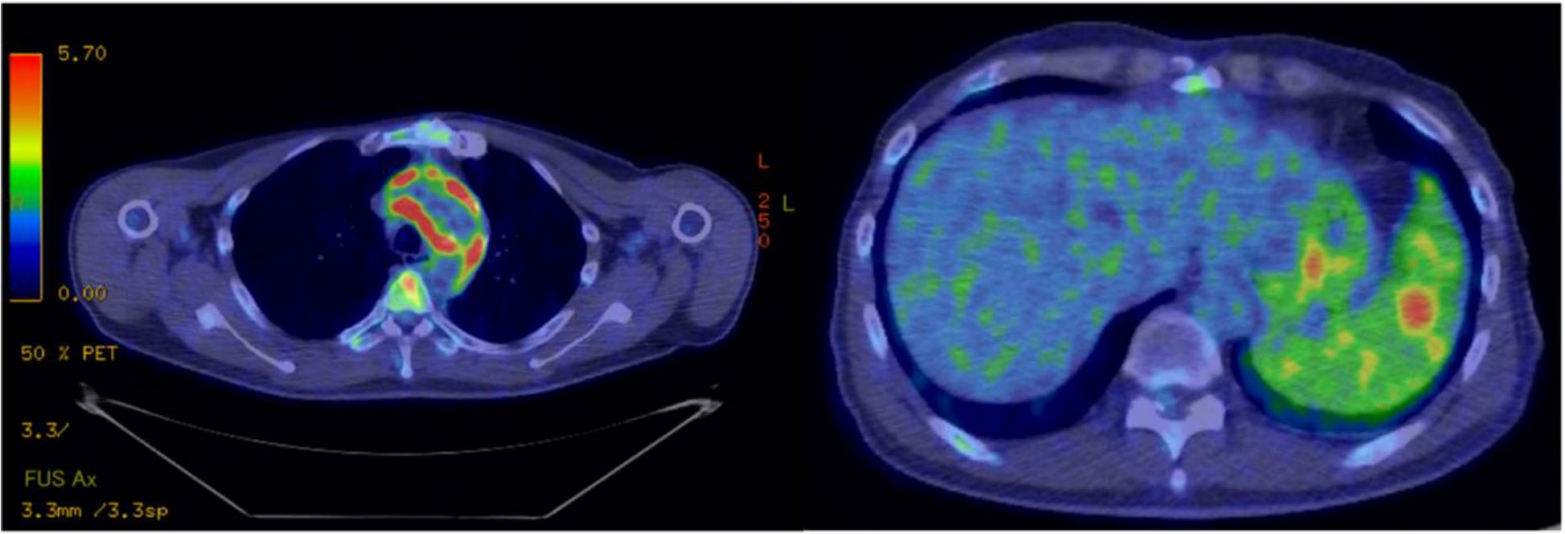
Positron-emission tomography-computed tomography. Hyperaccumulation (SUVmax: 11.8) in the fluid collection around the graft (left). Hyperaccumulation was observed throughout the spleen (SUVmax: 4.1), and localized hyperaccumulation was consistent with the area of splenic infarction (SUVmax:5.5) (right)

**Fig. 3:**
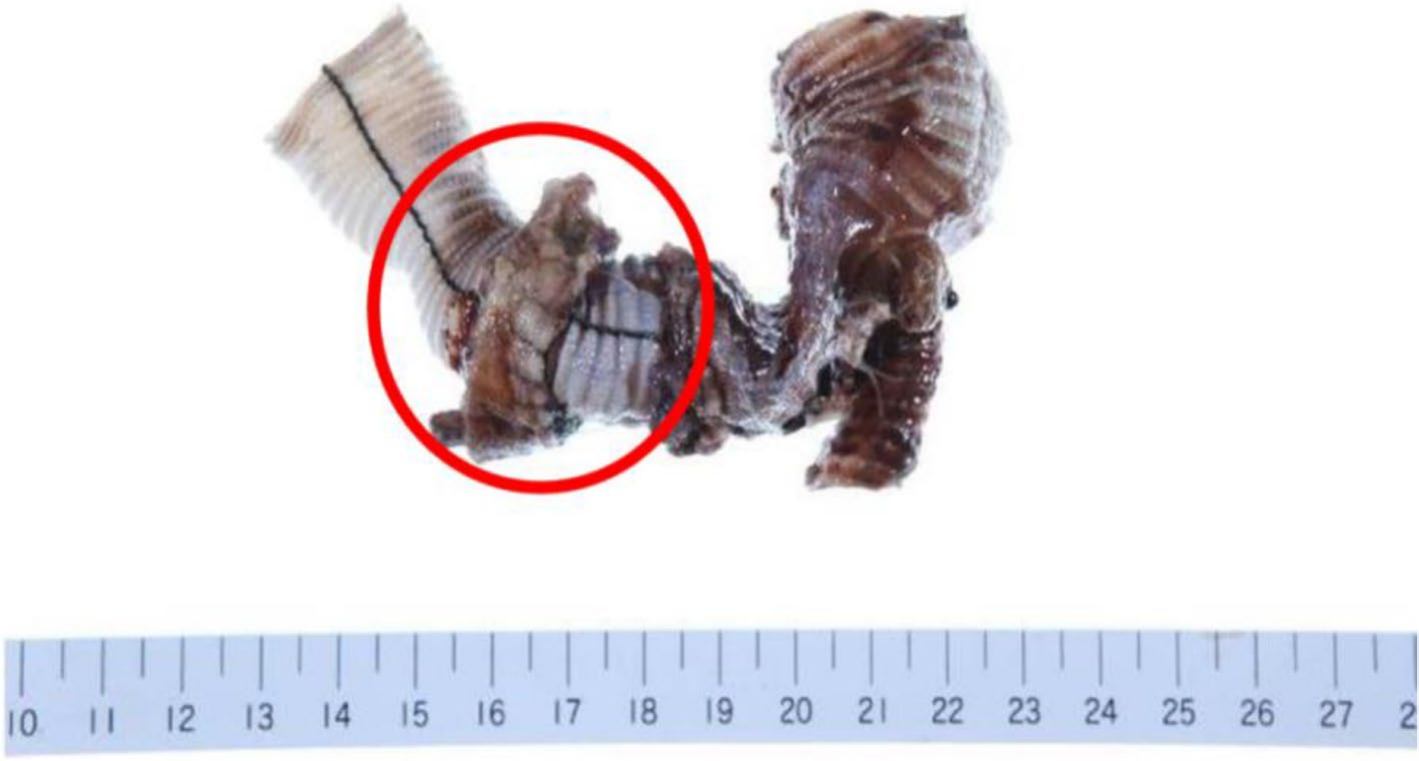
Clinical picture of the infected aortic graft removed in surgery. Pathological examination revealed inflammatory cell infiltration with neutrophils, foam cells, and foreign body giant cells with fibrosis, including fibroblasts, in the aortic graft. Debris and hemosiderin deposits were also observed in the hematoma. The tissue in the red circle was positive for *Enterococcus faecalis*

**Fig. 4:**
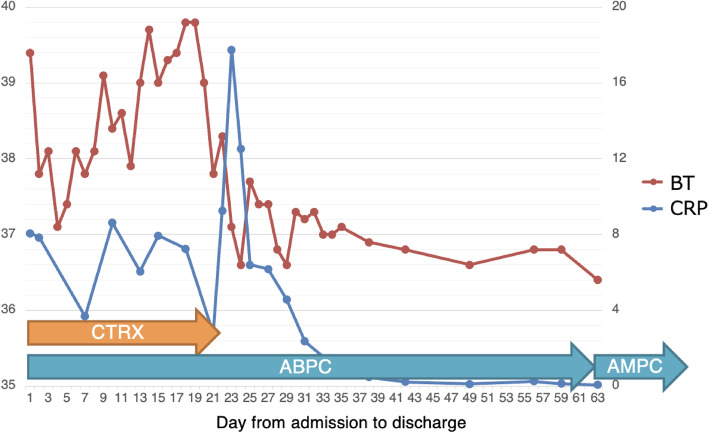
Clinical course of the case. BT, body temperature; CRP, C-reactive protein; CTRX, ceftriaxone; ABPC, ampicillin; AMPC, amoxicillin

**Fig. 5:**
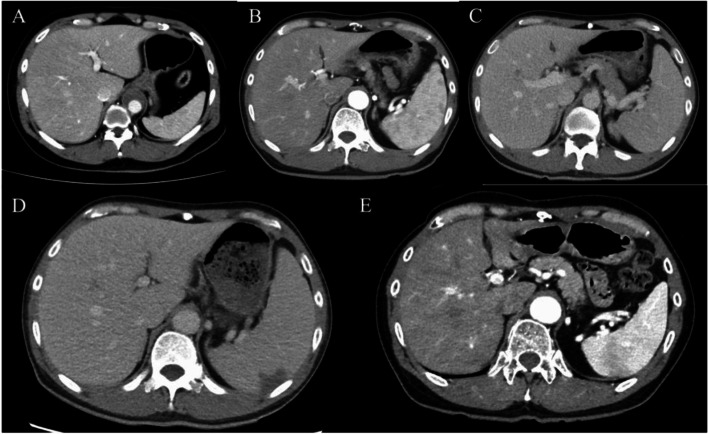
Spleen size followed up with computed tomography. Computed tomography revealed gradual enlargement of the spleen but improvement in spleen size after aortic graft replacement. **A** admission due to aortic dissection; **B** 7 months after surgery; **C** 13 months after surgery; **D** admission due to aortic graft infection 14 months after surgery; **E **5 months after aortic graft replacement

Because meeting the modified Duke criteria was possible, we suspected aortic graft infection with infective endocarditis, and intravenous ampicillin (2 g every 4 h) and ceftriaxone (2 g every 12 h) were initiated. Blood culture identified *Enterococcus faecalis* (*E. faecalis*) by matrix-assisted laser desorption/ionization time-of-flight mass spectrometry (MALDI-TOF Biotyper, Bruker Daltonics). Transthoracic and transesophageal echocardiography revealed no vegetation. Positron emission tomography (PET)-CT on the 10^th^ day showed hyperaccumulation around the aortic graft and spleen (Fig. 2). We finally diagnosed aortic graft infection due to *E. faecali*, and the cardiac surgeon performed aortic arch replacement on the 20^th^ day. After surgery, we de-escalated antibiotics to ampicillin monotherapy. The fever resolved on postoperative day 2 (the 22nd day from admission), and *E. faecalis* was identified in the tissue culture of the graft (Fig. 3). After six weeks of intravenous ampicillin monotherapy, his clinical condition improved, and he was discharged on postoperative day 44 (the 64^th^ day from admission) and switched to oral amoxicillin for three months (Fig. 4). After a half-year follow-up, the size of the splenomegaly decreased (Fig. 5), and there was no relapse after antibiotic therapy was completed.

## Discussion and conclusion

We experienced a case of splenomegaly with splenic infarction caused by AGI due to *E. faecalis*. In this case, the main differential diagnosis was a foreign body reaction. Although the J-graft used in the patient potentially have the possibility, it has been improved and is believed to have fewer the reaction [5]. In addition, if the coating material induces an inflammatory response, the inflammatory response is thought to disappear when the coating material as an antigen is degraded, absorbed, and removed, although this varies depending on the material. The absorption of the coating material is 2 to 4 weeks for gelatin and 6 weeks to 3 months for collagen [6]. In this case, the same type of stent as the first surgery was used, but no clinical findings to date indicate a foreign body reaction. These clinical findings not only before but after the radical surgery supported our diagnosis of aortic graft infection.

AGI is rare, and the incidence reported in the literature is between 0.5% and 6%, although it depends on procedure types [7]. In addition, a search using the keywords “splenomegaly” and “bacteremia” in the electronic databases PubMed, Embase, and Ichushi by March 31, 2022 (Additinal file 1) revealed no reports of splenomegaly due to intravascular graft infection. Thus, we found that this is the first reported case of splenomegaly with splenic infarction due to AGI. In general, the causes of splenomegaly include hepatic, hematologic, infectious, autoimmune, vascular, and malignant diseases [8]. In this case, there were no causes of splenomegaly such as liver, autoimmune, or hematological diseases other than graft infection. In addition, this was also supported by the improvement in splenomegaly after treatment of the infection.

The mechanism of splenomegaly in AGI is not clear yet, but that in infectious endocarditis might be a clue. In infective endocarditis, the association between embolism, thrombus, splenic infarction, and splenomegaly has been previously reported [2–4, 9]. Several case reports of infective endocarditis and pacemaker infection combined with splenomegaly have been reported [10, 11]. Splenic infarction and splenomegaly are associated with septic embolic inflammation in the former and hyperplasia of lymphoid follicles and increased reticuloendothelial cells in the latter [12]. In the present case, although different from infective endocarditis, there were splenic findings suggesting bacteremia and septic embolic inflammation. A similar condition could have led to splenic infarction and splenomegaly.

There is no gold standard for the diagnosis of AGI; therefore, splenomegaly might be helpful in future diagnosis. The principles for diagnosis of AGI include the following: (1) index of suspicion; (2) recognition of the differences in clinical presentations of VGI; (3) time of onset postoperatively; (4) physical findings; (5) laboratory test results, including cultures of blood, purulent material from a draining sinus, or aspirates of perigraft fluid and surgical specimens; and (6) imaging [13]. Accordingly, Lyons et al. proposed dividing the criteria into clinical/surgical, radiologic, and laboratory categories, with major/minor criteria in each category [14]. The diagnosis of AGI is proposed when at least one major/minor criterion is met in addition to one major criterion in another category. The major and minor criteria in radiology include CT and PET-CT findings, respectively, and it has recently been reported that PET-CT may be more sensitive than CT [15]. The study reported that PET/CT had a sensitivity of 93% and specificity of 91% [16]. Technetium-99 m/indium-111-labeled leukocyte imaging is also reported to be helpful for diagnosis [17], with a sensitivity approaching 100% [12]. However, these tests have issues of false positivity [17, 18] and are sometimes difficult to access. Gallium 67 (Ga) scintigraphy has been previously used to detect VGI; however, the sensitivity was not significantly different between CT-scan and 67-Ga imaging [19]. The radiological tests are helpful, but the diagnosis is still made in combination with findings in other categories. On the other hand, the signs and symptoms of AGI are not specific, including fever and chills. However, if AGI involves the aortic root, they can be similar to those of infective endocarditis (IE), such as heart failure and murmur, but not in all cases of AGI. Moreover, a microbiological investigation that consists of laboratory categories is often difficult because of the anatomical location of the vascular graft. Therefore, splenomegaly, which can be examined at the bedside, may facilitate the diagnosis, even though it cannot confirm it.

Finally, the candidates for the treatment of graft infections include surgical drainage and antibiotics [2]. One small case series proposed that splenectomy for endocarditis was complicated by splenic abscess and persistent bacteremia [20]. However, surgery is more invasive, with a reported mortality rate of 18%–30% [2]. Moreover, splenectomy itself poses a higher risk of subsequent overwhelming post-splenectomy infection due to encapsulated bacteria, such as Streptococcus pneumoniae and Haemophilus influenzae [21]. In our case, the patient failed to respond to the antibiotic therapy alone, and surgical intervention without splenectomy was performed to control infection. The size of his spleen decreased after graft replacement and antibiotic therapy, and he completely recovered without splenectomy.

Splenomegaly and splenic infarction are rare, but they could be complicated by AGI. While the diagnosis of graft infections is challenging, these findings could be helpful for its diagnosis.

## Supplementary Information

**Additional file 1.**

## Data Availability

Not applicable.

## Abbreviations

CT: Computed tomography
CRP: C-reactive protein
PET: Positron emission tomography
