# Supplementary material for: First case report of splenomegaly with splenic infarction due to aortic graft infection
Source: BMC Cardiovasc Disord. 2023 May 5;23:237. doi: 10.1186/s12872-023-03259-y (PMC10161471; doi:10.1186/s12872-023-03259-y)
Supplement: Supplementary file 1 — Additional file 1. [file 12872_2023_3259_MOESM1_ESM.zip › Splenomegaly_Embase_ESM.pdf]

SEARCH (((('splenomegaly'/exp OR splenomegaly) OR 'enlarged spleen') AND ('bacteremia'/exp
----

Title	Source	Abstract	Embase L	Open URL Link
Kinetics o	Pathogens	Abortion a	https://w	http://xz9wr2yb6e.search.serialssolutions.com/?sid=
The diagn	Clinical Jo	History: 2(	https://w	http://xz9wr2yb6e.search.serialssolutions.com/?sid=
Group B s	Journal of	Case Repo	https://w	http://xz9wr2yb6e.search.serialssolutions.com/?sid=
Endotipsit	Journal of	Recurrent	https://w	http://xz9wr2yb6e.search.serialssolutions.com/?sid=
Rare but F	Clinical Pa	Diffuse la	https://w	http://xz9wr2yb6e.search.serialssolutions.com/?sid=
Highlights	Clinical A(		https://w	http://xz9wr2yb6e.search.serialssolutions.com/?sid=
Rectal car	IDCases (	Schistoso	https://w	http://xz9wr2yb6e.search.serialssolutions.com/?sid=
Telangiec	Allergy, As	Backgrour	https://w	http://xz9wr2yb6e.search.serialssolutions.com/?sid=
The first b	Pathogens	Infectious	https://w	http://xz9wr2yb6e.search.serialssolutions.com/?sid=
A case of	Journal of	A 52-year-	https://w	http://xz9wr2yb6e.search.serialssolutions.com/?sid=
Contempo	Open Foru	Backgrour	https://w	http://xz9wr2yb6e.search.serialssolutions.com/?sid=
Brucellosi	Open Foru	Automate(	https://w	http://xz9wr2yb6e.search.serialssolutions.com/?sid=
What caus	American	Introducti(	https://w	http://xz9wr2yb6e.search.serialssolutions.com/?sid=
The conne	Italian Jou	Backgrour	https://w	http://xz9wr2yb6e.search.serialssolutions.com/?sid=
From scia	Italian Jou	We descri	https://w	http://xz9wr2yb6e.search.serialssolutions.com/?sid=
Cytomega	Infectious	Cytomega	https://w	http://xz9wr2yb6e.search.serialssolutions.com/?sid=
Curing tw	Pediatric I	Backgrour	https://w	http://xz9wr2yb6e.search.serialssolutions.com/?sid=
Atypical a	Journal of	Scrub typh	https://w	http://xz9wr2yb6e.search.serialssolutions.com/?sid=
Flood sync	Radiology	Flood sync	https://w	http://xz9wr2yb6e.search.serialssolutions.com/?sid=
Failure of	Infection a	Immunoco	https://w	http://xz9wr2yb6e.search.serialssolutions.com/?sid=
A case of	IDCases (	We preser	https://w	http://xz9wr2yb6e.search.serialssolutions.com/?sid=
Identificat	Journal of	Case sum	https://w	http://xz9wr2yb6e.search.serialssolutions.com/?sid=
Acute bru(	Infezioni ir	Brucellosi	https://w	http://xz9wr2yb6e.search.serialssolutions.com/?sid=
An atypica	IDCases (	Lemierre s	https://w	http://xz9wr2yb6e.search.serialssolutions.com/?sid=
Inflammat	Medical R	Inflammat	https://w	http://xz9wr2yb6e.search.serialssolutions.com/?sid=
Group b st	Journal of	LEARNIN(	https://w	http://xz9wr2yb6e.search.serialssolutions.com/?sid=
A rare cas	Journal of	Introducti(	https://w	http://xz9wr2yb6e.search.serialssolutions.com/?sid=
CLOSTRII	Journal of	Case Repo	https://w	http://xz9wr2yb6e.search.serialssolutions.com/?sid=
Pitfall of e	American	INTRODU	https://w	http://xz9wr2yb6e.search.serialssolutions.com/?sid=
A rapidly f	American	INTRODU	https://w	http://xz9wr2yb6e.search.serialssolutions.com/?sid=
Hematolo	Cocuk Ent	Objective:	https://w	http://xz9wr2yb6e.search.serialssolutions.com/?sid=
18-Fluorir	Indian Jou	Melioidos	https://w	http://xz9wr2yb6e.search.serialssolutions.com/?sid=
CLL2-give	HemaSphe	Backgrour	https://w	http://xz9wr2yb6e.search.serialssolutions.com/?sid=
Salmonell	Cocuk Ent	Salmonell	https://w	http://xz9wr2yb6e.search.serialssolutions.com/?sid=
Etiologies	Asian Pac	Objective:	https://w	http://xz9wr2yb6e.search.serialssolutions.com/?sid=
A single-c	Open Foru	Backgrour	https://w	http://xz9wr2yb6e.search.serialssolutions.com/?sid=

A classic	IDCases (	Acute bac	https://w	http://xz9wr2yb6e.search.serialssolutions.com/?sid
Neonatal	Pakistan	Objective:	https://w	http://xz9wr2yb6e.search.serialssolutions.com/?sid
Synchronc	Case Repo	Synchronc	https://w	http://xz9wr2yb6e.search.serialssolutions.com/?sid
The devel	ecancerm	Secondary	https://w	http://xz9wr2yb6e.search.serialssolutions.com/?sid
Fusobacte	Acta Clini	Lemierre	https://w	http://xz9wr2yb6e.search.serialssolutions.com/?sid
Atypical ir	Pediatric	Purpose:	https://w	http://xz9wr2yb6e.search.serialssolutions.com/?sid
DISSEMIN	Chest (20	SESSION	https://w	http://xz9wr2yb6e.search.serialssolutions.com/?sid
Multiple li	American	INTRODU	https://w	http://xz9wr2yb6e.search.serialssolutions.com/?sid
An attack	American	INTRODU	https://w	http://xz9wr2yb6e.search.serialssolutions.com/?sid
HO-1 med	Infection (	Introducti	https://w	http://xz9wr2yb6e.search.serialssolutions.com/?sid
Stay the c	Journal of	Learning (	https://w	http://xz9wr2yb6e.search.serialssolutions.com/?sid
Streptoco	Italian Jou	Backgrour	https://w	http://xz9wr2yb6e.search.serialssolutions.com/?sid
Severe ba	American	Introducti	https://w	http://xz9wr2yb6e.search.serialssolutions.com/?sid
Late-onse	Indian Jou	A 57-year-	https://w	http://xz9wr2yb6e.search.serialssolutions.com/?sid
A case of	Cocuk Ent	Dengue fe	https://w	http://xz9wr2yb6e.search.serialssolutions.com/?sid
Neutrophi	Clinical C	Here, we	https://w	http://xz9wr2yb6e.search.serialssolutions.com/?sid
Prolonged	Iranian Jou	Introducti	https://w	http://xz9wr2yb6e.search.serialssolutions.com/?sid
To cut or	Journal of	Case repo	https://w	http://xz9wr2yb6e.search.serialssolutions.com/?sid
Staphyloc	IDCases (	Staphyloc	https://w	http://xz9wr2yb6e.search.serialssolutions.com/?sid
A case of	LymphoSi	Backgrour	https://w	http://xz9wr2yb6e.search.serialssolutions.com/?sid
Use of PE	ecancerm	Hepatospl	https://w	http://xz9wr2yb6e.search.serialssolutions.com/?sid
Dissemina	Journal of	Infantile d	https://w	http://xz9wr2yb6e.search.serialssolutions.com/?sid
'Blastoid'	Molecular	Burkitt lyn	https://w	http://xz9wr2yb6e.search.serialssolutions.com/?sid
A rare cas	Italian Jou	Introducti	https://w	http://xz9wr2yb6e.search.serialssolutions.com/?sid
Babesia m	Frontiers	Babesia m	https://w	http://xz9wr2yb6e.search.serialssolutions.com/?sid
Laparosco	Internatio	Backgrour	https://w	http://xz9wr2yb6e.search.serialssolutions.com/?sid
Fulminant	Journal of	Case Pres	https://w	http://xz9wr2yb6e.search.serialssolutions.com/?sid
Driven bat	Journal of	Case Pres	https://w	http://xz9wr2yb6e.search.serialssolutions.com/?sid
Hicc-up th	Journal of	Case Pres	https://w	http://xz9wr2yb6e.search.serialssolutions.com/?sid
Recurrent	Journal of	Lactobaci	https://w	http://xz9wr2yb6e.search.serialssolutions.com/?sid
S. sanguis	Internatio	Backgrour	https://w	http://xz9wr2yb6e.search.serialssolutions.com/?sid
Multiple h	American	Fusobacte	https://w	http://xz9wr2yb6e.search.serialssolutions.com/?sid
Anterior a	American	A 53-year-	https://w	http://xz9wr2yb6e.search.serialssolutions.com/?sid
Inflamed l	American	Hemophag	https://w	http://xz9wr2yb6e.search.serialssolutions.com/?sid
Hepatospl	American	Hepatospl	https://w	http://xz9wr2yb6e.search.serialssolutions.com/?sid
What is di	Open Foru	Backgrour	https://w	http://xz9wr2yb6e.search.serialssolutions.com/?sid
What is di	Open Foru	Backgrour	https://w	http://xz9wr2yb6e.search.serialssolutions.com/?sid
Spontaned	Hepatolog	Methods:	https://w	http://xz9wr2yb6e.search.serialssolutions.com/?sid
Scarlet fe	London Jo	There has	https://w	http://xz9wr2yb6e.search.serialssolutions.com/?sid
Clinical cl	Blood (201	Objective:	https://w	http://xz9wr2yb6e.search.serialssolutions.com/?sid

The masq	Indian Jou		https://w	http://xz9wr2yb6e.search.serialssolutions.com/?sid
Hemophag	Kuwait M	Nonimmur	https://w	http://xz9wr2yb6e.search.serialssolutions.com/?sid
A rare pai	Journal of	LEARNIN(	https://w	http://xz9wr2yb6e.search.serialssolutions.com/?sid
As clean a	Journal of	LEARNIN(	https://w	http://xz9wr2yb6e.search.serialssolutions.com/?sid
Invasive li	Journal of	Case pres	https://w	http://xz9wr2yb6e.search.serialssolutions.com/?sid
Fusobacte	Case Repo	We report	https://w	http://xz9wr2yb6e.search.serialssolutions.com/?sid
Dysgamm	Annals of	With great	https://w	http://xz9wr2yb6e.search.serialssolutions.com/?sid
Bivalvular	American	Introducti	https://w	http://xz9wr2yb6e.search.serialssolutions.com/?sid
Sepsis du	Journal of	LEARNIN(	https://w	http://xz9wr2yb6e.search.serialssolutions.com/?sid
Deaths by	Laborator	Backgrour	https://w	http://xz9wr2yb6e.search.serialssolutions.com/?sid
Epidemiol	Vector-Bo	Backgrour	https://w	http://xz9wr2yb6e.search.serialssolutions.com/?sid
Challenge	Open Foru	The diagn	https://w	http://xz9wr2yb6e.search.serialssolutions.com/?sid
Gastrointe	IDCases (	Fusobacte	https://w	http://xz9wr2yb6e.search.serialssolutions.com/?sid
Bridging t	Journal of	Long-term	https://w	http://xz9wr2yb6e.search.serialssolutions.com/?sid
Rates of i	Blood (201	Ruxolitinib	https://w	http://xz9wr2yb6e.search.serialssolutions.com/?sid
A case of	American	Introducti	https://w	http://xz9wr2yb6e.search.serialssolutions.com/?sid
Fever of u	Journal of	Although	https://w	http://xz9wr2yb6e.search.serialssolutions.com/?sid
Pseudomo	IDCases (	Infections	https://w	http://xz9wr2yb6e.search.serialssolutions.com/?sid
Case repo	Internation	INTRODU	https://w	http://xz9wr2yb6e.search.serialssolutions.com/?sid
Hemophag	Critical Ca	Introducti	https://w	http://xz9wr2yb6e.search.serialssolutions.com/?sid
Sequelae	Annals of	Hyper-IgM	https://w	http://xz9wr2yb6e.search.serialssolutions.com/?sid
Neutroper	Blood (201	Hypoglyce	https://w	http://xz9wr2yb6e.search.serialssolutions.com/?sid
Obliterativ	American	Introducti	https://w	http://xz9wr2yb6e.search.serialssolutions.com/?sid
A case of	American	Introducti	https://w	http://xz9wr2yb6e.search.serialssolutions.com/?sid
Infective e	Annals of	Colonosco	https://w	http://xz9wr2yb6e.search.serialssolutions.com/?sid
Malaria in	Mediterra	This revie	https://w	http://xz9wr2yb6e.search.serialssolutions.com/?sid
Clinical ch	AIDS Rese	Objective:	https://w	http://xz9wr2yb6e.search.serialssolutions.com/?sid
Meningoc	Archives o	Previously	https://w	http://xz9wr2yb6e.search.serialssolutions.com/?sid
The develo	Bone Marr	The activa	https://w	http://xz9wr2yb6e.search.serialssolutions.com/?sid
A rare cau	Balkan Me	Although	https://w	http://xz9wr2yb6e.search.serialssolutions.com/?sid
Lemierre's	Chest (20	INTRODU	https://w	http://xz9wr2yb6e.search.serialssolutions.com/?sid
Successfu	Bone Marr	Aim: Cons	https://w	http://xz9wr2yb6e.search.serialssolutions.com/?sid
Two in on	Critical Ca	Backgrour	https://w	http://xz9wr2yb6e.search.serialssolutions.com/?sid
Bendamus	Haematolo	Backgrour	https://w	http://xz9wr2yb6e.search.serialssolutions.com/?sid
Fungus ar	Journal of	MAS is a	https://w	http://xz9wr2yb6e.search.serialssolutions.com/?sid
Splenic ab	Journal of	Case Pres	https://w	http://xz9wr2yb6e.search.serialssolutions.com/?sid
A cough th	Journal of	Case Repo	https://w	http://xz9wr2yb6e.search.serialssolutions.com/?sid
Endocardi	Journal fur		https://w	http://xz9wr2yb6e.search.serialssolutions.com/?sid
Emerging	Singapore	Leuconos	https://w	http://xz9wr2yb6e.search.serialssolutions.com/?sid
Streptoco	Infectious	A 49-year-	https://w	http://xz9wr2yb6e.search.serialssolutions.com/?sid

Diagnostic	Journal of	Introductio	https://wv	http://xz9wr2yb6e.search.serialssolutions.com/?sid=
Infective e	Journal of	Introductio	https://wv	http://xz9wr2yb6e.search.serialssolutions.com/?sid=
Clinical m	Enfermeda	The clinica	https://wv	http://xz9wr2yb6e.search.serialssolutions.com/?sid=
Fever, dia	Enfermeda	Fever, dia	https://wv	http://xz9wr2yb6e.search.serialssolutions.com/?sid=
Role of pro	Indian Jou	Antimicrob	https://wv	http://xz9wr2yb6e.search.serialssolutions.com/?sid=
Salmonella	Chirurgisc	Backgroun	https://wv	http://xz9wr2yb6e.search.serialssolutions.com/?sid=
A pain in t	Internation	Objective:	https://wv	http://xz9wr2yb6e.search.serialssolutions.com/?sid=
Cladribine	Best Pract	Cladribine	https://wv	http://xz9wr2yb6e.search.serialssolutions.com/?sid=
Bacterial p	Hepatolog	Hepatic IV	https://wv	http://xz9wr2yb6e.search.serialssolutions.com/?sid=
Congenita	Internation	Congenita	https://wv	http://xz9wr2yb6e.search.serialssolutions.com/?sid=

OR bacteremia OR bacteraemia)) NOT ((('splenomegaly'/exp OR splenomegaly) OR 'enlarged sp

=EMBASE&issn=20760817&id=doi:10.3390%2Fpathogens11030279&atitle=Kinetics+of+Place
=EMBASE&issn=15363724&id=doi:10.1097%2FJSM.0000000000001017&atitle=The+diagnosis
=EMBASE&issn=17088267&id=doi:10.1136%2Fjim-2022-SRMC.83&atitle=Group+B+streptocc
=EMBASE&issn=22133453&id=doi:10.1016%2Fj.jceh.2021.02.009&atitle=Endotipsitis%3A+An
=EMBASE&issn=2632010X&id=doi:10.1177%2F2632010X211070774&atitle=Rare+but+Potenti
=EMBASE&issn=15430790&id=doi:&atitle=Highlights+in+Graft-vs-Host+Disease+From+the
=EMBASE&issn=22142509&id=doi:10.1016%2Fj.idcr.2022.e01383&atitle=Rectal+carcinoma+a
=EMBASE&issn=17101492&id=doi:10.1186%2Fs13223-022-00647-5&atitle=Telangiectasia+m
=EMBASE&issn=20760817&id=doi:10.3390%2Fpathogens10121580&atitle=The+first+bacteria
=EMBASE&issn=20428812&id=doi:10.1093%2Fjscr%2Frjab541&atitle=A+case+of+splenic+ru
=EMBASE&issn=23288957&id=doi:10.1093%2Fofid%2Fofab466.895&atitle=Contemporary+Cli
=EMBASE&issn=23288957&id=doi:10.1093%2Fofid%2Fofab473&atitle=Brucellosis+initially+m
=EMBASE&issn=15720241&id=doi:10.14309%2F01.ajg.0000788080.33147.74&atitle=What+cau
=EMBASE&issn=18779352&id=doi:&atitle=The+connection+between+rheumatoid+factor%2C
=EMBASE&issn=18779352&id=doi:10.4081%2Fitjm.2021.1386&atitle=From+sciatica+pain+to
=EMBASE&issn=15369943&id=doi:10.1097%2FIPC.0000000000000980&atitle=Cytomegaloviru
=EMBASE&issn=15455017&id=doi:10.1002%2Fpbc.29060&atitle=Curing+two+diseases+with
=EMBASE&issn=0973709X&id=doi:10.7860%2FJCDR%2F2021%2F48310.14744&atitle=Atypica
=EMBASE&issn=19300433&id=doi:10.1016%2Fj.radcr.2020.10.045&atitle=Flood+syndrome+m
=EMBASE&issn=10985522&id=doi:10.1128%2FIAI.00417-20&atitle=Failure+of+CD4+T+cell-
=EMBASE&issn=22142509&id=doi:10.1016%2Fj.idcr.2021.e01045&atitle=A+case+of+Gemella
=EMBASE&issn=20551169&id=doi:10.1177%2F20551169211012346&atitle=Identification+of+
=EMBASE&issn=11249390&id=doi:10.53854%2Fliim-2903-19&atitle=Acute+brucellosis+asso
=EMBASE&issn=22142509&id=doi:10.1016%2Fj.idcr.2021.e01314&atitle=An+atypical+presen
=EMBASE&issn=21974187&id=doi:10.1007%2F978-3-030-39021-1_2&atitle=Inflammatory+liv
=EMBASE&issn=15251497&id=doi:10.1007%2Fs11606-021-06830-5&atitle=Group+b+streptoc
=EMBASE&issn=15333450&id=doi:&atitle=A+rare+case+of+evans+syndrome+with+systemi
=EMBASE&issn=17088267&id=doi:10.1136%2Fjim-2022-WRMC.409&atitle=CLOSTRIDIUM+P
=EMBASE&issn=15720241&id=doi:10.14309%2F01.ajg.0000715364.23361.0f&atitle=Pitfall+of
=EMBASE&issn=15720241&id=doi:10.14309%2F01.ajg.0000711856.91947.2d&atitle=A+rapidly
=EMBASE&issn=13085271&id=doi:10.5578%2Fced.202046&atitle=Hematological+findings+in
=EMBASE&issn=09740244&id=doi:10.4103%2Fijnm.IJNM_15_20&atitle=18-Fluorine-fluorodeo
=EMBASE&issn=25729241&id=doi:10.1097%2FHS9.0000000000000404&atitle=CLL2-give%2C
=EMBASE&issn=13085271&id=doi:10.5578%2Fced.68699&atitle=Salmonella+ser.+Typhimuriu
=EMBASE&issn=19957645&id=doi:10.4103%2F1995-7645.278096&atitle=Etiologies+of+tropic
=EMBASE&issn=23288957&id=doi:10.1093%2Fofid%2Fofaa050&atitle=A+single-center+pros

=EMBASE&issn=22142509&id=doi:10.1016%2Fj.idcr.2020.e00701&atitle=A+classic+and+fata
=EMBASE&issn=03044904&id=doi:&atitle=Neonatal+sepsis-An+etiological+study&stitle=Pa
=EMBASE&issn=20906714&id=doi:10.1155%2F2020%2F8888829&atitle=Synchronous+Occurr
=EMBASE&issn=17546605&id=doi:10.3332%2FECANCER.2020.1011&atitle=The+development
=EMBASE&issn=22953337&id=doi:10.1080%2F17843286.2019.1687975&atitle=Fusobacterium
=EMBASE&issn=15455017&id=doi:10.1002%2Fpbc.28077&atitle=Atypical+intracranial+lesion
=EMBASE&issn=19313543&id=doi:10.1016%2Fj.chest.2019.08.610&atitle=DISSEMINATED+
=EMBASE&issn=15720241&id=doi:10.14309%2F01.ajg.0000598640.17372.26&atitle=Multiple+
=EMBASE&issn=15720241&id=doi:10.14309%2F01.ajg.0000597092.77416.67&atitle=An+attac
=EMBASE&issn=14390973&id=doi:10.1007%2Fs15010-019-01341-2&atitle=HO-1+mediates+
=EMBASE&issn=15251497&id=doi:10.1007%2F11606.1525-1497&atitle=Stay+the+course+an
=EMBASE&issn=18779352&id=doi:10.4081%2Fitjm.2019.s2&atitle=Streptococcus+gallolyticu
=EMBASE&issn=15354970&id=doi:&atitle=Severe+babesiosis+with+low+levels+of+parasite
=EMBASE&issn=22120025&id=doi:10.4103%2Fijot.ijot_56_18&atitle=Late-onset+posttranspl
=EMBASE&issn=13085271&id=doi:10.5578%2Fced.67500&atitle=A+case+of+dengue+fever+
=EMBASE&issn=20500904&id=doi:10.1002%2Fccr3.1932&atitle=Neutrophilic+eccrine+hidrad
=EMBASE&issn=20082150&id=doi:10.5812%2Fijp.69170&atitle=Prolonged+fever+and+intrave
=EMBASE&issn=17088267&id=doi:10.1136%2Fjim-2018-000974.254&atitle=To+cut+or+not+
=EMBASE&issn=22142509&id=doi:10.1016%2Fj.idcr.2019.e00656&atitle=Staphylococcus+pas
=EMBASE&issn=22925945&id=doi:10.14785%2Flymphosign-2018-0014&atitle=A+case+of+K
=EMBASE&issn=17546605&id=doi:10.3332%2Fecancer.2018.872&atitle=Use+of+PEG-aspara
=EMBASE&issn=18655785&id=doi:10.1007%2Fs12308-018-0327-1&atitle=Disseminated+BC
=EMBASE&issn=20499469&id=doi:10.3892%2Fmco.2018.1585&atitle=%E2%80%98Blastoid%E
=EMBASE&issn=18779352&id=doi:10.4081%2Fitjm.2018.s2&atitle=A+rare+case+of+sepsis+
=EMBASE&issn=1664302X&id=doi:10.3389%2Ffmicb.2018.00085&atitle=Babesia+microti+inf
=EMBASE&issn=22102612&id=doi:10.1016%2Fj.ijscr.2018.01.017&atitle=Laparoscopic+splen
=EMBASE&issn=15535606&id=doi:&atitle=Fulminant+gram-negative+septic+shock+due+to+
=EMBASE&issn=15535606&id=doi:&atitle=Driven+batty%3A+Seeking+a+diagnosis+for+a+p
=EMBASE&issn=15535606&id=doi:&atitle=Hicc-up+the+ante%3A+A+catastrophic+case+of
=EMBASE&issn=23247096&id=doi:10.1177%2F2324709617744233&atitle=Recurrent+Lactoba
=EMBASE&issn=18727913&id=doi:&atitle=S.+sanguis+bacteremia%2C+scrub+typhus+and+
=EMBASE&issn=15720241&id=doi:10.1038%2Fajg.2017.321&atitle=Multiple+hepatic+absces
=EMBASE&issn=15720241&id=doi:10.1038%2Fajg.2017.321&atitle=Anterior+abdominal+wall
=EMBASE&issn=15720241&id=doi:10.1038%2Fajg.2017.321&atitle=Inflamed+liver%3A+A+cas
=EMBASE&issn=15720241&id=doi:10.1038%2Fajg.2017.321&atitle=Hepatosplenic+T-cell+lym
=EMBASE&issn=23288957&id=doi:10.1093%2Fofid%2Fofx163.151&atitle=What+is+different+
=EMBASE&issn=23288957&id=doi:10.1093%2Fofid%2Fofxl63.151&atitle=What+is+different+
=EMBASE&issn=19360533&id=doi:10.1007%2Fs12072-016-9783-9&atitle=Spontaneous+bact
=EMBASE&issn=17571480&id=doi:10.1080%2F17571472.2017.1365677&atitle=Scarlet+fever%
=EMBASE&issn=15280020&id=doi:&atitle=Clinical+characterization+of+staphylococcus+sep

=EMBASE&issn=09752129&id=doi:10.4103%2F0971-5851.195728&atitle=The+masquerading+
=EMBASE&issn=00235776&id=doi:&atitle=Hemophagocytic+lymphohistiocytosis+in+a+newb
=EMBASE&issn=15251497&id=doi:&atitle=A+rare+pain+in+a+common+scenario&stitle=J.+(
=EMBASE&issn=15251497&id=doi:&atitle=As+clean+as+the+cow+that+calves%3A+A+case
=EMBASE&issn=17088267&id=doi:10.1136%2Fjim-2016-000120.55&atitle=Invasive+liver+abs
=EMBASE&issn=16879635&id=doi:10.1155%2F2016%2F3608346&atitle=Fusobacterium+necro
=EMBASE&issn=10811206&id=doi:&atitle=Dysgammaglobulinemia%2C+lymphoproliferation%2
=EMBASE&issn=1073449X&id=doi:&atitle=Bivalvular+endocarditis+due+to+methicillin-resist
=EMBASE&issn=08848734&id=doi:&atitle=Sepsis+due+to+listeria+bacteremia+as+initial+p
=EMBASE&issn=00236837&id=doi:10.1038%2Flabinvest.2015.3&atitle=Deaths+by+dengue+fe
=EMBASE&issn=15577759&id=doi:10.1089%2Fvbz.2014.1726&atitle=Epidemiological%2C+dia
=EMBASE&issn=23288957&id=doi:10.1093%2Fofid%2Fofv025&atitle=Challenges+in+the+ma
=EMBASE&issn=22142509&id=doi:10.1016%2Fj.idcr.2015.07.001&atitle=Gastrointestinal+vari
=EMBASE&issn=20082371&id=doi:&atitle=Bridging+to+heart+transplantation+from+the+bive
=EMBASE&issn=00064971&id=doi:&atitle=Rates+of+infection+in+myelofibrosis+patients+tr
=EMBASE&issn=1073449X&id=doi:&atitle=A+case+of+hemophagocytic+lymphohistiocytosis+
=EMBASE&issn=13057693&id=doi:10.3233%2FJPI-140408&atitle=Fever+of+unknown+origin+
=EMBASE&issn=22142509&id=doi:10.1016%2Fj.idcr.2014.09.001&atitle=Pseudomonas+bacte
=EMBASE&issn=22102612&id=doi:10.1016%2Fj.ijscr.2014.10.069&atitle=Case+report+of+rar
=EMBASE&issn=00903493&id=doi:10.1097%2F01.ccm.0000440448.09992.41&atitle=Hemopha
=EMBASE&issn=10811206&id=doi:&atitle=Sequelae+of+delayed+diagnosis+of+hyper-IgM+s
=EMBASE&issn=00064971&id=doi:&atitle=Neutropenia+in+glycogen+storage+disease+1b+%
=EMBASE&issn=00029270&id=doi:10.1038%2Fajg.2013.267&atitle=Obliterative+portal+venop
=EMBASE&issn=1073449X&id=doi:&atitle=A+case+of+mediastinitis+following+endobronchia
=EMBASE&issn=11087471&id=doi:&atitle=Infective+endocarditis+and+infected+aneurysm+c
=EMBASE&issn=20353006&id=doi:10.4084%2FMJHID.2012.073&atitle=Malaria+in+children&s
=EMBASE&issn=17426405&id=doi:10.1186%2F1742-6405-9-24&atitle=Clinical+characteristic
=EMBASE&issn=00039888&id=doi:10.1136%2Farchdischild-2012-301885.24&atitle=Meningoc
=EMBASE&issn=02683369&id=doi:10.1038%2Fbmt.2012.37&atitle=The+development+of+hae
=EMBASE&issn=21463131&id=doi:10.5152%2Fbalkanmedj.2012.053&atitle=A+rare+cause+of
=EMBASE&issn=00123692&id=doi:10.1378%2Fchest.1118551&atitle=Lemierre%27s+disease%
=EMBASE&issn=02683369&id=doi:10.1038%2Fbmt.2011.48&atitle=Successful+unrelated+cor
=EMBASE&issn=00903493&id=doi:10.1097%2F01.ccm.0000390903.16849.8c&atitle=Two+in+
=EMBASE&issn=03906078&id=doi:&atitle=Bendamustine+in+association+with+rituximab+as
=EMBASE&issn=10761608&id=doi:10.1097%2FRHU.0b013e3181db7b64&atitle=Fungus+and+
=EMBASE&issn=15535592&id=doi:10.1002%2Fjhm.709&atitle=Splenic+abscess+-+A+rare+b
=EMBASE&issn=10815589&id=doi:10.231%2FJIM.0b013e3182820c55&atitle=A+cough+that+b
=EMBASE&issn=10240098&id=doi:&atitle=Endocarditis+Update+2009%3A+Annual+meeting+
=EMBASE&issn=02183048&id=doi:&atitle=Emerging+pathogen%3A+Leuconostoc+bacteremia
=EMBASE&issn=10569103&id=doi:10.1097%2FIPC.0b013e31819e30ae&atitle=Streptococcus+

=EMBASE&issn=17521947&id=doi:10.1186%2F1752-1947-2-315&atitle=Diagnostic+difficultie
=EMBASE&issn=17521947&id=doi:10.1186%2F1752-1947-2-143&atitle=Infective+endocarditi
=EMBASE&issn=0213005X&id=doi:10.1157%2F13123262&atitle=Clinical+manifestations+of+
=EMBASE&issn=0213005X&id=doi:10.1157%2F13123267&atitle=Fever%2C+diarrhoea+and+n
=EMBASE&issn=09729607&id=doi:&atitle=Role+of+prophylactic+antimicrobials+in+pediatric
=EMBASE&issn=01779990&id=doi:10.1159%2F000094837&atitle=Salmonella+splenic+absces
=EMBASE&issn=08856265&id=doi:&atitle=A+pain+in+the+neck+can+lead+to+pain+in+the+
=EMBASE&issn=15216926&id=doi:10.1016%2FS1521-6926%2802%2900089-0&atitle=Cladribi
=EMBASE&issn=13866346&id=doi:10.1016%2FS1386-6346%2802%2900018-9&atitle=Bacteria
=EMBASE&issn=10702903&id=doi:&atitle=Congenital+bone+marrow+failure&stitle=Int.+J.+F

leen') AND ('bacteremia'/exp OR bacteremia OR bacteraemia) AND ([medline]/lim OR [preprint]/

ntal+Infection+by+Different+Smooth+Brucella+Strains+in+Mice&stitle=Pathogens&title=Pa
+of+a+sinister+sore+throat+in+this+collegiate+basketball+player+is+no+lay+up&stitle=Cl
occal+bacteremia+possibly+due+to+genitourinary+invasion+from+sexual+encounter%3A+A+
+Underdiagnosed+Complication+of+Transjugular+Intrahepatic+Portosystemic+Shunts&stitle
ally+Fatal+Presentations+of+Diffuse+Large+B-cell+Lymphoma%3A+Leukemic+Phase+or+H
+63rd+American+Society+of+Hematology+Annual+Meeting+and+Exposition&stitle=Clin.+Ad
rising+in+a+patient+with+intestinal+and+hepatic+schistosomiasis+due+to+Schistosoma+
acularis+eruptiva+perstans+with+systemic+involvement%3A+a+case+report&stitle=Allergy+
l+endocarditisdue+to+achromobacter+xylosoxidans+in+a+dog&stitle=Pathogens&title=Path
upture+a+week+after+appendectomy&stitle=J.+Surg.+Case+Rep.&title=Journal+of+Surgical+
nical+Epidemiology+of+Pediatric+Shigella+and+Campylobacter+Infections+in+Houston%2C+
isidentified+as+ochrobactrum+anthropi+Bacteremia%3A+A+case+report+and+review+of+th
used+the+cirrhosis%3F+Cirrhosis+with+normal+liver+biopsy&stitle=Am.+J.+Gastroenterol.&
+heart+and+cancer%3A+A+real+life+scenario&stitle=Ital.+J.+Med.&title=Italian+Journal+o
+a+complex+diagnosis%3A+A+case+of+pyomyositis+and+chronic+lymphocytic+leukemia&s
s+Reactivation+after+Bendamustine-Based+Chemotherapy%3A+A+Case+Report&stitle=Infe
+one+transplant%3A+The+case+of+a+male+infant+with+both+wiskott-aldrich+syndrome+
l+and+severe+manifestations+of+scrub+typhus+in+children%3A+Hiding+without+the+rash
anaged+by+partial+splenic+embolization+and+percutaneous+peritoneal+drainage&stitle=Ra
deficient+hosts+to+control+chronic+nontyphoidal+salmonella+infection+leads+to+exacerbat
+morbillorum+native+valve+endocarditis+and+results+of+in+vitro+susceptibility+testing&s
Streptococcus+suis+in+a+cat+with+endomyocarditis&stitle=J.+Feline+Med.+Surg.+Open+
ciated+with+isolated+splenic+and+left+gastric+artery+vasculitis+and+acute+ischemic+bo
tation+of+lemierre+syndrome+of+urogenital+source&stitle=IDCases&title=IDCases&volume
er+lesions&stitle=Med.+Radiol.&title=Medical+Radiology&volume=&issue=&spage=49&epag
coccus+infective+endocarditis+in+a+non-pregnant+middle+age+woman+with+severe+uteri
c+lupus+erythematosus+and+pulmonary+nocardiosis&stitle=J.+Am.+Soc.+Nephrol.&title=Jo
ARAPUTRIFICUM+in+46-YEAR-OLD+MALE+with+LIVER+DISEASE&stitle=J.+Invest.+Med.
+elevated+ca+19-9&stitle=Am.+J.+Gastroenterol.&title=American+Journal+of+Gastroenterol
+fatal+case+of+fulminant+hepatic+failure+from+streptococcus+salivarius+bacteremia&stit
+children+with+brucellosis&stitle=Cocuk+Enfeksiyon+Derg.&title=Cocuk+Enfeksiyon+Dergi
xyglucose+positron+emission+Tomography-Computed+tomography+in+the+evaluation+of+th
+a+prospective%2C+open-label%2C+multicenter+phase-ii+trial+of+obinutuzumab+%28GA1
m+bacteremia+related+hemophagocytic+lymphohistiocytosis%3A+A+case+report&stitle=Co
al+acute+febrile+illness+in+West+Pahang%2C+Malaysia%3A+A+prospective+observational
ective+cohort+study+on+postsplenectomy+sepsis+and+its+prevention&stitle=Open+Forum

l+case+of+Streptococcus+mutans+subacute+bacterial+endocarditis%3B+A+now+potentiall
k.+Paediatr.+J.&title=Pakistan+Paediatric+Journal&volume=44&issue=4&spage=356&epage=
ence+of+Splenic+Pleomorphic+Mantle+Cell+Lymphoma+and+Esophageal+Adenocarcinoma+
t+of+T-cell+malignancies+in+patients+with+pre-existing+myeloproliferative+neoplasms%3A
+necrophorum+septicemia+with+portal+thrombosis+and+liver+abscesses%3B+an+intra-abc
s+of+HLH+in+a+child&stitle=Pediatr.+Blood+Cancer&title=Pediatric+Blood+and+Cancer&v
IAC+IN+A+YOUNG+IMMUNOCOMPETENT+PATIENT&stitle=Chest&title=Chest&volume=15
liver+abscesses+caused+by+streptococcus+intermedius+bacteremia+in+the+setting+of+a-
k+of+disseminated+MAC&stitle=Am.+J.+Gastroenterol.&title=American+Journal+of+Gastroe
metabolic+adaptation+to+heme+but+not+to+Staphylococcus+aureus+bacteremia&stitle=Url
d+don%27t+put+down+anchors&stitle=J.+Gen.+Intern.+Med.&title=Journal+of+General+Inte
s+subspecies+Pasteurianus+bacteraemia+following+adjuvant+chemotherapy+after+distal+p
mia&stitle=Am.+J.+Respir.+Crit.+Care+Med.&title=American+Journal+of+Respiratory+and+(
ant+lymphoproliferative+disease+in+a+male+kidney+transplant+patient+on+minimal+triple+
complicated+with+trombophlebitis+in+a+child&stitle=Cocuk+Enfeksiyon+Derg.&title=Cocuk
enitis+secondary+to+pegfilgrastim+in+a+patient+with+synovial+sarcoma&stitle=Clin.+Case
enous+immunoglobulin+resistance+in+kawasaki+disease%3A+Should+macrophage+activatio
to+cut%3A+Right-sided+infective+endocarditis+with+persistent+bacteremia&stitle=J.+Inves
teuri+infective+endocarditis%3A+A+case+report&stitle=IDCases&title=IDCases&volume=18
AT6A+mutation+associated+with+immunodeficiency+and+granulomatous+lymphocytic+inters
ginase+in+a+case+of+Hepatosplenic+%CE%B3%CE%B4+T-cell+lymphoma+with+long-term
G-osis+with+haemophagocytosis%2C+tubercular+bacteraemia%2C+and+unusual+haematolog
2%80%99+variant+of+Burkitt+lymphoma+with+additional+partial+1q+tetrasomy&stitle=Mol.
in+an+healthy+young+man&stitle=Ital.+J.+Med.&title=Italian+Journal+of+Medicine&volume=
ection+changes+host+spleen+architecture+and+is+cleared+by+a+Th1+immune+response&
ectomy+for+a+simultaneous+wandering+spleen+along+with+an+ectopic+accessory+spleen
-capnocytophaga+infection&stitle=J.+Hosp.+Med.&title=Journal+of+Hospital+Medicine&volu
lethora+of+findings&stitle=J.+Hosp.+Med.&title=Journal+of+Hospital+Medicine&volume=13
+babesiosis&stitle=J.+Hosp.+Med.&title=Journal+of+Hospital+Medicine&volume=13&issue=
cillus+Bacteremia+in+a+Patient+With+Leukemia&stitle=J.+Investig.+Med.+High+Impact+Ca
leptospirosis+coinfection+complicated+with+Herpes+simplex+virus+replication&stitle=Int.+
ses+secondary+to+fusobacterium+bacteremia&stitle=Am.+J.+Gastroenterol.&title=American
+cellulitis%3A+A+rare+presentation+of+klebsiella+spontaneous+bacterial+peritonitis&stitle=
se+of+hemophagocytic+lymphohistiocytosis+and+colorectal+cancer+in+a+patient+with+rap
nphoma+in+patient+with+Crohn%27s+disease+receiving+immunomodulator+therapy%3A+A+
-when+dealing+with+bacteremic+brucellosis%3F&stitle=Open+Forum+Infect.+Dis.&title=Op
when+dealing+with+bacteremic+brucellosis%3F&stitle=Open+Forum+Infect.+Dis.&title=Ope
erial+peritonitis+with+non-typhoidal+group+D+Salmonella+infection&stitle=Hepatol.+Int.&ti
3A+A+guide+for+general+practitioners&stitle=London+J.+Prim.+Care&title=London+Journa
ticemia-associated+hemophagocytic+lymphohistiocytosis&stitle=Blood&title=Blood&volume=

-splenic+lesion&stitle=Indian+J.+Med.+Paediatr.+Oncol.&title=Indian+Journal+of+Medical+a
orn+infant+presenting+with+nonimmune+hydrops+fetalis%3A+A+case+report&stitle=Kuwai
Gen.+Intern.+Med.&title=Journal+of+General+Internal+Medicine&volume=31&issue=2&spage
e+of+leukocytosis&stitle=J.+Gen.+Intern.+Med.&title=Journal+of+General+Internal+Medicine
cess+syndrome+in+North+America&stitle=J.+Invest.+Med.&title=Journal+of+Investigative+M
ophorum+pharyngitis+complicated+by+Lemierre%27s+syndrome&stitle=Case+Rep.+Med.&tit
2C+and+recurrent+sinopulmonary+infections+associated+with+E1021K+mutation+in+the+p
tant+staphylococcus+aureus+managed+with+daptomycin+and+ceftaroline&stitle=Am.+J.+Re
resentation+of+hodgkin+lymphoma&stitle=J.+Gen.+Intern.+Med.&title=Journal+of+General+
ever+base+on+analysis+of+autopsy+cases&stitle=Lab.+Invest.&title=Laboratory+Investigati
gnostic%2C+clinical%2C+and+therapeutic+aspects+of+brucella+bacteremia+in+children+in
nagement+of+disseminated+progressive+histoplasmosis+in+human+immunodeficiency+virus
ant+of+Lemierre%27s+syndrome+complicating+ruptured+appendicitis&stitle=IDCases&title=
entricular+pulsatile+berlin+heart+EXCOR+assist+device+support+in+a+patient+with+advan
eated+with+ruxolitinib&stitle=Blood&title=Blood&volume=124&issue=21&spage=&epage=&a
+in+the+setting+of+profound+HIV+immunosuppression+and+cryptococcal+bacteremia&stitl
-in+children%3A+A+challenge+persisting+with+advancing+medical+care&stitle=J.+Pediatr.+
remia+as+an+initial+presentation+of+SLE&stitle=IDCases&title=IDCases&volume=1&issue
e+chronic+myelogenous+leukemia+related+multibacterial+splenic+abscess+presenting+wit
gocytic+lymphohistiocytosis+syndrome%28HLH%29+%3A+A+great+sepsis+masquerade&stit
yndrome+as+an+adult+in+absence+of+known+genetic+mutations&stitle=Ann.+Allergy+Ast
%28GSD1b%29&stitle=Blood&title=Blood&volume=122&issue=21&spage=&epage=&aulast=D
athy%3A+A+rare+cause+of+pre-sinusoidal+portal+hypertension&stitle=Am.+J.+Gastroenter
al+ultrasound+guided+transbronchial+needle+aspiration&stitle=Am.+J.+Respir.+Crit.+Care+
of+splenic+artery+post+colonoscopy&stitle=Ann.+Gastroenterol.&title=Annals+of+Gastroent
stitle=Mediterr.+J.+Hematol.+Infect.+Dis.&title=Mediterranean+Journal+of+Hematology+and
cs+and+outcome+of+Penicillium+marneffei+infection+among+HIV-infected+patients+in+nor
occal+sepsis+and+haemophagocytic+lymphohistiocytosis&stitle=Arch.+Dis.+Child.&title=Ar
mophagocytic+syndrome+is+associated+with+high-risk+disease+and+viral+reactivation+an
f+endocarditis%3A+Streptococcus+pyogenes&stitle=Balkan+Med.+J.&title=Balkan+Medical+
%3A+Not+so+forgotten%21&stitle=Chest&title=Chest&volume=140&issue=4&spage=&epage
d+blood+transplantation+in+a+child+with+chronic+eosinophilic+leukaemia+and+constitutic
one-HLH+in+sepsis+and+acute+myelofibrosis&stitle=Crit.+Care+Med.&title=Critical+Care-
s+salvage+therapy+for+splenic+marginal+zone+lymphoma&stitle=Haematologica&title=Haen
virus+as+triggers+of+macrophage+activation+syndrome+%28MAS%29+in+an+adult+without
out+lethal+disease&stitle=J.+Hosp.+Med.&title=Journal+of+Hospital+Medicine&volume=5&is
becomes+an+emergency&stitle=J.+Invest.+Med.&title=Journal+of+Investigative+Medicine&vo
-of+the+Austrian+Society+of+Cardiology%2C+Salzburg%2C+June+3-6%2C+2009&stitle=J.+I
a&stitle=Singapore+Gen.+Hosp.+Proc.&title=Singapore+General+Hospital+Proceedings&volu
-equi+subspecies+zooepidemicus+bacteremia+as+first+manifestation+of+hairy+cell+leuken

es+of+Lactobacillus+casei+bacteraemia+in+immunocompetent+patients%3A+A+case+repor
s+caused+by+Staphylococcus+aureus+in+a+patient+with+atopic+dermatitis%3A+A+case+
HIV+infection+in+distinct+geographical+areas&stitle=Enferm.+Infecc.+Microbiol.+Clin.&titl
eurological+syndrome+in+HIV-infected+immigrants&stitle=Enferm.+Infecc.+Microbiol.+Clin.
+practice&stitle=Indian+J.+Pract.+Pediatr.&title=Indian+Journal+of+Practical+Pediatrics&vo
s+as+an+unusual+complication+of+acute+gastroenteritis&stitle=Chir.+Gastroenterol.+Inte
belly%3A+Lemierre%27s+syndrome&stitle=Int.+Pediatr.&title=International+Pediatrics&volu
ne+in+the+treatment+of+hairy-cell+leukaemia&stitle=Best+Pract.+Res.+Clin.+Haematol.&t
l+peritonitis+in+hepatic+inferior+vena+cava+disease%3A+A+hypothesis+to+explain+the+c
Pediatr.+Hematol.+Oncol.&title=International+Journal+of+Pediatric+Hematology%2FOncology

/lim OR [pubmed-not-medline]/lim))

thogens&volume=11&issue=3&spage=&epage=&aulast=Poveda-Urkixo&aufirst=Irati&auinit=
in.+J.+Sport+Med.&title=Clinical+Journal+of+Sport+Medicine&volume=32&issue=2&spage=
case+study&stitle=J.+Invest.+Med.&title=Journal+of+Investigative+Medicine&volume=70&iss
=J.+Clin.+Exp.+Hepatol.&title=Journal+of+Clinical+and+Experimental+Hepatology&volume=1
Hemophagocytic+Syndrome+in+Bone+Marrow&stitle=Clin.+Pathol.&title=Clinical+Pathology&
v.+Hematol.+Oncol.&title=Clinical+Advances+in+Hematology+and+Oncology&volume=20&iss
mekongi&stitle=IDCases&title=IDCases&volume=27&issue=&spage=&epage=&aulast=Burky
+Asthma+Clin.+Immunol.&title=Allergy%2C+Asthma+and+Clinical+Immunology&volume=18&i
ogens&volume=10&issue=12&spage=&epage=&aulast=Steiner&aufirst=Verena&auinit=V.&au
+Case+Reports&volume=2021&issue=12&spage=&epage=&aulast=Deleuze&aufirst=Catherin
+TX%2C+2019+and+2020.&stitle=Open+Forum+Infect.+Dis.&title=Open+Forum+Infectious+
ne+literature&stitle=Open+Forum+Infect.+Dis.&title=Open+Forum+Infectious+Diseases&vol
title=American+Journal+of+Gastroenterology&volume=116&issue=SUPPL&spage=S1488&ep
f+Medicine&volume=15&issue=3&spage=55&epage=56&aulast=Pestelli&aufirst=G.&auinit=(
title=Ital.+J.+Med.&title=Italian+Journal+of+Medicine&volume=15&issue=3&spage=179&epa
ct.+Dis.+Clin.+Pract.&title=Infectious+Diseases+in+Clinical+Practice&volume=29&issue=5&
and+sickle+cell+disease+treated+with+an+ex-vivo+t+cell+depleted+haploidentical+stem+c
&stitle=J.+Clin.+Diagn.+Res.&title=Journal+of+Clinical+and+Diagnostic+Research&volume=
diol.+Case+Rep.&title=Radiology+Case+Reports&volume=16&issue=1&spage=108&epage=1
ted+inflammation%2C+chronic+anemia%2C+and+altered+myelopoiesis&stitle=Infect.+Immun
title=IDCases&title=IDCases&volume=23&issue=&spage=&epage=&aulast=Tanveer&aufirst=
Rep.&title=Journal+of+Feline+Medicine+and+Surgery+Open+Reports&volume=7&issue=1&sp
wel+infarction.+A+systematic+review+of+the+most+recent+cases&stitle=Infez.+Med.&title
=26&issue=&spage=&epage=&aulast=Thakur&aufirst=Abhishek&auinit=A.&aufull=Thakur+A
ge=65&aulast=Fraia&aufirst=Anna+Sara&auinit=A.S.&aufull=Fraia+A.S.&coden=&isbn=&pag
ne+fibroid+disease&stitle=J.+Gen.+Intern.+Med.&title=Journal+of+General+Internal+Medicin
urnal+of+the+American+Society+of+Nephrology&volume=32&issue=&spage=139&epage=&a
&title=Journal+of+Investigative+Medicine&volume=70&issue=&spage=313&epage=&aulast=D
ogy&volume=115&issue=SUPPL&spage=S1735&epage=S1736&aulast=Dalal&aufirst=Kunal+
le=Am.+J.+Gastroenterol.&title=American+Journal+of+Gastroenterology&volume=115&issue=
si&volume=14&issue=3&spage=e111&epage=e116&aulast=Kara&aufirst=Tu%C4%9F%C3%A
ne+great+masquerader+melioidosis%3A+A+case+series&stitle=Indian+J.+Nucl.+Med.&title=
01%2C+G%29%2C+ibrutinib+%28I%29%2C+plus+venetoclax+%28VE%29+in+untreated+patie
cuk+Enfeksiyon+Derg.&title=Cocuk+Enfeksiyon+Dergisi&volume=14&issue=1&spage=e35&e
+study&stitle=Asian+Pac.+J.+Trop.+Med.&title=Asian+Pacific+Journal+of+Tropical+Medici
+Infect.+Dis.&title=Open+Forum+Infectious+Diseases&volume=7&issue=3&spage=&epage=

y+underappreciated+disease&stitle=IDCases&title=IDCases&volume=19&issue=&spage=&e
=360&aulast=Rafique&aufirst=Arshad&auinit=A.&aufull=Rafique+A.&coden=&isbn=&pages=3
-with+Overexpression+of+BCL1+Protein&stitle=Case+Rep.+Oncol.+Med.&title=Case+Repor
+A+report+of+three+cases&stitle=ecancermedicalscience&title=ecancermedicalscience&vol
dominal+variation+of+Lemierre+syndrome&stitle=Acta+Clin.+Belg.&title=Acta+Clinica+Belgi
olume=67&issue=SUPPL+1&spage=&epage=&aulast=Nunez&aufirst=Farranaz+Alvarez&auir
6&issue=4&spage=A623&epage=&aulast=Itani&aufirst=Wafic&auinit=W.&aufull=Itani+W.&c
+routine+dental+cleaning&stitle=Am.+J.+Gastroenterol.&title=American+Journal+of+Gastroe
nterology&volume=114&issue=&spage=S1057&epage=&aulast=Boland&aufirst=Katherine&a
oan+und+Vogel+GmbH&title=Infection&volume=47&issue=&spage=S24&epage=S25&aulast=
rnal+Medicine&volume=34&issue=2&spage=S637&epage=S638&aulast=Kovar&aufirst=Nora
ancreatectomy+with+splenectomy&stitle=Ital.+J.+Med.&title=Italian+Journal+of+Medicine&
Critical+Care+Medicine&volume=199&issue=9&spage=&epage=&aulast=Izakovich&aufirst=T
immunosuppressive+therapy%3A+Diagnosis+and+management&stitle=Indian+J.+Transplant.&
+Enfeksiyon+Dergisi&volume=13&issue=1&spage=e28&epage=e31&aulast=%C3%96zbay&au
+Rep.&title=Clinical+Case+Reports&volume=7&issue=3&spage=533&epage=536&aulast=P
n+syndrome+be+considered%3F&stitle=Iran.+J.+Pediatr.&title=Iranian+Journal+of+Pediatrics
t.+Med.&title=Journal+of+Investigative+Medicine&volume=67&issue=2&spage=452&epage=
3&issue=&spage=&epage=&aulast=Ramnarain&aufirst=Jaineel&auinit=J.&aufull=Ramnarain+
stitial+lung+disease&stitle=LymphoSign+J.&title=LymphoSign+Journal&volume=5&issue=4&s
+remission+after+stem+cell+transplantation&stitle=ecancermedicalscience&title=ecancerm
gical+findings+with+its+haematology+analyser-based+expression&stitle=J.+Hematopatholog
+Clin.+Oncol.&title=Molecular+and+Clinical+Oncology&volume=8&issue=5&spage=637&epa
=12&issue=2&spage=118&epage=119&aulast=Valiani&aufirst=V.&auinit=V.&aufull=Valiani+V
stitle=Front.+Microbiol.&title=Frontiers+in+Microbiology&volume=9&issue=JAN&spage=&ep
.+Case+report+and+review+of+the+literature&stitle=Int.+J.+Surg.+Case+Rep.&title=Interna
me=13&issue=4&spage=&epage=&aulast=Zhmurouski&aufirst=Dzmitry&auinit=D.&aufull=Zh
&issue=4&spage=&epage=&aulast=Pascoe&aufirst=Jennifer&auinit=J.&aufull=Pascoe+J.&co
4&spage=&epage=&aulast=Khanin&aufirst=Yuriy&auinit=Y.&aufull=Khanin+Y.&coden=&isbn
ase+Rep.&title=Journal+of+Investigative+Medicine+High+Impact+Case+Reports&volume=5&
J.+Antimicrob.+Agents&title=International+Journal+of+Antimicrobial+Agents&volume=50&iss
+Journal+of+Gastroenterology&volume=112&issue=&spage=S1248&epage=&aulast=Herlihy&
=Am.+J.+Gastroenterol.&title=American+Journal+of+Gastroenterology&volume=112&issue=&
adilino+syndrome&stitle=Am.+J.+Gastroenterol.&title=American+Journal+of+Gastroenterolog
case+report&stitle=Am.+J.+Gastroenterol.&title=American+Journal+of+Gastroenterology&vo
en+Forum+Infectious+Diseases&volume=4&issue=&spage=S120&epage=&aulast=Ayed&auf
en+Forum+Infectious+Diseases&volume=4&issue=&spage=S120&epage=&aulast=Ayed&aufi
tle=Hepatology+International&volume=11&issue=1&spage=S1092&epage=&aulast=Hlaing&au
l+of+Primary+Care&volume=9&issue=5&spage=77&epage=79&aulast=Basetti&aufirst=S.&a
=128&issue=22&spage=&epage=&aulast=Qiu&aufirst=Hongxia&auinit=H.&aufull=Qiu+H.&co

nd+Paediatric+Oncology&volume=37&issue=4&spage=311&epage=313&aulast=Abdulla&aufi
t+Med.+J.&title=Kuwait+Medical+Journal&volume=48&issue=3&spage=241&epage=244&aul
=S524&epage=S525&aulast=Seth&aufirst=Aradhna&auinit=A.&aufull=Seth+A.&coden=&isb
&volume=31&issue=2&spage=S561&epage=S562&aulast=Hammer&aufirst=Rachel&auinit=
ledicine&volume=64&issue=4&spage=938&epage=939&aulast=Al-Khazraji&aufirst=A.&auini
le=Case+Reports+in+Medicine&volume=2016&issue=&spage=&epage=&aulast=Faraone&a
110%CE%B4+PI%283%29+kinase+catalytic+subunit%3A+A+report+of+two+cases&stitle=A
spir.+Crit.+Care+Med.&title=American+Journal+of+Respiratory+and+Critical+Care+Medicin
Internal+Medicine&volume=30&issue=&spage=S450&epage=S451&aulast=Xu&aufirst=Jin&a
on&volume=95&issue=&spage=8A&epage=&aulast=Melo-Uribe&aufirst=Mario&auinit=M.&a
+Southern+Israel%3A+A+7-year+retrospective+study+%282005-2011%29&stitle=Vector+Bo
-infected+patients+in+resource-limited+settings&stitle=Open+Forum+Infect.+Dis.&title=O
=IDCases&volume=2&issue=3&spage=72&epage=76&aulast=Akhrass&aufirst=Fadi+Al&auin
ced+end-organ+failure&stitle=J.+Tehran.+Uni.+Heart.+Cent.&title=Journal+of+Tehran+Univ
ulast=Zarzour&aufirst=Ahmad&auinit=A.&aufull=Zarzour+A.&coden=&isbn=&pages=-&date
e=Am.+J.+Respir.+Crit.+Care+Med.&title=American+Journal+of+Respiratory+and+Critical+
-Infect.+Dis.&title=Journal+of+Pediatric+Infectious+Diseases&volume=9&issue=1&spage=1
=4&spage=70&epage=71&aulast=Gill&aufirst=Veenu&auinit=V.&aufull=Gill+V.&coden=&isb
h+scrotal+swelling&stitle=Int.+J.+Surg.+Case+Rep.&title=International+Journal+of+Surgery
le=Crit.+Care+Med.&title=Critical+Care+Medicine&volume=41&issue=12&spage=A311&epa
hma+Immunol.&title=Annals+of+Allergy%2C+Asthma+and+Immunology&volume=111&issue=
ale&aufirst=David+C.&auinit=D.C.&aufull=Dale+D.C.&coden=&isbn=&pages=-&date=2013&
l.&title=American+Journal+of+Gastroenterology&volume=108&issue=&spage=S334&epage=
Med.&title=American+Journal+of+Respiratory+and+Critical+Care+Medicine&volume=187&is
erology&volume=26&issue=2&spage=170&epage=172&aulast=Taya&aufirst=Yeng+Kwang&a
+Infectious+Diseases&volume=4&issue=1&spage=&epage=&aulast=Schumacher&aufirst=Ri
thern+Vietnam&stitle=AIDS+Res.+Ther.&title=AIDS+Research+and+Therapy&volume=9&iss
chives+of+Disease+in+Childhood&volume=97&issue=&spage=A10&epage=&aulast=Bentur&
d+lower+day+100+survival&stitle=Bone+Marrow+Transplant.&title=Bone+Marrow+Transpla
Journal&volume=29&issue=3&spage=331&epage=333&aulast=Ye%C5%9Filkaya&aufirst=Ay%
=&aulast=Gupta&aufirst=Nishant&auinit=N.&aufull=Gupta+N.&coden=&isbn=&pages=-&da
nal+trisomy+8+mosaicism&stitle=Bone+Marrow+Transplant.&title=Bone+Marrow+Transpla
+Medicine&volume=38&issue=&spage=A283&epage=&aulast=Siddiqui&aufirst=Asma&auinit
atologica&volume=95&issue=&spage=524&epage=&aulast=Iannitto&aufirst=E.&auinit=E.&a
+connective+tissue+disease+%28CTD%29&stitle=J.+Clin.+Rheumatol.&title=Journal+of+Cli
ssue=&spage=155&epage=&aulast=Krishnamurthy&aufirst=Mahesh&auinit=M.&aufull=Krish
lume=58&issue=2&spage=412&epage=&aulast=Macariola&aufirst=D.&auinit=D.&aufull=Ma
ardiol.&title=Journal+fur+Kardiologie&volume=16&issue=9-10&spage=364&epage=367&aul
me=18&issue=3&spage=80&epage=83&aulast=Haresh&aufirst=Tulsidas&auinit=T.&aufull=
ia&stitle=Infect.+Dis.+Clin.+Pract.&title=Infectious+Diseases+in+Clinical+Practice&volum

t&stitle=J.+Med.+Case+Rep.&title=Journal+of+Medical+Case+Reports&volume=2&issue=&s
report&stitle=J.+Med.+Case+Rep.&title=Journal+of+Medical+Case+Reports&volume=2&issu
e=Enfermedades+Infecciosas+y+Microbiologia+Clinica&volume=26&issue=SUPPL.+5&spage
&title=Enfermedades+Infecciosas+y+Microbiologia+Clinica&volume=26&issue=SUPPL.+5&s
olume=9&issue=1&spage=17&epage=22&aulast=Narain&aufirst=Nigam+P.&auinit=N.P.&aufu
rdiszip.&title=Chirurgische+Gastroenterologie+Interdisziplinar&volume=22&issue=3&spage=2
me=19&issue=3&spage=185&epage=187&aulast=Haasper&aufirst=Cad&auinit=C.&aufull=H
itle=Best+Practice+and+Research+in+Clinical+Haematology&volume=16&issue=1&spage=1
ause+of+infection+in+high+protein+ascites&stitle=Hepatol.+Res.&title=Hepatology+Resear
&volume=6&issue=2&spage=131&epage=145&aulast=Gazda&aufirst=H.&auinit=H.&aufull=G

I.&aufull=Poveda-Urkixo+I.&coden=&isbn=&pages=-&date=2022&auinit1=I&auinitm=
e192&epage=e193&aulast=Davis&aufirst=Cameron&auinit=C.&aufull=Davis+C.&coden=&isb
sue=2&spage=495&epage=&aulast=Falco&aufirst=A.&auinit=A.&aufull=Falco+A.&coden=&is
2&issue=1&spage=222&epage=224&aulast=Niyas&aufirst=Vettakkara+K.M.&auinit=V.K.M.&
&volume=15&issue=&spage=&epage=&aulast=Wan+Mohd+Zohdi&aufirst=Wan+Awatif&auini
sue=1&spage=7&epage=24&aulast=Chen&aufirst=Yi-Bin&auinit=Y.-B.&aufull=Chen+Y.-B.&c
&aufirst=Matthew&auinit=M.&aufull=Burky+M.&coden=&isbn=&pages=-&date=2022&auinit
ssue=SUPPL+1&spage=&epage=&aulast=Ayoub&aufirst=Abdul+Rahman&auinit=A.R.&aufull
ufull=Steiner+V.&coden=&isbn=&pages=-&date=2021&auinit1=V&auinitm=
e&auinit=C.&aufull=Deleuze+C.&coden=&isbn=&pages=-&date=2021&auinit1=C&auinitm=
Diseases&volume=8&issue=SUPPL+1&spage=S449&epage=S450&aulast=Tabarani&aufirst=
ume=8&issue=10&spage=&epage=&aulast=Gopalsamy&aufirst=Srinivasa+Nithin&auinit=S.N
age=S1489&aulast=Patel&aufirst=Pranav&auinit=P.&aufull=Patel+P.&coden=&isbn=&pages
G.&aufull=Pestelli+G.&coden=&isbn=&pages=55-56&date=2021&auinit1=G&auinitm=
ge=183&aulast=Magnani&aufirst=Ottavia&auinit=O.&aufull=Magnani+O.&coden=IJMEE&isb
&spage=E317&epage=E320&aulast=Aggarwal&aufirst=Abhimanyu&auinit=A.&aufull=Aggarwa
ell+transplant&stitle=Pediatr.+Blood+Cancer&title=Pediatric+Blood+and+Cancer&volume=6
15&issue=4&spage=SR04&epage=SR06&aulast=Marwah&aufirst=Ashish&auinit=A.&aufull=I
12&aulast=Chikamori&aufirst=Fumio&auinit=F.&aufull=Chikamori+F.&coden=&isbn=&pages
.&title=Infection+and+Immunity&volume=89&issue=1&spage=&epage=&aulast=Loomis&aufi
=Farah&auinit=F.&aufull=Tanveer+F.&coden=&isbn=&pages=-&date=2021&auinit1=F&auini
age=&epage=&aulast=Wood&aufirst=James&auinit=J.&aufull=Wood+J.&coden=&isbn=&pag
=Infezioni+in+Medicina&volume=29&issue=3&spage=469&epage=474&aulast=Saad&aufirst=
.&coden=&isbn=&pages=-&date=2021&auinit1=A&auinitm=
es=49-65&date=2021&auinit1=A&auinitm=S
e&volume=36&issue=SUPPL+1&spage=S231&epage=&aulast=Ng+Sueng&aufirst=Luis+F.&
ulast=Kanipakam&aufirst=Reddappa+Venkata+Sai+Rakesh&auinit=R.V.S.R.&aufull=Kanipaka
D%27Assumpcao&aufirst=C.&auinit=C.&aufull=D%27Assumpcao+C.&coden=&isbn=&pages=
K.&auinit=K.K.&aufull=Dalal+K.K.&coden=&isbn=&pages=S1735-S1736&date=2020&auinit1
=SUPPL&spage=S1299&epage=&aulast=Uhlenhopp&aufirst=Dustin&auinit=D.&aufull=Uhlenh
7e+Tural&auinit=T.T.&aufull=Kara+T.T.&coden=&isbn=&pages=e111-e116&date=2020&auir
Indian+Journal+of+Nuclear+Medicine&volume=35&issue=3&spage=222&epage=225&aulast=
nts+with+CLL+with+17p+deletion+%2F+TP53+mutation&stitle=HemaSphere&title=HemaS
page=e37&aulast=Ya%C5%9Far+Durmu%C5%9F&aufirst=Sevgi&auinit=S.&aufull=Ya%C5%9
ne&volume=13&issue=3&spage=115&epage=122&aulast=Thabit&aufirst=Alif&auinit=A.&aufu
=&aulast=Rieg&aufirst=Siegbert&auinit=S.&aufull=Rieg+S.&coden=&isbn=&pages=-&date=

page=&aulast=Schmalzle&aufirst=S.A.&auinit=S.A.&aufull=Schmalzle+S.A.&coden=&isbn=&
56-360&date=2020&auinit1=A&auinitm=
ts+in+Oncological+Medicine&volume=2020&issue=&spage=&epage=&aulast=Dabrowski&au
ume=14&issue=&spage=&epage=&aulast=Burns&aufirst=Ethan+A.&auinit=E.A.&aufull=Burr
ca&volume=74&issue=&spage=23&epage=&aulast=Collard&aufirst=Charl%C3%A8ne&auinit=
nit=F.A.&aufull=Nunez+F.A.&coden=&isbn=&pages=-&date=2020&auinit1=F&auinitm=A
oden=&isbn=&pages=A623-&date=2019&auinit1=W&auinitm=
nterology&volume=114&issue=&spage=S1273&epage=&aulast=Hanna&aufirst=Angy&auinit=
uinit=K.&aufull=Boland+K.&coden=&isbn=&pages=S1057-&date=2019&auinit1=K&auinitm=
=Guerra&aufirst=J.&auinit=J.&aufull=Guerra+J.&coden=&isbn=&pages=S24-S25&date=2019&
&auinit=N.&aufull=Kovar+N.&coden=&isbn=&pages=S637-S638&date=2019&auinit1=N&auir
olume=13&issue=&spage=102&epage=&aulast=Pulizzi&aufirst=N.&auinit=N.&aufull=Pulizzi
.&auinit=T.&aufull=Izakovich+T.&coden=&isbn=&pages=-&date=2019&auinit1=T&auinitm=
&title=Indian+Journal+of+Transplantation&volume=13&issue=2&spage=141&epage=144&aula
ufirst=Deniz&auinit=D.&aufull=%C3%96zbay+D.&coden=&isbn=&pages=e28-e31&date=2019
uar&aufirst=Neha&auinit=N.&aufull=Puar+N.&coden=&isbn=&pages=533-536&date=2019&a
s&volume=29&issue=1&spage=&epage=&aulast=Mousavi&aufirst=Mahdieh+Sadat&auinit=M
&aulast=Bouchillon&aufirst=J.M.&auinit=J.M.&aufull=Bouchillon+J.M.&coden=&isbn=&pages
J.&coden=&isbn=&pages=-&date=2019&auinit1=J&auinitm=
spage=146&epage=148&aulast=Choea&aufirst=Noreen&auinit=N.&aufull=Choea+N.&coden=
edicalscience&volume=12&issue=&spage=&epage=&aulast=Sun&aufirst=Kai&auinit=K.&auf
y&title=Journal+of+Hematopathology&volume=11&issue=3&spage=87&epage=92&aulast=Bh
age=639&aulast=Siddiqi&aufirst=Ahsan&auinit=A.&aufull=Siddiqi+A.&coden=&isbn=&pages=
/.&coden=&isbn=&pages=118-119&date=2018&auinit1=V&auinitm=
age=&aulast=Djokic&aufirst=Vitomir&auinit=V.&aufull=Djokic+V.&coden=&isbn=&pages=-&
ational+Journal+of+Surgery+Case+Reports&volume=43&issue=&spage=36&epage=40&aulas
murouski+D.&coden=&isbn=&pages=-&date=2018&auinit1=D&auinitm=
den=&isbn=&pages=-&date=2018&auinit1=J&auinitm=
=&pages=-&date=2018&auinit1=Y&auinitm=
issue=4&spage=&epage=&aulast=Ambesh&aufirst=Paurush&auinit=P.&aufull=Ambesh+P.&
ue=&spage=S164&epage=S165&aulast=Kow&aufirst=Jia-Yi&auinit=J.-Y.&aufull=Kow+J.-Y.&
aufirst=John+D.&auinit=J.D.&aufull=Herlihy+J.D.&coden=&isbn=&pages=S1248-&date=2017
spage=S1259&epage=&aulast=Pribish&aufirst=Abby&auinit=A.&aufull=Pribish+A.&coden=&i
y&volume=112&issue=&spage=S1288&epage=&aulast=Chandrakumaran&aufirst=Anchalia&a
lume=112&issue=&spage=S1215&epage=S1216&aulast=Pointer&aufirst=Lauren&auinit=L.&a
irst=Houda+Ben&auinit=H.B.&aufull=Ayed+H.B.&coden=&isbn=&pages=S120-&date=2017&
rst=Houda+Ben&auinit=H.B.&aufull=Ayed+H.B.&coden=&isbn=&pages=S120-&date=2017&a
ufirst=Tin+Tin&auinit=T.T.&aufull=Hlaing+T.T.&coden=&isbn=&pages=S1092-&date=2017&
uinit=S.&aufull=Basetti+S.&coden=&isbn=&pages=77-79&date=2017&auinit1=S&auinitm=
den=&isbn=&pages=-&date=2016&auinit1=H&auinitm=

rst=Mansoor&auinit=M.&aufull=Abdulla+M.&coden=&isbn=&pages=311-313&date=2016&au
ast=Sobaih&aufirst=Badr+Hasan&auinit=B.H.&aufull=Sobaih+B.H.&coden=&isbn=&pages=2
n=&pages=S524-S525&date=2016&auinit1=A&auinitm=
R.&aufull=Hammer+R.&coden=&isbn=&pages=S561-S562&date=2016&auinit1=R&auinitm=
t=A.&aufull=Al-Khazraji+A.&coden=&isbn=&pages=938-939&date=2016&auinit1=A&auinitm=
ufirst=Antonio&auinit=A.&aufull=Faraone+A.&coden=&isbn=&pages=-&date=2016&auinit1=A
n.+Allergy+Asthma+Immunol.&title=Annals+of+Allergy%2C+Asthma+and+Immunology&volur
ne&volume=191&issue=&spage=&epage=&aulast=Pershwitz&aufirst=G.E.&auinit=G.E.&auful
uinit=J.&aufull=Xu+J.&coden=&isbn=&pages=S450-S451&date=2015&auinit1=J&auinitm=
ufull=Melo-Uribe+M.&coden=&isbn=&pages=8A-&date=2015&auinit1=M&auinitm=
rne+Zoonotic+Dis.&title=Vector-Borne+and+Zoonotic+Diseases&volume=15&issue=3&spage
pen+Forum+Infectious+Diseases&volume=2&issue=1&spage=&epage=&aulast=Murphy&aufi
it=F.A.&aufull=Akhrass+F.A.&coden=&isbn=&pages=72-76&date=2015&auinit1=F&auinitm=
ersity+Heart+Center&volume=10&issue=4&spage=201&epage=204&aulast=Demirozu&aufirs
=2014&auinit1=A&auinitm=
Care+Medicine&volume=189&issue=&spage=&epage=&aulast=Sutton&aufirst=E.&auinit=E.&
9&epage=22&aulast=Ikhlas&aufirst=Ahmad&auinit=A.&aufull=Ikhlas+A.&coden=&isbn=&pag
n=&pages=70-71&date=2014&auinit1=V&auinitm=
+Case+Reports&volume=5&issue=12&spage=951&epage=953&aulast=McPhillips&aufirst=Se
ge=&aulast=Sharma&aufirst=Ramakant&auinit=R.&aufull=Sharma+R.&coden=&isbn=&pages
5&spage=A65&epage=&aulast=Thakor&aufirst=A.&auinit=A.&aufull=Thakor+A.&coden=&isb
auinit1=D&auinitm=C
&aulast=Pillai&aufirst=Ajish&auinit=A.&aufull=Pillai+A.&coden=&isbn=&pages=S334-&date
sue=&spage=&epage=&aulast=Smith&aufirst=J.P.&auinit=J.P.&aufull=Smith+J.P.&coden=&i
uinit=Y.K.&aufull=Taya+Y.K.&coden=&isbn=&pages=170-172&date=2013&auinit1=Y&auinitm
chard-Fabian&auinit=R.-F.&aufull=Schumacher+R.-F.&coden=&isbn=&pages=-&date=2012&
ue=&spage=&epage=&aulast=Larsson&aufirst=Mattias&auinit=M.&aufull=Larsson+M.&cod
aufirst=H.&auinit=H.&aufull=Bentur+H.&coden=&isbn=&pages=A10-&date=2012&auinit1=H
ntation&volume=47&issue=&spage=S198&epage=S199&aulast=Kato&aufirst=A.&auinit=A.&
6C5%9Feg%C3%BCl&auinit=A.&aufull=Ye%C5%9Filkaya+A.&coden=&isbn=&pages=331-333
te=2011&auinit1=N&auinitm=
ntation&volume=46&issue=&spage=S148&epage=S149&aulast=Elhasid&aufirst=R.&auinit=R
=A.&aufull=Siddiqui+A.&coden=&isbn=&pages=A283-&date=2010&auinit1=A&auinitm=
ufull=Iannitto+E.&coden=&isbn=&pages=524-&date=2010&auinit1=E&auinitm=
nical+Rheumatology&volume=16&issue=&spage=S95&epage=&aulast=Navarro&aufirst=I.Y.&
namurthy+M.&coden=&isbn=&pages=155-&date=2010&auinit1=M&auinitm=
cariola+D.&coden=&isbn=&pages=412-&date=2010&auinit1=D&auinitm=
ast=Mulac&aufirst=Karl&auinit=K.&aufull=Mulac+K.&coden=JKARF&isbn=&pages=364-367
Haresh+T.&coden=&isbn=&pages=80-83&date=2009&auinit1=T&auinitm=
e=17&issue=6&spage=407&epage=408&aulast=Oever&aufirst=Jaap+Ten&auinit=J.T.&aufull=

spage=&epage=&aulast=Tommasi&aufirst=Chiara&auinit=C.&aufull=Tommasi+C.&coden=&i
ue=&spage=&epage=&aulast=Mohiyiddeen&aufirst=Gadha&auinit=G.&aufull=Mohiyiddeen+G
=6&epage=11&aulast=Iribarren&aufirst=Jos%C3%A9+Antonio&auinit=J.A.&aufull=Iribarren+
page=54&epage=61&aulast=Cabezos+Ot%C3%B3n&aufirst=Juan&auinit=J.&aufull=Cabezos+
ull=Narain+N.P.&coden=IJPPG&isbn=&pages=17-22&date=2007&auinit1=N&auinitm=P
01&epage=203&aulast=Chen&aufirst=Shih-Yi&auinit=S.-Y.&aufull=Chen+S.-Y.&coden=CGIH
aasper+C.&coden=INPDE&isbn=&pages=185-187&date=2004&auinit1=C&auinitm=
01&epage=116&aulast=Goodman&aufirst=Grant+R.&auinit=G.R.&aufull=Goodman+G.R.&cod
ch&volume=24&issue=1&spage=42&epage=49&aulast=Shrestha&aufirst=Santosh+Man&auir
azda+H.&coden=IPHOE&isbn=&pages=131-145&date=1999&auinit1=H&auinitm=

ɔn=&pages=e192-e193&date=2022&auinit1=C&auinitm=
sbn=&pages=495-&date=2022&auinit1=A&auinitm=
&aufull=Niyas+V.K.M.&coden=&isbn=&pages=222-224&date=2022&auinit1=V&auinitm=K.M.
t=W.A.&aufull=Wan+Mohd+Zohdi+W.A.&coden=&isbn=&pages=-&date=2022&auinit1=W&au
oden=&isbn=&pages=7-24&date=2022&auinit1=Y&auinitm=-B
1=M&auinitm=
=Ayoub+A.R.&coden=&isbn=&pages=-&date=2022&auinit1=A&auinitm=R

=Christy&auinit=C.&aufull=Tabarani+C.&coden=&isbn=&pages=S449-S450&date=2021&auin
.&aufull=Gopalsamy+S.N.&coden=&isbn=&pages=-&date=2021&auinit1=S&auinitm=N
=S1488-S1489&date=2021&auinit1=P&auinitm=

n=&pages=179-183&date=2021&auinit1=O&auinitm=
l+A.&coden=IDCPE&isbn=&pages=E317-E320&date=2021&auinit1=A&auinitm=
8&issue=SUPPL+3&spage=S190&epage=S191&aulast=Silbert&aufirst=Sara&auinit=S.&aufu
Marwah+A.&coden=&isbn=&pages=SR04-SR06&date=2021&auinit1=A&auinitm=
=108-112&date=2021&auinit1=F&auinitm=
rst=Wendy+P.&auinit=W.P.&aufull=Loomis+W.P.&coden=INFIB&isbn=&pages=-&date=2021
tm=
es=-&date=2021&auinit1=J&auinitm=
=Mariam+Ahmed&auinit=M.A.&aufull=Saad+M.A.&coden=INMEF&isbn=&pages=469-474&da

auinit=L.F.&aufull=Ng+Sueng+L.F.&coden=&isbn=&pages=S231-&date=2021&auinit1=L&au
am+R.V.S.R.&coden=&isbn=&pages=139-&date=2021&auinit1=R&auinitm=V.S.R.
313-&date=2021&auinit1=C&auinitm=
=K&auinitm=K
nopp+D.&coden=&isbn=&pages=S1299-&date=2020&auinit1=D&auinitm=
nit1=T&auinitm=T
Kulkarni&aufirst=Pramukh&auinit=P.&aufull=Kulkarni+P.&coden=&isbn=&pages=222-225&c
ohere&volume=4&issue=&spage=32&epage=&aulast=Huber&aufirst=H.&auinit=H.&aufull=Hu
9Far+Durmu%C5%9F+S.&coden=&isbn=&pages=e35-e37&date=2020&auinit1=S&auinitm=
ıll=Thabit+A.&coden=&isbn=&pages=115-122&date=2020&auinit1=A&auinitm=
2020&auinit1=S&auinitm=

pages=-&date=2020&auinit1=S&auinitm=A

ıfirst=Dominik&auinit=D.&aufull=Dabrowski+D.&coden=&isbn=&pages=-&date=2020&auinit
ıs+E.A.&coden=&isbn=&pages=-&date=2020&auinit1=E&auinitm=A
=C.&aufull=Collard+C.&coden=&isbn=&pages=23-&date=2020&auinit1=C&auinitm=

A.&aufull=Hanna+A.&coden=&isbn=&pages=S1273-&date=2019&auinit1=A&auinitm=

ıauinit1=J&auinitm=
ıitm=
+N.&coden=&isbn=&pages=102-&date=2019&auinit1=N&auinitm=

ast=Anupama&aufirst=Sneha+Haridas&auinit=S.H.&aufull=Anupama+S.H.&coden=&isbn=&pa
&auinit1=D&auinitm=
uinit1=N&auinitm=
/I.S.&aufull=Mousavi+M.S.&coden=&isbn=&pages=-&date=2019&auinit1=M&auinitm=S
=452-&date=2019&auinit1=J&auinitm=M

&isbn=&pages=146-148&date=2018&auinit1=N&auinitm=
ull=Sun+K.&coden=&isbn=&pages=-&date=2018&auinit1=K&auinitm=
ıola&aufirst=Rajesh+Kumar&auinit=R.K.&aufull=Bhola+R.K.&coden=&isbn=&pages=87-92&d
=637-639&date=2018&auinit1=A&auinitm=

ıdate=2018&auinit1=V&auinitm=
t=Rizzuto&aufirst=Antonia&auinit=A.&aufull=Rizzuto+A.&coden=&isbn=&pages=36-40&date=

coden=&isbn=&pages=-&date=2017&auinit1=P&auinitm=
ıcoden=&isbn=&pages=S164-S165&date=2017&auinit1=J&auinitm=-Y
&auinit1=J&auinitm=D
sbn=&pages=S1259-&date=2017&auinit1=A&auinitm=
ıuinit=A.&aufull=Chandrakumaran+A.&coden=&isbn=&pages=S1288-&date=2017&auinit1=A&
aufull=Pointer+L.&coden=&isbn=&pages=S1215-S1216&date=2017&auinit1=L&auinitm=
auinit1=H&auinitm=B
ıuinit1=H&auinitm=B
auinit1=T&auinitm=T

init1=M&auinitm=
41-244&date=2016&auinit1=B&auinitm=H

=
\&auinitm=
ne=115&issue=5&spage=A99&epage=A100&aulast=Feuille&aufirst=E.J.&auinit=E.J.&aufull=F
l=Pershwitz+G.E.&coden=&isbn=&pages=-&date=2015&auinit1=G&auinitm=E

e=195&epage=201&aulast=Fruchtman&aufirst=Yariv&auinit=Y.&aufull=Fruchtman+Y.&coden
rst=Richard+A.&auinit=R.A.&aufull=Murphy+R.A.&coden=&isbn=&pages=-&date=2015&auin
A
t=Zumrut+Tuba&auinit=Z.T.&aufull=Demirozu+Z.T.&coden=&isbn=&pages=201-204&date=2

aufull=Sutton+E.&coden=&isbn=&pages=-&date=2014&auinit1=E&auinitm=
es=19-22&date=2014&auinit1=A&auinitm=

ean&auinit=S.&aufull=McPhillips+S.&coden=&isbn=&pages=951-953&date=2014&auinit1=S&
=A311-&date=2013&auinit1=R&auinitm=
n=&pages=A65-&date=2013&auinit1=A&auinitm=

=2013&auinit1=A&auinitm=
sbn=&pages=-&date=2013&auinit1=J&auinitm=P
n=K
auinit1=R&auinitm=-F
en=&isbn=&pages=-&date=2012&auinit1=M&auinitm=
&auinitm=
aufull=Kato+A.&coden=&isbn=&pages=S198-S199&date=2012&auinit1=A&auinitm=
&date=2012&auinit1=A&auinitm=

.&aufull=Elhasid+R.&coden=&isbn=&pages=S148-S149&date=2011&auinit1=R&auinitm=

auinit=I.Y.&aufull=Navarro+I.Y.&coden=&isbn=&pages=S95-&date=2010&auinit1=I&auinitm=

&date=2009&auinit1=K&auinitm=

=Oever+J.T.&coden=IDCPE&isbn=&pages=407-408&date=2009&auinit1=J&auinitm=T

isbn=&pages=-&date=2008&auinit1=C&auinitm=
.&coden=&isbn=&pages=-&date=2008&auinit1=G&auinitm=
J.A.&coden=EIMCE&isbn=&pages=6-11&date=2008&auinit1=J&auinitm=A
+Ot%C3%B3n+J.&coden=EIMCE&isbn=&pages=54-61&date=2008&auinit1=J&auinitm=

HA&isbn=&pages=201-203&date=2006&auinit1=S&auinitm=-Y

en=BPRCA&isbn=&pages=101-116&date=2003&auinit1=G&auinitm=R
nit=S.M.&aufull=Shrestha+S.M.&coden=HPRSF&isbn=&pages=42-49&date=2002&auinit1=S

ıinitm=A

it1=C&auinitm=

ll=Silbert+S.&coden=&isbn=&pages=S190-S191&date=2021&auinit1=S&auinitm=

&auinit1=W&auinitm=P

te=2021&auinit1=M&auinitm=A

ıinitm=F

date=2020&auinit1=P&auinitm=
ıber+H.&coden=&isbn=&pages=32-&date=2020&auinit1=H&auinitm=

1=D&auinitm=

ages=141-144&date=2019&auinit1=S&auinitm=H

late=2018&auinit1=R&auinitm=K

=2018&auinit1=A&auinitm=

&auinitm=

Feuille+E.J.&coden=&isbn=&pages=A99-A100&date=2015&auinit1=E&auinitm=J

=VZDEB&isbn=&pages=195-201&date=2015&auinit1=Y&auinitm=
it1=R&auinitm=A

2015&auinit1=Z&auinitm=T

&auinitm=

=Y

&auinitm=M
