# Supplementary material for: First case report of splenomegaly with splenic infarction due to aortic graft infection
Source: BMC Cardiovasc Disord. 2023 May 5;23:237. doi: 10.1186/s12872-023-03259-y (PMC10161471; doi:10.1186/s12872-023-03259-y)
Supplement: Supplementary file 1 — Additional file 1. [file 12872_2023_3259_MOESM1_ESM.zip › Splenomegaly_Ichushi_ESM.pdf]

| ID | Language | Authors | Title | Journal | Year |
|---|---|---|---|---|---|
| W317170072<I | 日本語 | 金 允泰, 石川 利 | 脾腫、脾梗塞を合併したEnter | 日本内科学会関東 | 2022 |
| W210430006<I | 日本語 | 高島 明美, 長谷 | サイトメガロウイルス感染症に | 日本病院総合診療 | 2022 |
| 2022080414 | 日本語 | 野村 綾 | LETTERS TO THE EDITORに | 日本病院総合診療 | 2021 |
| 2022080413 | 日本語 | 五十野 博基 | 本誌第17巻4号「脾腫を伴い | 日本病院総合診療 | 2021 |
| 2021014506 | 日本語 | 宮崎 麻衣, 末永 | HIV患者に発症した非外傷性 | 日本臨床外科学会 | 2020 |
| 2020302788 | 英語 | Hisamura Masak | Splenic mass of uncertain eti | Acute Medicine & | 2020 |
| 2020255065 | 英語 | Komatsu Toshir | Percutaneous Transcatheter | 信州医学雑誌 | 2019 |
| 2020211809 | 日本語 | 澤田 良子 | 検査説明Q&A 血小板輸血後 | 臨床検査 | 2020 |
| 2020203923 | 英語 | Fujiki Toshihiro, | Long-lasting low NK cell activ | Pediatrics Internat | 2019 |
| 2020178775 | 日本語 | 伊藤 由作, 塩田 | 一過性骨髄異常増殖症と鑑別 | 日本小児血液・が | 2019 |
| 2020176354 | 日本語 | 前田 隆求, 三田 | 大動脈術後の血球貪食性リン | 日本集中治療医学 | 2019 |
| 2020105305 | 日本語 | 山下 耕生, 田原 | 脾梗塞を合併したEBウイルス | 日本消化器病学会 | 2019 |
| 2020077309 | 英語 | Kusunoki Ryusa | Intravascular Large B-cell Ly | Internal Medicine | 2019 |
| 2020074196 | 英語 | Nishida Takashi, | Typhoid fever complicated by | The Kitasato Medi | 2018 |
| 2020046723 | 日本語 | 近藤 健, 塚本 知 | キーフレーズで読み解く 外来 | 日本医事新報 | 2019 |
| 2019267563 | 英語 | Kaneko Hiroto, | An adult-onset case of chror | Journal of Infectio | 2018 |
| 2019232015 | 日本語 | 植田 裕子, 牧田 | 成人発症の慢性活動性EBウ | 岡山医療センター | 2019 |
| 2019225880 | 日本語 | 有松 朋之, 宮本 | MYD88遺伝子変異の検出に | 日本農村医学会雑 | 2019 |
| 2019171052 | 英語 | Hajsadeghi Shok | Concurrent diagnosis of infec | Journal of Cardiolo | 2018 |
| 2019103266 | 英語 | Valencia Damiar | Leuconostoc species endoca | Journal of Cardiolo | 2018 |
| 2019102985 | 日本語 | 猪熊 孝実, 泉野 | 入院中に伝染性単核球症が | 日本腹部救急医学 | 2018 |
| 2019054015 | 英語 | Kusama Yoshiki, | A pediatric case of imported c | Journal of General | 2017 |
| 2018222999 | 日本語 | 小田 侑希, 相澤 | 不明熱の鑑別診断に肝生検 | 奈良県西和医療セ | 2018 |
| 2018187574 | 日本語 | 芦谷 啓吾, 山岡 | EBV感染による伝染性単核球 | 日本内科学会雑誌 | 2018 |
| 2017204049 | 日本語 | 廣川 哲太郎, 杉 | 病理解剖が有用であった、意 | 新潟県厚生連医誌 | 2017 |
| 2017129273 | 日本語 | 山田 尚友, 山田 | 移植医療における輸血関連検 | 日本検査血液学会 | 2016 |
| 2016410200 | 日本語 | 竹迫 弥生, 有岡 | 【腹痛を診る-非専門医に求め | 内科 | 2016 |
| 2016310130 | 英語 | Fukuda Yutaka, | Pulmonary arterial hypertensi | Pediatrics Internat | 2015 |
| 2016222103 | 日本語 | 横田 俊平 | 話題の疾患と治療 マクロファ | 感染・炎症・免疫 | 2016 |
| 2016211067 | 日本語 | 松木 充, 渡口 | 【不明熱を切る】診療で不明熱 | Modern Physician | 2016 |
| 2016162345 | 日本語 | 林 真由美, 康 | 冠動脈瘤を合併した若年性骨 | 日本小児科学会雑 | 2015 |
| 2016161845 | 日本語 | 小澤 貴暢, 吉竹 | リスクファクターのない若年女 | 茨城循環器研究会 | 2015 |
| 2015373940 | 日本語 | 東山 弘幸, 嶋尾 | Fragmentation hemolysisを呈 | 日本新生児成育医 | 2015 |
| 2015351820 | 英語 | Bera Debabrata, | Infective endarteritis in a cas | Journal of Cardiolo | 2015 |
| 2015259453 | 日本語 | 樋口 紘平, 五百 | 治療忌避のため8年間無治療 | 大阪小児科学会誌 | 2015 |
| 2015175709 | 日本語 | 森本 哲 | 【ICUで遭遇する血液疾患】(P | Intensivist | 2015 |
| 2015049498 | 英語 | Lin Yun-Ho, Lin | A Case Report of Scrub Typh | Japanese Journal | 2014 |
| 2014358201 | 英語 | Kiritani Sho, Kar | Multiple splenic nodules with | Clinical Journal of | 2013 |
| 2014358015 | 英語 | Gang Mi Hyeon, | Splenic infarction in a child wi | Pediatrics Internat | 2013 |
| 2014032387 | 日本語 | 宮前 多佳子, 井 | Chediak-Higashi症候群の血 | 日本臨床免疫学会 | 2013 |
| 2013353624 | 日本語 | 福島 隆治 | 実践 腹部エコー検査(第10回 | J-VET | 2013 |
| 2013201323 | 英語 | Agarwal Abhishe | Bilateral Subcapsular and Peri | Internal Medicine | 2012 |
| 2013185907 | 英語 | Park Seung Jin, | A case of splenic infarction p | Journal of Infectio | 2012 |
| 2013144556 | 日本語 | 吉竹 恵理, 岡村 | 人工膝関節全置換術後に敗血 | 麻酔 | 2012 |
| 2013119342 | 日本語 | 大賀 正一 | 【血球貪食症候群の病態と診 | 血液フロンティア | 2012 |
| 2013091260 | 日本語 | 小山 尚俊, 久保 | EBウイルス関連血球貪食症候 | 藤枝市立総合病院 | 2012 |
| 2013082072 | 日本語 | 松岡 信良, 村山 | 多発性大腸MALTリンパ腫にト | 診断と治療 | 2012 |
| 2012372825 | 英語 | 橋本 由起, 江野 | Leukocytoclastic Vasculltis w | 東邦医学会雑誌 | 2012 |

| | | | | | |
|---|---|---|---|---|---|
| 2012227871 | 英語 | Wada Yoshiro, K | Computed tomography finding | Pediatrics Internat | 2011 |
| 2012177757 | 日本語 | 藤岡 啓介, 西條 | 皮膚ランダム生検が診断に有 | 四国医学雑誌 | 2011 |
| 2012028642 | 日本語 | 蜂須賀 崇, 武内 | 悪性リンパ腫が疑われた脾梗 | 日本臨床外科学会 | 2011 |
| 2011253736 | 日本語 | 辻本 文雄 | 【腹痛の画像診断】各論 膵臓 | Medical Technolog | 2011 |
| 2011043587 | 日本語 | 保阪 由美子, 木 | 脾梗塞にて発見されたGemell | 感染症学雑誌 | 2010 |
| 2011007556 | 日本語 | 大賀 正一 | 【血液疾患の診かた 血液専門 | 治療 | 2010 |
| 2010237470 | 日本語 | 沼倉 忠久, 松浦 | インフリキシマブ療法中に発生 | 日本呼吸器学会雑 | 2010 |
| 2010180741 | 日本語 | 佐伯 百穂, 廻 勇 | 初診時より著明なleukoerythr | 岡山医療センター | 2010 |
| 2010099197 | 日本語 | 梶田 藍, 北川 正 | サイトメガロウイルス感染を契 | 岡山医療センター | 2008 |
| 2010039472 | 日本語 | 長井 瞳, 石山 泰 | 大動脈弁直下に生じた感染性 | 心臓 | 2009 |
| 2009321861 | 日本語 | 堤 久, 若杉 恵介 | 血球貪食症候群を伴った血管 | 老年者造血器疾患 | 2008 |
| 2009287951 | 日本語 | 河 敬世 | 【貧血を診る】血球貪食症候群 | 綜合臨床 | 2009 |
| 2009242224 | 日本語 | 大賀 正一, 田中 | 【免疫不全症候群と遺伝子異 | 臨床検査 | 2009 |
| 2009183798 | 日本語 | 義久 精臣, 宮田 | 心原性脾塞栓症による脾破裂 | 心臓 | 2009 |
| 2008344578 | 日本語 | 今井 利美, 西村 | 左心不全を合併したIntravasc | 日本内科学会雑誌 | 2008 |
| 2008287686 | 日本語 | 村瀬 卓平 | 【悪性リンパ腫診療update in 2 | 内科 | 2008 |
| 2008118951 | 日本語 | 喜安 嘉彦, 長沼 | 高齢で発症し急性肝障害から | 内科 | 2008 |
| 2008103703 | 日本語 | 辻本 文雄 | 【一般医のためのエコー活用 | Medicina | 2007 |
| 2008092966 | 日本語 | 辻本 文雄 | 間違いだらけの超音波検査・ | 綜合臨床 | 2008 |
| 2008058074 | 日本語 | 濱中 洋平, 鈴木 | 真性多血症から門脈閉塞をき | 日本門脈圧亢進症 | 2006 |
| 2008047953 | 英語 | Maruoka Hiroshi | Increased Splenic Fluorodeox | Internal Medicine | 2007 |
| 2008017007 | 英語 | Suzuki Yuhko, S | Splenic Infarction after Epste | International Jour | 2007 |
| 2007224400 | 日本語 | 仁多 美奈子, 新 | 血球貪食症候群を伴った急性 | 臨床血液 | 2007 |
| 2006233837 | 英語 | Natsume Hiromu | Splenic infarction in Letterer- | Pediatrics Internat | 2005 |
| 2006128939 | 日本語 | 伊藤 圭一, 村田 | 脾摘術にて肝不全を離脱し得 | 肝臓 | 2006 |
| 2006063328 | 日本語 | 渡部 拓, 深澤 雄 | 横紋筋融解症と急性腎不全を | 幌南病院医学雑誌 | 2005 |
| 2004311725 | 日本語 | 黒田 加奈子, 森 | インフルエンザAウイルス感染 | 日本検査血液学会 | 2004 |
| 2004229758 | 英語 | Belet Nursen, A | Two cases of congenital cyto | Pediatrics Internat | 2003 |
| 2004159330 | 英語 | Taguchi Akihiko, | Gamma/Delta T Cell Lymphom | Internal Medicine | 2004 |
| 2003200430 | 日本語 | 石井 亘, 島田 一 | 急性呼吸促迫症候群及び播種 | 内科 | 2003 |
| 2001153843 | 英語 | Osafune Kenji, T | Crescentic glomerulonephritis | Clinical and Experin | 2000 |
| 2001123147 | 日本語 | 金子 詩子, 鳥越 | 若年性関節リウマチと高サイト | 長岡赤十字病院医 | 2000 |
| 2001094027 | 日本語 | 稲毛 康司, 橋本 | 【小児呼吸器疾患の現況 特 | 日本胸部臨床 | 2000 |
| 1999215265 | 英語 | Huang Hsuan-L | 固有弁の感染性心内膜炎に合 | Japanese Circulati | 1999 |
| 1999077416 | 日本語 | 落海 剛彦, 今津 | 汎血球減少症と脾腫をきたした | 広島医学 | 1998 |
| 1999065419 | 英語 | Akdeniz Hayrett | 東アナトリアのVan地域におけ | Nagoya Medical Jo | 1998 |
| 1995070772 | 日本語 | 牟田 毅, 他 | 著明な肝脾腫および汎血球減 | 超音波医学 | 1994 |
| 1992188095 | 英語 | Saitoh Makiko, H | 幼児と小児における細菌性心 | Acta Paediatrica J | 1991 |
| 1991051827 | 日本語 | 高村 宏, 平沼 俊 | 末梢血および骨髄中に異型細 | 日大医学雑誌 | 1990 |
| 1990066159 | 英語 | Kobayashi Ichiro | 冠動脈瘤を合併した慢性Epste | Acta Paediatrica J | 1989 |
| 1989152264 | 日本語 | 高淵 洋彰, 白井 | 脾梗塞を伴った結核性脾膿瘍 | 最新医学 | 1989 |
| 1988157844 | 日本語 | 渡辺 尚彦, 田辺 | 救命し得た黄色ブドウ球菌性 | 感染症学雑誌 | 1987 |
| 1983111732 | 英語 | Feiz J., Hazeghi | 小児の重症ブドウ球菌性敗血 | Asian Medical Jour | 1980 |

| Volume | Pages | Pub. Type |
|---|---|---|
| 676回 | 52 | 会議録 |
| 18(1) | 36-41 | 原著論文 |
| 17(6) | 641 | レター |
| 17(6) | 640 | レター |
| 81(9) | 1889-1895 | 原著論文/ |
| 7(1) | 1 of 4-4 of | 原著論文/ |
| 67(5) | 289-292 | 原著論文/ |
| 64(3) | 302-304 | Q&A |
| 61(4) | 413-414 | 原著論文/ |
| 56(3) | 343-347 | 原著論文/ |
| 26(6) | 457-458 | 原著論文/ |
| 357回 | 35 | 会議録/症 |
| 58(13) | 1885-1889 | 原著論文/ |
| 48(2) | 132-136 | 原著論文/ |
| 4991 | 1-2 | 解説 |
| 24(5-6) | 479-482 | 原著論文/ |
| 14 | 295-297 | 原著論文/ |
| 67(5) | 585-590 | 原著論文/ |
| 17(5) | 147-150 | 原著論文/ |
| 18(1) | 37-41 | 原著論文/ |
| 38(4) | 745-748 | 原著論文/ |
| 18(6) | 414-417 | 原著論文/ |
| 7(1) | 99-103 | 原著論文/ |
| 107(2) | 269-273 | 原著論文/ |
| 26(1) | 64-68 | 原著論文/ |
| 17(3) | 434-440 | 解説 |
| 118(4) | 773-776 | 解説/特集 |
| 57(4) | 731-734 | 原著論文/ |
| 45(4) | 329-333 | 解説 |
| 36(4) | 315-321 | 解説/特集 |
| 119(12) | 1765-1771 | 原著論文/ |
| 22 | 27-33 | 原著論文/ |
| 27(2) | 275-279 | 原著論文/ |
| 11(1) | 21-24 | 原著論文/ |
| 32(2) | 8 | 会議録/症 |
| 7(2) | 343-351 | 総説/特集 |
| 67(2) | 115-117 | 原著論文/ |
| 6(6) | 434-437 | 原著論文/ |
| 55(5) | e126-e128 | 原著論文/ |
| 36(4) | 226-232 | 原著論文/ |
| 26(8) | 64-72 | 解説 |
| 51(9) | 1073-1076 | 原著論文/ |
| 18(6) | 945-947 | 原著論文/ |
| 61(12) | 1366-1368 | 原著論文/ |
| 23(1) | 69-75 | 解説/特集 |
| 18(1) | 11-14 | 原著論文/ |
| 100(10) | 1753-1757 | 原著論文/ |
| 59(5) | 236-240 | 原著論文/ |

| | | |
|---|---|---|
| 53(5) | 773-776 | 原著論文/ |
| 67(5-6) | 257-262 | 原著論文/ |
| 72(10) | 2649-2653 | 原著論文/ |
| 39(7) | 669-684 | 解説/特集 |
| 84(5) | 592-596 | 原著論文/ |
| 92(10) | 2401-2406 | 解説/特集 |
| 48(6) | 449-453 | 原著論文/ |
| 5 | 223-224 | 原著論文/ |
| 4 | 207-208 | 原著論文/ |
| 41(11) | 1257-1262 | 原著論文/ |
| 17(2) | 33-38 | 原著論文/ |
| 58(8) | 1759-1763 | 解説/特集 |
| 53(5) | 599-604 | 解説/特集 |
| 41(4) | 422-429 | 原著論文/ |
| 97(7) | 1666-1668 | 原著論文/ |
| 102(2) | 312-317 | 解説/特集 |
| 101(1) | 185-188 | 原著論文/ |
| 44(12) | 295-305 | 解説/特集 |
| 57(1) | 180-191 | 解説 |
| 12(4) | 291-295 | 原著論文/ |
| 46(12) | 909-911 | 原著論文/ |
| 85(5) | 380-383 | 原著論文/ |
| 48(4) | 310-314 | 原著論文/ |
| 47(3) | 329-332 | 原著論文/ |
| 47(1) | 30-37 | 原著論文/ |
| 2(1) | 44-49 | 原著論文/ |
| 5(2) | 201-206 | 原著論文/ |
| 45(5) | 593-594 | 原著論文/ |
| 43(2) | 120-125 | 原著論文/ |
| 91(3) | 570-573 | 原著論文/ |
| 4(4) | 329-334 | 原著論文/ |
| 13(1) | 23-28 | 原著論文/ |
| 59(増刊) | S170-S174 | 解説/特集 |
| 63(5) | 400-403 | 原著論文/ |
| 51(9) | 1169-1170 | 会議録/症 |
| 42(2) | 101-110 | 原著論文 |
| 21(10) | 660 | 会議録/症 |
| 33(5) | 613-616 | 原著論文 |
| 49(3) | 275-281 | 原著論文/ |
| 31(4) | 509-512 | 原著論文/ |
| 44(1) | 185-190 | 原著論文/ |
| 61(10) | 1153-1159 | 原著論文/ |
| 23(10) | 757-762 | 原著論文 |

Abstract

本邦において,成人でのサイトメガロウイルス(CMV)初感染による急性感染例が増加している。呈示

症例は70歳,男性.HIV陽性患者.腹痛とふらつきを主訴に受診し,CTで脾破裂が疑われた.外傷歴に
症例は15歳男児で、突然発症の心窩部痛、左側腹痛を訴えて当院を救急受診した。胸部所見で
54歳男。2年前から乏尿、労作性呼吸困難、腹部膨満、両下肢浮腫がみられた。20歳時に感染性
＜文献概要＞はじめに　血小板輸血後に血小板数の増加が不良な病態を血小板輸血不応(plate
症例は生後4ヵ月男児で、咳嗽と鼻漏がみられ、12日目から持続性発熱、皮疹、下痢が出現した
急性巨核芽球性白血病(AMKL)は新生児発生例が多く、原因遺伝子変異により予後に差があるこ
61歳男性。Stanford A型動脈解離に対し上行弓部大動脈置換術、オープンステントグラフト内挿
例報告
症例は85歳女性で、1週間前から発熱が続いており、さらに転倒により下肢を骨折し、心窩部痛と
症例は7歳男児で、父はインド系、母親は日本人であり、家族がインドから帰国した2週間後に発

56歳男。4歳時に種痘様水疱症と診断された。発熱のため受診した。呼吸困難のため入院し、肝
53歳男。前医でLDHとTPの高値を指摘され、当科に紹介された。全身の小リンパ節腫脹と軽度肝
症例は76歳女性。X年1月初めから断続的に発熱あり。徐々に食欲が低下してきたため、2月末に
症例は13歳女児で、7週前に倦怠感、身体痛、インフルエンザ様症状が出現し、4週前には外来で
症例は24歳男性で、両側の側頭部の頭痛と間欠的に生じる視力障害、および間欠熱がここ2週
症例は16歳の男性。多数回殴られて受傷。近医を受診後、当院へ転院搬送となった。造影CTで
症例は10歳の男児で、4日前から高熱、頭痛、倦怠感がみられ、2日前から鼻血と吐血が出現す
症例は83歳女性。主訴は発熱。胸部CTにてはじめは肺炎が疑われた。しかし、抗生剤の効果は
33歳男。1週間持続する39℃台の発熱と咽頭痛を主訴に他院受診し、細菌性咽頭炎の診断で抗
背景:腫瘍の診断には病理組織学的診断が必須であるが、重症のため生検ができない場合も少
輸血副作用には、溶血性副作用と非溶血性副作用がある。非溶血性副作用の中でもHLA抗原や
急性心筋梗塞,腹部大動脈瘤破裂,大動脈解離,肺動脈塞栓症の4つは,「救急外来でショック状態
慢性活動性Epstein-Barrウイルス感染症(CAEBV)に関連する肺動脈高血圧(PAH)および接合部
マクロファージ活性化症候群は全身炎症性疾患の代表的なものであり、主に全身型若年性特発
＜ポイント＞不明熱患者で咽後浮腫をみた場合、年長児や成人であっても川崎病を考慮すべき
1歳3ヵ月男児。頭部に水疱が出現し水痘と診断されたが、痂皮化した2日後に39℃の発熱と体幹
17歳女性。39℃の発熱が出現し近医を受診、感冒の診断で総合感冒薬を処方されたが発熱が
溶血性貧血の発症を契機に先天性サイトメガロウイルス(以下CMV)感染を診断した早産児の1例
症例は6歳男児で、4ヵ月間にわたり熱が続いており、複数の抗生物質を経験的に処方するも効
例報告
抗菌薬に不応性の持続性発熱をみたらHLHを鑑別に挙げ,フェリチンを測定する。HLHは,フェリチ
34歳女。発熱、右上腹部痛、右脇腹の鈍痛が1週間続いた後、敗血症性ショック、播種性血管内
症例は慢性リンパ性白血病(CLL)の既往を有する64歳男性で、著明な白血球増加症(163600/m
7歳女児。臍周囲および右上腹部の疼痛があった。肝機能異常および軽度の脾腫大を認め、原発
Chediak-Higashi症候群は原発性免疫不全症候群のひとつに分類され,わが国では約14例の報告

症例は小児期のサイトメガロウイルス感染による精神発達遅滞の22歳女性で、側腹部痛、体重減
症例は24歳男性。7日間継続する発熱、咽頭痛、筋痛と3日前からの左上腹部痛を主訴とした。
症例は58歳男性で、2年前に原発性マクログロブリン血症と診断され、1年前から両膝関節痛が
血球貪食症候群(HPS)は、免疫担当細胞の過剰な持続活性化を背景に、発熱、血球減少、肝脾
症例は2歳女児で、鼻汁、咳嗽と発熱で近医に抗生剤を処方されるも解熱せず、発熱5日目に紹
71歳男。約11年前より、糖尿病、アルコール性肝障害、高血圧症で通院中であった。8年前に便
白血球性破砕性血管炎は感染性心内膜炎、B型肝炎、C型肝炎、敗血症などの感染症により発

生後9日目に腹部膨満を呈し、健康状態不良の、妊娠35週で経腟分娩により出生した女児(体重
症例は62歳女性で、38℃台の発熱を認め、数ヵ月無治療で経過観察された。NSAIDsで経過を
症例は46歳,男性.3ヵ月前より全身倦怠感と微熱を認めていたが左側腹部に疼痛が出現したた

64歳男。嘔気、左季肋部痛と発熱で入院した。心尖部に最強点を有する汎収縮期雑音を認め、
＜プライマリ・ケアにおけるポイント＞こんな所見があれば本症を疑う・抗菌薬に不応の高熱が
クローン病患者のインフリキシマブ治療中に発症した血球貪食症候群(Hemophagocytic Syndro
80歳代女性。患者は下腿に紫斑が出現し、その後、微熱と全身倦怠感が次第に増悪し、近医で
41歳女。患者は全身の皮疹、関節痛、高熱、咽頭痛を主訴とした。脾腫および肝機能異常、高
症例は76歳、男性。61歳時に特発性血小板増多症の診断を受け当院血液内科通院中であった
症例は76歳女性。2007年4月に発熱で発症し、5月当科に入院。38℃台の発熱があり表在性リン
血球貪食症候群(Hemophagocytic syndrome;HPS)、または血球貪食性リンパ組織球症(Hemoph
血球貪食症候群/血球貪食性リンパ組織球症(HPS/HLH)は,発熱,血球減少,肝脾腫,播種性血管
症例は40歳代、男性。出血性脳梗塞を発症し前医に入院、同院での心エコー図検査にて僧帽弁
69歳男。患者は近医で左下肺野に浸潤影を指摘され、加療されたが発熱が持続し、便秘、下痢
血管内大細胞型B細胞リンパ腫(IVLBCL)は比較的まれであるが、中・高齢者に好発する予後不
75歳女性。患者は発熱、関節痛、脱水、食欲不振を主訴に著者らの施設へ入院となった。入院

54歳男。13年前に真性多血症の診断され、代謝拮抗薬によりコントロールされていた。9年前に
症例は64歳女性で、発熱と衰弱が1ヵ月以上持続し頻脈を呈した。細菌感染は否定され、腹部超
伝染性単核球症(IM)に引き続き脾梗塞が発現した遺伝性球状赤血球症(HS)の初症例(18歳、日
症例は19歳男性で、発熱と全身倦怠感の1ヵ月以上持続を主訴に来院、汎血球減少を認めた
1.5歳男児.蒼白,食欲不振および2週間続く熱で来院した.CTおよびMRIで巨脾腫と脾梗塞が認め
症例1(57歳男).全身倦怠感,黒色便を主訴とした.アルコール性肝硬変と診断され,外来にて経過
70歳男.65歳頃より毎日大量飲酒を続けていた.下痢・食事摂取不良に続き脱力と起立困難とな
25歳女.悪寒を伴う発熱,全身発疹を自覚した.入院後も高熱が持続し,高LDH血症,肝機能障害,血
症例1は22歳の初産婦から出生した2600gの女児で,生後2時間で口と鼻からの出血と皮膚の発
54歳女.発熱,肝脾腫を主訴とした.肝生検でシヌソイドにリンパ球浸潤を,また,腹腔鏡的切除脾の
31歳女.不明熱を主訴とした.白血球増加は認めなかったが発熱,関節症状,定型的皮疹,咽頭痛,リ
57歳男.4ヵ月前から倦怠感や食欲不振があり,脾腫や食道静脈瘤から門脈高血圧を疑われたが
症例1は10歳男で,耳痛,嘔吐,頭痛が出現し,不穏状態となった.2歳時に全身型若年性関節リウマ
アデノウイルス7型感染症は,発症年齢が1ヵ月から5歳前後にある.急性呼吸器感染症,咽頭結膜
19歳男.三尖弁,心室中隔欠損部,肺動脈弁を含む右心に感染性心内膜炎による巨大疣贅があり,
例報告
3年間にブルセラ症患者233例(男108例)を経験した.平均年齢は男で29.1歳,女で35.2歳であった
例報告
1950年から1989年までに男子15例,女子10例,計25例の細菌性心内膜炎を経験した.小児科の
57歳男,全身倦怠感,下痢,発熱のために入院した.理学的所見上,軽度の黄疸と肝腫が認められた
2歳男.発熱と咳が持続し,EBV感染が血清学的に証明された.口唇の発赤,苺舌,紅斑性発疹,頸部
71歳男,発熱を主訴とし,画像検査で脾膿瘍と診断したが,各種抗生剤が無効のため摘脾を行い,
症例は,23歳主婦,発熱にて入院.既往歴に,リウマチ熱,僧帽弁閉鎖不全症あり,入院時に,肝脾腫,
2年間に5～13歳のコアグラーゼ陽性黄色ブドウ球菌性敗血症を21例経験した.何れも基礎疾患

| Memo | Thesaurus |
|---|---|
| 示症例は31歳,男性。3週間持続する発熱,頭痛を認め,リンパ球の軽度増加,肝胆道系酵素上昇を合併してい | |
| | ＊巨脾症(外科的療法); 脂肪組織(外科的療法); ＊脾臓摘出術(有害作用); リンパ腫-びまん性大 |
| | ＊巨脾症(外科的療法,合併症); ＊脂肪組織(外科的療法); ＊脾臓摘出術(有害作用); リンパ腫-び |
| なく,非外傷 | ＊HIV感染症(合併症,薬物療法); 血腫(合併症,病理学,外科的療法); MRI; 鑑別診断; X線CT; 脾臓 |
| は異常なく, | Fosfomycin; 急性腹症(診断,薬物療法); MRI; 超音波診断; X線CT; ＊嚢胞(画像診断,薬物療法); |
| 生心内膜炎( | X線透視検査; ＊三尖弁狭窄症(病因,診断,治療); 術後合併症(診断,治療); 心エコー図; ＊バイオ |
| elet transfu | 血液型判定と血液交差適合試験; ＊血小板計数; HLA抗原; ＊血小板輸血; 治療成績 |
| 。21日目に | Ciclosporin(治療的利用); IgG(治療的利用); ＊NK細胞; ウイルス性疾患(診断,治療); 気道感染; |
| ことが判明し | 骨髄増殖性疾患; 鑑別診断; 転座; ＊白血病-急性巨核芽球性(診断,薬物療法,合併症); ヒト第1 |
| 術が施行さ | Ferritins(血液); Methylprednisolone(治療的利用); Triglycerides(血液); ＊術後合併症; ＊動脈瘤- |
| | 巨脾症(合併症); 抗凝固剤(治療的利用); ＊伝染性単核球症(病因); ＊脾臓梗塞(合併症,薬物療 |
| 左下肢痛を | 鑑別診断; 超音波診断; X線CT; ＊リンパ腫-びまん性大細胞型B細胞性(病理学,診断); ＊血管腫 |
| 熱、下痢、嘔 | Ceftriaxone(治療的利用); ＊腸チフス(合併症,診断,薬物療法); ＊DIC(合併症,診断,治療); 腹部X |
| | ＊巨脾症(X線診断,病因); 抗細菌剤(治療的利用); 心エコー図; ＊心内膜炎-感染性(超音波診断, |
| 脾腫、汎血 | Ciclosporin(治療的利用); Dexamethasone(治療的利用); Doxorubicin(治療的利用); Etoposide( |
| 脾腫を認め | Asparaginase(治療的利用); Cytarabine(治療的利用); Etoposide(治療的利用); Methylprednisolo |
| 入院。身体 | 骨髄検査; 生検; ＊変異; 免疫組織化学; ＊リンパ腫-びまん性大細胞型B細胞性(診断,病理学); |
| で食欲不振、 | Vancomycin(治療的利用); ＊心内膜炎-感染性(超音波診断,薬物療法,合併症); 僧帽弁閉鎖不 |
| 間で悪化して | Ceftriaxone(治療的利用); ＊Leuconostoc; 肝炎-C型(合併症); クモ膜下出血(合併症,画像診断 |
| IIIb型脾損傷 | ヒトヘルペスウイルス4型; 巨脾症(X線診断,病因); ウイルス抗体; X線CT; ＊伝染性単核球症(診 |
| るようになっ | ELISA; ヘマトクリット; 鑑別診断; 等張液(治療的利用); 吐血(診断,薬物療法,予後); 腹痛(診断,薬 |
| 不良であり、 | 肝臓(病理学); 鑑別診断; 生検; ＊結核-粟粒(診断,病理学); 肉芽腫(診断,病理学); ＊不明熱(診断 |
| 生物質を投 | ウイルスDNA(血液); Lupus Coagulation Inhibitor(血液); Prednisolone(治療的利用); 血液化学 |
| なくない。著 | ＊意識障害(病因,遺伝学,病理学); myc遺伝子; 肝腫大(病因,遺伝学,病理学); 巨脾症(病因,遺伝 |
| HLA抗体が | 移植片対宿主病(免疫学); ＊血液型判定と血液交差適合試験; 血液製剤; ＊HLA抗原; ＊輸血(有 |
| になり死に | ポルフィリン症(合併症,診断); 巨脾症(合併症,診断); 自己免疫疾患(合併症,診断); 心筋梗塞(合 |
| 異所性頻拍 | 肝腫大(診断,合併症); 冠状動脈瘤(超音波診断,合併症); 巨脾症(診断,合併症); 心エコー図; 心 |
| 性関節炎の | バイオマーカー; C-Reactive Protein; Interferon Gamma; Interleukin-6(血液); Steroids(治療 |
| である。不明 | 咽頭疾患(病因,X線診断); 巨脾症(病因,X線診断); クモ膜下出血(病因,X線診断); 梗塞(病因,X線 |
| に紅斑が出 | Ciclosporin(治療的利用,毒性・副作用); Granulocyte-Macrophage Colony-Stimulating Factor( |
| 持続するた | Cefazolin(治療的利用); Gentamicins(治療的利用); Staphylococcus aureus; 危険因子; ＊三尖 |
| を経験した。 | メトヘモグロビン血症(診断,合併症); 肝腫大(診断,合併症); ＊サイトメガロウイルス感染症(診断, |
| 果がみられ | Gentamicins(治療的利用); ＊心内膜炎-感染性(合併症,超音波診断,薬物療法); ペニシリン耐性; |
| | 巨脾症(病因,X線診断,外科的療法); ＊診療拒否(患者側); 脾動脈(外科的療法,X線診断); リンパ |
| ン高値,可溶 | Ciclosporin(治療的利用); Dexamethasone(治療的利用); Etoposide(治療的利用); ＊Ferritins(血 |
| 凝固症候群 | Ceftriaxone(治療的利用); Minocycline(治療的利用); 血液透析; 骨髄検査; ＊ツツガムシ病(合併 |
| m3)と進行 | Cyclophosphamide(治療的利用); ＊Propionibacterium acnes; 巨脾症(病因); 腫瘍多剤併用療法 |
| 発性Epstein | ＊ヒトヘルペスウイルス4型; Protein C; 肝機能検査; 肝臓疾患(病因,診断); 巨脾症(治療,X線診 |
| があるに過 | ＊Chediak-Higashi症候群(合併症,診断,治療); 肝腫大(病因); 巨脾症(病因); 毛の色; 肛門疾患( |
| | イヌの疾病(超音波診断); 巨脾症(超音波診断); 血管肉腫(超音波診断); 血腫(超音波診断); ＊超 |
| 減少および | Cyclophosphamide(治療的利用); Methylprednisolone(治療的利用); 血管造影; ＊血腫(病因,X線 |
| 血液検査で | ELISA; IgM(血液); ＊マイコプラズマ感染症(合併症,診断,薬物療法); ＊Mycoplasma pneumoniae; |
| 出現し、1ヵ月 | ＊マクログロブリン血症(合併症); 脛骨骨折(合併症,外科的療法); ＊ショック-敗血症性(病因); 致 |
| 腫、播種性 | Ciclosporin(治療的利用); Etoposide(治療的利用); 同種移植; 遺伝性疾患(治療,薬物療法); 自己 |
| 介受診し、血 | Aciclovir(治療的利用); ウイルスDNA; ヒトヘルペスウイルス4型; ＊Gamma-Globulins(治療的利 |
| 潜血陽性で | Cyclophosphamide(治療的利用); ＊ヘリコバクター感染症(合併症,薬物療法); Helicobacter pylor |
| 症する疾患 | IgA血管炎; 鑑別診断; ＊心内膜炎-感染性(合併症); 生検; 直接蛍光抗体法; ＊血管炎-皮膚白血 |

| | |
|---|---|
| 2370g)症例 | Aciclovir(治療的利用); Alanine Transaminase(血液); Aspartate Aminotransferases(血液); L-La |
| られていた | 鑑別診断; *生検; 皮膚(病理学); 免疫組織化学; *リンパ腫-びまん性大細胞型B細胞性(病理学 |
| 当院を受診 | 鑑別診断; 超音波診断; X線CT; *脾臓梗塞(画像診断,外科的療法,病理学); 脾臓摘出術; リンパ |
| | 巨脾症(合併症); 自己免疫疾患(合併症); 超音波診断; 膵炎(合併症); *膵臓疾患(X線診断,超音 |
| 白血球増加、 | Ceftriaxone(治療的利用); Gentamicins(治療的利用); Penicillin G(治療的利用); 眼症状; *心内膜 |
| 持続し、血球 | ヒトヘルペスウイルス4型; *プライマリヘルスケア; アルゴリズム; 診療ガイドライン; Epstein-Barr |
| ne:以下HPS | Crohn病(診断,薬物療法); Ethambutol(治療的利用); Gamma-Globulins(治療的利用); Isoniazid(治 |
| 著明な白血 | DIC(病因); *白血病-慢性骨髄単球性(合併症); *貧血-骨髄癆性(病因); 致死的転帰 |
| ェリチン血症 | Prednisolone(治療的利用,毒性・副作用); *Still病-成人(合併症,薬物療法); *サイトメガロウイル |
| 。2007年5月 | Vancomycin(治療的利用); *医用材料; 心エコー図; *心内膜炎-感染性(超音波診断,合併症,外 |
| パ節腫大や | Methylprednisolone(治療的利用); リンパ腫-B細胞性(合併症,病理学); リンパ腫-びまん性大細 |
| agocytic lym | Ciclosporin(治療的利用); ヒトヘルペスウイルス4型; Etoposide(治療的利用); Prednisolone(治療 |
| 内凝固およ | Ciclosporin(治療的利用); フローサイトメトリー; Gamma-Globulins(治療的利用); NK細胞; 組織球 |
| 前尖および | 心エコー図; *心内膜炎-感染性(合併症,診断); 僧帽弁閉鎖不全症(合併症,超音波診断); *塞栓 |
| を繰返すよう | Cyclophosphamide(治療的利用); Prednisolone(治療的利用); Vincristine(治療的利用); 腫瘍多剤 |
| 良のびまん | Cyclophosphamide(治療的利用); Doxorubicin(治療的利用); Prednisolone(治療的利用); Vincris |
| 等、皮膚所見 | Ferritins(血液); Gamma-Globulins(治療的利用); Methylprednisolone(治療的利用); Prednisolon |
| | 局所解剖学; 過誤腫(超音波診断); *巨脾症(超音波診断); 血管腫(超音波診断); 鑑別診断; *超 |
| | *巨脾症(超音波診断); 超音波診断; *胆道ジスキネジア(超音波診断); 膿瘍(超音波診断); 脾臓 |
| 脾腫がみられ | Hydroxyurea(治療的利用); Pseudomonas aeruginosa; シュードモナス感染症(合併症); 血管疾患 |
| 音波、CT像 | *巨脾症(放射性核種診断,病理学); *脾臓疾患(放射性核種診断,病理学); 抗好中球細胞質抗体 |
| 本人男性)を | IgM(血液); *球状赤血球症-遺伝性(合併症); ウイルス抗原; X線CT; *脾臓梗塞(合併症,X線診断 |
| め緊急入院 | Dexamethasone(治療的利用); Etoposide(治療的利用); Ferritins(血液); Tretinoin(治療的利用); |
| られた.ガリウ | Gallium Isotopes; 巨脾症(外科的療法,病因,画像診断); MRI; *組織球症-Langerhans細胞(外科 |
| 観察中であっ | *肝硬変(合併症,外科的療法); 肝硬変-アルコール性(合併症,外科的療法); 脾機能亢進(合併症 |
| 緊急入院と | Myoglobin; アルコール依存症; 栄養障害; *横紋筋融解症(合併症,病理学); *肝硬変-アルコール |
| 球減少を認 | Ferritins(血液); インフルエンザ-ヒト(合併症); *インフルエンザウイルスA型; プロトロンビン時間; |
| 赤により入院 | *サイトメガロウイルス感染症; 新生児疾患; DIC; 感染症垂直伝播 |
| 脾髄にリン | ウイルスDNA; *肝臓腫瘍(合併症,診断,薬物療法); 腫瘍多剤併用療法; 脾臓腫瘍(合併症,診断, |
| ンパ節腫脹 | Steroids(治療的利用); *Still病-成人(薬物療法,病因); 胸水; 胸部X線診断; 抗核抗体; 呼吸窮迫 |
| ,腎不全が急 | 外科手術; 糸球体腎炎; 腎炎; 心内膜炎-感染性(合併症); 糸球体腎炎-急速進行性(合併症,外科 |
| チ(JRA)と診 | Cytokines; 関節炎-若年性(診断,治療,免疫学); 血液タンパク質障害; 組織球症-非Langerhans細 |
| 熱,急性胃腸 | ヒトアデノウイルス感染症; 気道感染; 気道疾患; 小児科学 |
| 赤血球破壊 | 心エコー図; 心内膜炎-感染性(病態生理学,超音波診断,合併症); 貧血-溶血性(合併症) |
| | 巨脾症; 心内膜炎-感染性; 汎血球減少症 |
| .症状は発熱 | ブルセラ症; トルコ |
| | 肝腫大; 心内膜炎-感染性(合併症); 汎血球減少症; 脾臓疾患 |
| 入院1,000例 | 心内膜炎-感染性 |
| が,脾腫はな | 血液; 骨髄; 組織球症(病理学); 死後検査 |
| リンパ節腫脹 | ヒトヘルペスウイルス4型(炎症・感染・感染症); 冠動脈疾患 |
| 摘出脾臓の病 | 結核-脾臓(画像診断,合併症); 超音波診断; 膿瘍(合併症); 脾臓梗塞(合併症) |
| Osler痛斑,Ja | 心内膜炎-感染性; ブドウ球菌感染症 |
| や易感染性 | ブドウ球菌感染症; 敗血症 |

たが,咽頭炎や頸部リンパ節腫脹は目立たなかった。不明熱の精査目的で当院に入院し,CMV感染による伝
細胞型B細胞性; 血管腫瘍(診断); 肺炎球菌ワクチン(治療的利用); *術後感染症(予防)
まん性大細胞型B細胞性(診断); *血管腫瘍(診断); *術後感染症(予防)
疾患(合併症,病理学,外科的療法); 脾臓摘出術; *脾臓破裂(合併症,病理学,外科的療法)
*脾臓疾患(画像診断,薬物療法)
プロテーゼ(有害作用); *人工心臓弁(有害作用); *人工弁置換術(有害作用); *バルーン弁形成術

自然寛解; *血球貪食性リンパ組織球症(免疫学,薬物療法,予後); 静脈内注入
染色体; ヒト第22染色体; 融合癌遺伝子タンパク質; 細胞遺伝学的分析
解離性(外科的療法); *大動脈瘤-胸部(外科的療法); *大動脈置換術(有害作用); *血球貪食性リンパ組織
去); *Epstein-Barrウイルス感染症(合併症)
瘍(病理学,診断); 致死的転帰; 胆嚢炎-無石; 剖検
線診断; *敗血症(合併症,診断,薬物療法); インド; 輸入感染症(診断,薬物療法)
薬物療法,合併症); X線CT; *発熱(病因); 細菌培養; 血液培養
治療的利用); Prednisolone(治療的利用); 肝臓疾患(診断,薬物療法,合併症); 消化管出血(化学的誘発); DIC(
ne(治療的利用); Prednisolone(治療的利用); 多剤併用療法; *リンパ増殖性疾患(診断,薬物療法,治療); *Eps
CD20抗原; *血管腫瘍(診断,病理学); 致死的転帰; *遺伝学的検査; *Myeloid Differentiation Factor 88
全症(超音波診断,病因); *リウマチ熱(診断,薬物療法,合併症); 経食道心エコー図; Sultamicillin(治療的利用);
); 失明(病因,診断); 心エコー図; 心臓疾患-先天性(合併症,超音波診断,外科的療法); *心内膜炎-感染性(合
断,合併症); *脾臓疾患(X線診断,合併症); *腹部外傷(X線診断,合併症)
物療法,予後); クラスター分析; ウイルス非構造タンパク質(血液); *重症デング(遺伝学,診断,薬物療法); RT-
)
分析; 生検; X線CT; *伝染性単核球症(合併症,診断,薬物療法); *脾臓梗塞(病因,X線診断,薬物療法); リンパ節
学,病理学); 鑑別診断; 転座; *DIC(病因,遺伝学,病理学); マクロファージ(病理学); 免疫組織化学; *リンパ腫-
害作用); 血小板輸血(有害作用); 輸血関連急性肺障害(免疫学)
併症,診断); 大動脈破裂(合併症,診断); 動脈瘤-解離性(合併症,診断); 内分泌系疾患(合併症,診断); 鉛中毒(
電図; *肺高血圧症(診断,治療,合併症); 致死的転帰; 頻拍-異所性接合部(診断,合併症); *Epstein-Barrウイ
利用); 血小板計数; 抗凝固剤(治療的利用); 赤血球沈降速度; 発熱; 腫瘍壊死因子アルファ; *マクロファージ
診断); 腎臓疾患(病因,X線診断); 心内膜炎-感染性(合併症); 浮腫(病因,X線診断); 高安動脈炎(合併症); *X
治療的利用); IgG(治療的利用); Prednisolone(治療的利用); *冠状動脈瘤(超音波診断,病因); MRI; Interleuki
(外科的療法,超音波診断); 心エコー図; *心内膜炎-感染性(病因,治療,診断); 多剤併用療法; *ブドウ球菌感
併症); 巨脾症(診断,合併症); 血小板減少症(診断,合併症); *貧血-溶血性(診断,治療,合併症); *未熟児疾患
経食道心エコー図; *大動脈弁上狭窄症(合併症,超音波診断); Enterococcus Infection(合併症,診断,薬物
増殖性疾患(診断,治療); 致死的転帰; 動脈瘤破裂(外科的療法,病因); *Epstein-Barrウイルス感染症(診断,合
液); NK細胞; 組織球症-非Langerhans細胞(合併症,診断,薬物療法); 鑑別診断; *発熱(病因,診断,薬物療法);
症,診断,治療); 脳出血(合併症,病因); DIC(合併症); 致死的転帰; 全身性炎症反応症候群(合併症); *血球貪食
; 腫瘍再発; 多剤併用療法; X線CT; *膿瘍(病因,診断,外科的療法); *白血病-慢性B細胞性(合併症,薬物療法
); 血液凝固; 鑑別診断; X線CT; *伝染性単核球症(合併症,診断); 脾臓梗塞(病因,診断,治療); Protein S; *血
因); 骨髄移植; 骨髄検査; Interleukin-2 Receptors(血液); 膿瘍(病因); 発熱(病因); *血球貪食性リンパ組織
音波診断; 石灰沈着症(超音波診断); 捻転(超音波診断); 嚢胞(超音波診断); 膿瘍(超音波診断); 脾臓梗塞(超
診断); 後腹膜腔(X線診断); 知的障害(合併症); 塞栓術; 多剤併用療法; 多臓器不全(病因); X線CT; *多発動
肝腫大(病因,X線診断); 巨脾症(病因,X線診断); ウイルス抗体(血液); X線CT; *脾臓梗塞(病因,X線診断,薬物
死的転帰; 周術期管理; *膝関節置換術(有害作用); 剖検
免疫疾患(合併症); 免疫学的因子(治療的利用); リンパ腫(合併症); 造血幹細胞移植; Epstein-Barrウイルス
); *Prednisolone(治療的利用); ウイルス抗体; 骨髄検査; *Epstein-Barrウイルス感染症(薬物療法,診断,合
i; Prednisolone(治療的利用); Vincristine(治療的利用); *回腸腫瘍(放射性核種診断,薬物療法); 腫瘍多剤併
球破砕性(病理学,合併症); 致死的転帰

ctate Dehydrogenase(血液); *肝臓疾患(病因,X線診断); *新生児疾患; 全血交換輸血; *X線CT; *単純ヘル
[illegible]); *血管腫瘍(病理学)
腫; Fluorodeoxyglucose F18(診断的利用); 陽電子放射型断層撮影
波診断,合併症); 膵臓腫瘍(合併症); X線CT; 膿瘍(合併症); 脾臓梗塞(合併症); *脾臓疾患(X線診断,超音波
莫炎-感染性(合併症,薬物療法); X線CT; 投薬計画; 微生物薬物感受性試験; *脾臓梗塞(病因); 網膜出血; *
rウイルス感染症(合併症); *血球貪食性リンパ組織球症(病因)
治療的利用); Methylprednisolone(治療的利用); Rifampicin(治療的利用); Streptomycin(治療的利用); 多剤併

ス感染症(合併症); 血小板減少症(病因,化学的誘発); 骨髄検査; *多臓器不全(病因,化学的誘発); 致死的転
科的療法); 心膜; 大動脈弁(超音波診断,外科的療法); *大動脈瘤(超音波診断,合併症,外科的療法); *動脈
胞型B細胞性(合併症,病理学); *血管腫瘍(合併症,病理学); 致死的転帰; パルス療法(薬物療法); *血球貪食
的利用); 同種移植; 多剤併用療法; 造血幹細胞移植; Epstein-Barrウイルス感染症(合併症); 臍帯血幹細胞
; 発生率; 細胞傷害性T細胞; リンパ球活性化; 配列分析; *血球貪食性リンパ組織球症(診断,薬物療法,疫学
症(合併症,病理学); DIC(合併症,診断); *脾臓破裂(病因,病理学); 腹腔内出血(病因,病理学); 致死的転帰; 敗
剤併用療法; 生検; *リンパ腫-びまん性大細胞型B細胞性(合併症,診断,薬物療法); *血管腫瘍(合併症,診断
stine(治療的利用); 腫瘍多剤併用療法; 分類; *血管腫瘍(薬物療法); Rituximab(治療的利用)
e(治療的利用); *Still病-成人(合併症,診断,薬物療法); *肝臓疾患(合併症,治療); 血液製剤; 血漿; *DIC(合併
音波診断; 嚢胞(超音波診断); 膿瘍(超音波診断); *脾臓梗塞(超音波診断); *脾臓疾患(超音波診断); *脾臓
梗塞(超音波診断); 脾臓疾患(超音波診断); 脾臓破裂(超音波診断); 内臓錯位症候群(超音波診断)
息(合併症,病理学); 血小板計数; 呼吸窮迫症候群-急性(病因); 原発性骨髄線維症(合併症,病理学); 食道胃
; *Fluorodeoxyglucose F18(診断的利用,薬理学); *血管炎-中枢神経系(放射性核種診断,病理学); 陽電子
f); *Epstein-Barrウイルス感染症(合併症,診断)
肝腫大(合併症); 巨脾症(合併症); 血小板減少症(合併症); 抗腫瘍剤(治療的利用); 骨髄検査; 皮下出血(合
的療法,合併症); X線CT; *脾臓梗塞(外科的療法,病因,画像診断); 脾臓摘出術; 放射性核種イメージング
,外科的療法); 脾臓摘出術; 肝炎-B型-慢性(合併症,外科的療法)
レ性(合併症,病理学); 急性腎障害(合併症,病理学); 死後検査; 免疫組織化学
*組織球症-非Langerhans細胞(病因,診断); Interleukin-2 Receptors(血液); 赤血球凝集抑制試験; 組織球

薬物療法); *リンパ腫-T細胞性(合併症,診断,薬物療法); ガンマ-デルタT細胞抗原受容体; Epstein-Barrウイ
症候群-急性; DIC; パルス療法(薬物療法)
科的療法)
細胞(診断,治療,免疫学); 症候群; 診断; 分類; マクロファージ活性化; マクロファージ活性化症候群(免疫学)

染性単核球症と診断した。肝脾腫を認め,無症状の脾梗塞を合併した。経過観察で脾梗塞の増悪はなく,1ヵ月

球症(病因,診断,薬物療法); ステントグラフト内挿術(有害作用)

診断,治療,合併症); 貧血(診断,治療,合併症); 酸素欠乏(診断,治療,合併症); リンパ増殖性疾患(診断,薬物療法
stein-Barrウイルス感染症(診断,薬物療法,治療); 臍帯血幹細胞移植

心不全-収縮期(超音波診断,病因)
併症,超音波診断,治療); 急性腎障害(合併症,診断,治療); 心膜液貯留(合併症,超音波診断,外科的療法); 大

PCR法; 外国人; フィリピン; *輸入感染症(遺伝学,診断,薬物療法); 分子タイピング

節炎(病因,病理学,薬物療法)
びまん性大細胞型B細胞性(合併症,遺伝学,病理学); FISH法; 致死的転帰; *肝機能障害(病因,遺伝学,病理

合併症); 肺塞栓症(合併症,診断); 泌尿生殖器奇形(合併症,診断); *腹痛(病因,診断)
ルス感染症(診断,治療,合併症); 末梢血幹細胞移植
ジ活性化症候群(診断,薬物療法); Interleukin-1 Beta(血液)
線CT; 脳梗塞(病因,X線診断); 肺高血圧症(病因,X線診断); 脾臓梗塞(病因,X線診断); 川崎病(合併症); 副腎
in-2 Receptors(血液); 心エコー図; 脳浮腫(画像診断,化学的誘発); 膜タンパク質; 致死的転帰; 全身性炎症
感染症(合併症,薬物療法,診断); 細菌培養; Levofloxacin(治療的利用); 血液培養
; 赤血球輸血
療法); 細菌培養; 血液培養
併症,治療); *待機療法
マクロファージ活性化; T細胞; リンパ球活性化; Epstein-Barrウイルス感染症(合併症,診断,薬物療法); *血球
食性リンパ組織球症(病因,診断,治療)
法); *発熱(病因,薬物療法); *脾臓疾患(病因,診断,外科的療法); 脾臓摘出術; *グラム陽性細菌感染症(合併
栓形成傾向(合併症,診断)
球症(病因)
超音波診断); *脾臓疾患(超音波診断); 脾臓破裂(超音波診断); 分離腫(超音波診断); リンパ腫(超音波診断)
脈炎-結節性(X線診断,合併症,外科的療法); DIC(病因); *腹腔内出血(病因,X線診断); 致死的転帰; 静脈内
療法); Azithromycin(治療的利用)

感染症(合併症); *血球貪食性リンパ組織球症(治療,薬物療法,病因)
併症); *血球貪食性リンパ組織球症(薬物療法,病理学,病因)
用療法; 腫瘍再発; 生検; *大腸腫瘍(合併症,診断,薬物療法); 多剤併用療法; *リンパ腫(放射性核種診断,薬

ペス(合併症,診断,薬物療法); ヒトヘルペスウイルス2型; *血球貪食性リンパ組織球症(病因,X線診断,治療)

診断,合併症); 脾臓破裂(合併症); *腹痛(病因)
グラム陽性細菌感染症(合併症,薬物療法); 経食道心エコー図; *Gemella; 血液培養

用療法; *結核-肺(化学的誘発,診断,薬物療法); パルス療法(薬物療法); *Infliximab(治療的利用,毒性・副作

帰; パルス療法(薬物療法); *血球貪食性リンパ組織球症(病因,化学的誘発,診断)
瘤-感染性(超音波診断,合併症,外科的療法); Fibrin Tissue Adhesive(治療的利用); 心臓弁形成術
性リンパ組織球症(合併症,薬物療法); 剖検
移植; *血球貪食性リンパ組織球症(病因,治療,薬物療法)
); Perforin
血症(合併症,診断)
,薬物療法); *左心室機能障害(合併症); 致死的転帰

症,治療); *血球貪食性リンパ組織球症(合併症,薬物療法)
腫瘍(転移性,超音波診断); 脾臓破裂(超音波診断,病因); *分離腫(超音波診断); リンパ管腫(超音波診断); リン

静脈瘤(合併症,X線診断); *塞栓術; *多血症-真性(薬物療法,合併症); 消化管内視鏡法; *破裂-自然(合併症
放射型断層撮影; 血管炎-抗好中球細胞質抗体関連(放射性核種診断,病理学)

併症); DIC(薬物療法,合併症); *白血病-前骨髄球性(合併症,薬物療法); 発熱(合併症); 汎血球減少症(合併症

; X線CT; 白血球計数

ルス感染症(合併症,診断); 細胞遺伝学的分析; Vascular Endothelial Growth Factors(血液)

月後に改善を確認した。CMV単核球症はEpstein-Barr virus(EBV)による伝染性単核球症に比べて非典型的

去,合併症); 致死的転帰; *Epstein-Barrウイルス感染症(診断,薬物療法,合併症); 無症候性疾患; 臨床的増悪

動脈弁狭窄症(合併症,超音波診断,治療); 大動脈弁閉鎖不全症(合併症,超音波診断,治療); 脾臓梗塞(合併症

学); *剖検; リンパ節症(病因,遺伝学,病理学)

疾患(病因,X線診断); *不明熱(病因); リンパ腫-びまん性大細胞型B細胞性(合併症); 血管腫瘍(合併症); 大
反応症候群(病因); GTP Phosphohydrolases; 遺伝学的検査; 血球貪食性リンパ組織球症(薬物療法,病因); *

求貪食性リンパ組織球症(病因,診断,薬物療法)

症,薬物療法); Fluorodeoxyglucose F18(診断的利用); Fludarabine(治療的利用); Rituximab(治療的利用); 陽

殳与

薬物療法); 致死的転帰; *リンパ腫-辺縁帯B細胞性(合併症,診断,薬物療法); Pirarubicin(治療的利用); Rituxi

用); *血球貪食性リンパ組織球症(化学的誘発,診断,薬物療法)

ンパ腫(超音波診断); 超音波プローブ; 内臓錯位症候群(超音波診断)

E,外科的療法); 脾臓梗塞(合併症,X線診断); 腹水症(合併症,X線診断,外科的療法); *腹腔静脈短絡術; 致死

E); 貧血(合併症); 先天性低フィブリノーゲン血症(合併症); 網膜出血(合併症); Gabexate(治療的利用); *血球

であることが多く,健常成人の不明熱では鑑別にCMV感染を挙げる必要がある。また,脾梗塞を合併することが[illegible]

[illegible]

[illegible]症,画像診断); 網膜動脈閉塞症(合併症,画像診断); Daptomycin(治療的利用); *物質乱用-静脈内(合併症); [illegible]

[illegible]脳出血(病因,X線診断); 腫脹(病因,X線診断); 肺梗塞(病因,X線診断)
[illegible]白血病-若年性骨髄単球性(診断,薬物療法,合併症)

[illegible]電子放射型断層撮影; インターフェロンガンマ遊離試験

[illegible]mab(治療的利用); 除菌療法

的転帰; 剖検

[illegible]貪食性リンパ組織球症(合併症)

があり,その検出にはD-dimer測定と,腹部CTあるいは腹部エコーによるスクリーニングが有用と考えられた。(

グラム陽性細菌感染症(合併症,診断,治療); 経食道心エコー図; 細菌培養; 血液培養

著者抄録）

| ID | Language | Authors | Title | Journal | Year |
|---|---|---|---|---|---|

| Volume | Pages | Pub. Type | Abstract | Memo |
|---|---|---|---|---|

| ID | Language | Authors | Title |
|---|---|---|---|

| Journal | Year | Volume | Pages | Pub. Type | Abstract | Memo |
|---|---|---|---|---|---|---|

| ID | Langu | Authors | Title | Journal | Year | Volume |
|---|---|---|---|---|---|---|

| Pages | Pub. Type | Abstract | Memo |
| --- | --- | --- | --- |

| CQ番号 | CQ名 | 検索式 | 文献数 | 検索DB |
|---|---|---|---|---|
| | | ((巨脾症/TH or 脾腫/AL)) and (((人工血管/TH or 人工血管/AL)) or ((心内膜炎/TH or 心内膜炎/AL)) or ((動脈瘤/TH or 動脈瘤/AL)) or ((人工ペースメーカー/TH or ペースメーカ/AL)) or (血管内/AL) or ((脾臓梗塞/TH or 脾梗塞/AL))) and (((菌血症/TH or 菌血症/AL)) or ((細菌感染症/TH or 細菌感染症/AL)) or ((感染/TH or 感染/AL))) | 91 | その他（DB名 |
| | | | 0 | |
| | | | 0 | |
| | | | 0 | |
| | #1 | (巨脾症/TH or 脾腫/AL) | 6,898 | |
| | #2 | (菌血症/TH or 菌血症/AL) | 12,113 | |
| | #3 | #1 and #2 | 14 | |
| | #4 | (細菌感染症/TH or 細菌感染症/AL) | 287,208 | |
| | #5 | (人工血管/TH or 人工血管/AL) | 19,003 | |
| | #6 | (心内膜炎/TH or 心内膜炎/AL) | 16,345 | |
| | #7 | (動脈瘤/TH or 動脈瘤/AL) | 132,829 | |
| | #8 | (人工ペースメーカー/TH or ペースメーカ/AL) | 15,876 | |
| | #9 | 血管内/AL | 111,870 | |
| | #10 | (脾臓梗塞/TH or 脾梗塞/AL) | 891 | |
| | #11 | #5 or #6 or #7 or #8 or #9 or #10 | 270,660 | |
| | #12 | (感染/TH or 感染/AL) | 885,006 | |
| | #13 | #2 or #4 or #12 | 943,690 | |
| | #14 | #1 and #11 and #13 | 91 | |

| 検索担当者 | 検索実行日 | 保存ファイル名 | メモ |
| --- | --- | --- | --- |

[illegible]入力）

PubMed 医中誌 Cochrane | JMEDPlus
