# Supplementary material for: First case report of splenomegaly with splenic infarction due to aortic graft infection
Source: BMC Cardiovasc Disord. 2023 May 5;23:237. doi: 10.1186/s12872-023-03259-y (PMC10161471; doi:10.1186/s12872-023-03259-y)
Supplement: Supplementary file 1 — Additional file 1. [file 12872_2023_3259_MOESM1_ESM.zip › Splenomegaly_Pubmed_ESM.pdf]

ID	Language	Authors	Title	Journal	Year
34178234	eng	Ewa AU, Ochan	Tatumella ptyseos septicaemi	Pan Afr Med J	2021
33267828	eng	Im JH, Chung M	Splenic infarction and infectio	BMC Infect Dis	2020
33046510	eng	Loomis WP, Del	Failure of CD4 T Cell-Deficier	Infect Immun	2020
32655567	eng	Park S, Jung B,	Salmonella Typhimurium Lacki	Front Immunol	2020
31173526	eng	Toral M, Robles-	Lactobacillus fermentum CEC	FASEB J	2019
30989418	eng	Inan A, Erdem H	Brucellosis in pregnancy: resu	Eur J Clin Microbic	2019
29753533	spa	Gómez H, Gonz	Massive splenic abscess seco	Gastroenterol Hep	2018
28775247	eng	Fareed S, Nashv	Spinal Abscess Caused by Sal	Am J Case Rep	2017
28569671	eng	Rahim MI, Babba	Degradable magnesium implan	Biomed Mater	2017
28369700	eng	Huang A, Fiador	In utero presentation of aggre	Br J Dermatol	2017
27894360	eng	Maclean L, Abou	The relationship of endotoxae	Parasitology	2017
27032517	eng	Tennant SM, Ma	Nontyphoidal salmonella disea	Vaccine	2016
26499862	eng	Mouri O, Benhai	Spontaneous remission of full	BMC Infect Dis	2015
25856341	eng	Nielsen MV, Am	Clinical indicators for bacteria	PLoS One	2015
25531235	eng	Villa G, D'Alfons	Role of hemodialysis with high	Blood Purif	2014
24697022	eng	Mikić D, Djordjev	Disseminated Rhodococcus e	Vojnosanit Pregl	2014
23867185	eng	D'Angiò M, Cegli	Visceral leishmaniasis present	Blood Transfus	2014
23808943	eng	Valdés-Ferrer S	HMGB1 mediates splenomega	J Intern Med	2013
23463879	eng	Kovacic V, Ljuti	Spleen rupture associated wit	Blood Purif	2013
23421883	eng	Apa H, Devrim I,	Factors affecting Brucella spp	Vector Borne Zoo	2013
23121673	eng	Litzman J, Nech	Chronic immune activation in	Clin Exp Immunol	2012
22992554	eng	Giuliano S, Rubi	Streptococcus anginosus gro	Infez Med	2012
22560926	eng	Satoh F, Seto Y	Fatal Staphylococcus aureus	Leg Med (Tokyo)	2012
22143787	eng	Vuyyuru R, Liu I	Characteristics of Borrelia he	Proc Natl Acad Sc	2011
21893423	eng	Logan LK, Jacol	A multicenter retrospective s	Int J Infect Dis	2011
20721485	eng	Lambertucci JR	Acute schistosomiasis manso	Mem Inst Oswaldo	2010
20408849	eng	Novelli EM, Hittr	Clinical predictors of severe n	Br J Haematol	2010
19024219	eng	Cacopardo B, Sa	Description of a rare case of	Eur Rev Med Phar	2008
18387752	fre	Zribi M, Ammari	[Clinical manifestations, comp	Pathol Biol (Paris)	2009
18094131	eng	Lydy SL, Ereme	Isolation and characterization	J Clin Microbiol	2008
17928968	ger	Linde B, Oelzne	[Fulminate liver failure in a 39	Med Klin (Munich)	2007
17554119	eng	Eremeeva ME, C	Bacteremia, fever, and spleno	N Engl J Med	2007
17475847	eng	Cunningham AF	Salmonella induces a switche	J Immunol	2007
16511385	eng	Brent AJ, Ound	Salmonella bacteremia in Ken	Pediatr Infect Dis	2006
16228056	eng	Fallatah SM, Od	Human brucellosis in Norther	Saudi Med J	2005
15313681	eng	Omanwar S, Riz	A rabbit model of non-cirrhot	Hepatobiliary Panc	2004
15308422	eng	Nomura R, Naka	Contribution of glucan-bindin	Arch Oral Biol	2004
15304000	eng	Peters RP, Zijlst	A prospective study of bloods	Trop Med Int Healt	2004
12884816	jpn	Katayama T, Kar	[Fatal septic shock and rhabd	Rinsho Ketsueki	2003
12757224	eng	Shwe TN, Nyein	Blood culture isolates from ch	Southeast Asian J	2002
12243791	eng	Shrestha S, Shr	Bacterial peritonitis in hepatic	Hepatol Res	2002
11902310	eng	Sakhalkar VS, R	Hemophagocytosis and granul	J Pediatr Hematol	2001
11876755	eng	Messias-Reaso	Complement activation in infe	Clin Exp Immunol	2002
11709134	spa	Ramón Maestre	[Bacteremia caused by Campy	Enferm Infecc Mic	2001
11597152	eng	Sirmatel F, Balci	A case of Salmonella paratyph	J Infect	2001
11300950	eng	Sotomayor EA,	Pathologic quiz case. A huma	Arch Pathol Lab M	2001
10996126	eng	Lambertucci JR	Schistosoma mansoni: assess	Acta Trop	2000
10783021	eng	Walsh AL, Phiri	Bacteremia in febrile Malawian	Pediatr Infect Dis	2000

10353865	eng	Archibald LK, Mc	Fever and human immunodefi	J Infect Dis	1999
9916105	eng	MacFarlane AS,	In vivo blockage of nitric oxide	Infect Immun	1999
9833784	eng	Pantongrag-Brc	Frequency of abdominal CT fir	Clin Radiol	1998
9524640	eng	Jacobs G, Calve	Neutropenia and thrombocyto	J Am Vet Med Ass	1998
9378064	ger	Hungerland E, Ei	[Clostridium sordellii sepsis wi	Dtsch Med Woche	1997
9046942	eng	Das M, Badley A	Infective endocarditis caused	Annu Rev Med	1997
8912878	eng	Smith SR, Term	Administration of interleukin-	Cell Immunol	1996
8730142	eng	Krishnaswamy G	Resolution of the neutropeni	J Rheumatol	1996
8712515	eng	Breitschwerdt E	Myocarditis in mice and guine	Am J Vet Res	1996
8579089	eng	Chassagne P, P	Is presentation of bacteremia	Am J Med	1996
8584358	eng	Sirisanthana V,	Disseminated Penicillium marr	Pediatr Infect Dis	1995
8565612	eng	Carroll J, Thaler	Generalized pustular eruption	Cutis	1995
7984081	eng	Arber N, Pras E,	Pacemaker endocarditis. Repo	Medicine (Baltimor	1994
8505767	eng	Akpede GO, Syk	Malaria with bacteraemia in ac	J Trop Med Hyg	1993
1495126	eng	Chandra R, Srini	Multidrug resistant enteric fe	J Trop Med Hyg	1992
2108081	eng	Ramdani, Dawkir	Pasteurella multocida infectio	Immunol Cell Biol	1990
2663979	eng	Heimberger TS,	Infections of prosthetic heart	Infect Dis Clin Nor	1989
3737076	eng	Prichard JG, Lo	Streptococcus milleri pyomyc	Obstet Gynecol	1986
4071169	eng	Alvarez-Elcoro	Community-acquired febrile ill	South Med J	1985
7097408	eng	Topley JM, Cupi	Pneumococcal and other infe	J Pediatr	1982
7056685	eng	Brumbaugh GW,	Myelomonocytic myeloprolifer	J Am Vet Med Ass	1982
14226112	eng	EDWARDS AM,	PRIMARY MACROGLOBULIN	Can Med Assoc J	1964
13228339	fre	LACROIX AC, J	[Polyarticular syndrome with	Alger Medicale	1954

Volume	Pages	Pub. Type
39	6	Case Repor
20(1)	915	Journal Art
89(1)		Journal Art
11	1277	Journal Art
33(9)	10005-100	Journal Art
38(7)	1261-1268	Evaluation S
41(7)	457	Case Repor
18	859-864	Case Repor
12(5)	055006	Journal Art
177(5)	1439-1441	Case Repor
144(4)	557-562	Journal Art
34(26)	2907-2910	Journal Art
15	445	Case Repor
10(4)	e0122139	Journal Art
38(3-4)	239-41	Case Repor
71(3)	317-24	Case Repor
12 Suppl 1(	s141-3	Case Repor
274(4)	381-90	Journal Art
35(1-3)	177-80	Case Repor
13(3)	176-80	Journal Art
170(3)	321-32	Journal Art
20(3)	145-54	Case Repor
14(5)	246-8	Case Repor
108(51)	20707-12	Journal Art
15(12)	e812-7	Journal Art
105(4)	422-35	Journal Art
149(5)	711-21	Journal Art
12(5)	331-4	Case Repor
57(5)	349-52	Journal Art
46(2)	627-37	Case Repor
102(10)	846-51	Case Repor
356(23)	2381-7	Case Repor
178(10)	6200-7	Journal Art
25(3)	230-6	Journal Art
26(10)	1562-6	Comparativ
3(3)	417-22	Journal Art
49(10)	783-8	Journal Art
9(8)	928-34	Journal Art
44(6)	381-5	Case Repor
33(4)	764-71	Journal Art
24(1)	42	Journal Art
23(9)	623-5	Case Repor
127(2)	310-5	Journal Art
19(9)	457-8	Case Repor
43(1)	19	Case Repor
125(5)	697-8	Case Repor
77(1)	101-9	Journal Art
19(4)	312-8	Journal Art

180(1)	87-92	Journal Art
67(2)	891-8	Journal Art
53(11)	816-9	Journal Art
212(5)	681-4	Case Repoı
122(42)	1281-4	Case Repoı
48	25-33	Journal Art
173(2)	207-14	Journal Art
23(4)	763-5	Case Repoı
57(4)	505-11	Comparativ
100(1)	65-70	Comparativ
14(11)	935-40	Journal Art
56(5)	276-8	Case Repoı
73(6)	299-305	Journal Art
96(3)	146-50	Journal Art
95(4)	284-7	Journal Art
68 (Pt 1)	57-61	Comparativ
3(2)	221-45	Journal Art
68(3 Suppl)	46S-49S	Case Repoı
78(12)	1431-4	Journal Art
101(2)	176-9	Comparativ
180(3)	313-6	Case Repoı
91(25)	1300-6	Journal Art
58(10)	747-9	Journal Art

Abstract
Tatumella ptyseos septicaemia in humans is yet to be reported in Nigeria with very few cases r
BACKGROUND: The spleen contains immune cells and exhibits a pattern of infarction different
Immunocompromised patients are more susceptible to recurrent nontyphoidal Salmonella (NT
Non-typhoidal Salmonella (NTS) causes gastrointestinal infection, which is commonly self-limit
The aim of the present study was to examine whether the immune-modulatory bacteria Lacto
Brucellosis in pregnant women is reported to be associated with obstetric complications (OCs)
ts; Journal Article
BACKGROUND In Primary Myelofibrosis (PMF; a clonal disorder arising from the neoplastic tran
Biomaterial-associated Pseudomonas aeruginosa biofilm infections constitute a cascade of ho
Mastocytosis is a clinically heterogenous disease characterized by mast cell hyperplasia in skin
Endotoxaemia has been described in cases of Human African trypanosomiasis (HAT), but it is u
Among more than 2500 nontyphoidal Salmonella enterica (NTS) serovars, S. enterica serovar
BACKGROUND: Visceral leishmaniasis (VL), i.e., infection with Leishmania sp. associated with h
Differentiation of infectious causes in severely ill children is essential but challenging in sub- S
BACKGROUND: We report here a case of a woman affected by fever, weight loss, splenomegal
INTRODUCTION: Rhodococcus (R) equi is an opportunistic, uncommon human pathogen that
ts; Journal Article
BACKGROUND: More than 500,000 hospitalized patients survive severe sepsis annually in the
We present an uremic patient on chronic hemodialysis with splenic septic emboli associated wi
Brucella infections have a wide spectrum of symptoms especially in children, making the diagno
Common variable immunodeficiency (CVID), the most frequent symptomatic immunoglobulin pri
Streptococcus anginosus group is widely known for its ability to cause invasive pyogenic infec
Familial neglect was suspected when an older deceased female was found to have large decubi
Rodents are natural reservoirs for a variety of species of Borrelia that cause relapsing fever (F
OBJECTIVES: To determine risk factors in children for the acquisition of Brucella, clinical pres
Acute schistosomiasis is a systemic hypersensitivity reaction against the migrating schistoson
Severe malarial anaemia (SMA) is a common complication of Plasmodium falciparum infections,
We report on a rare case of splenic abscess due to spontaneous Escherichia coli bacteremia i
The aim of this study was to evaluate the clinical, laboratory findings and therapeutic features
Carrion's disease is typically biphasic with acute febrile illness characterized by bacteremia and
BACKGROUND: Fulminate liver insufficiency can have many causes and is a challenge for diffe
Bartonella species cause serious human infections globally, including bacillary angiomatosis, Or
T-dependent Ab responses are characterized by parallel extrafollicular plasmablast growth and
BACKGROUND: Nontyphoidal Salmonella spp. are among the leading causes of childhood bacte
OBJECTIVES: Analysis of the clinical features, laboratory findings, treatment given and complic
BACKGROUND: Non-cirrhotic portal hypertension is a common cause of portal hypertension
Our previous analysis of major cell surface proteins of Streptococcus mutans isolated from th
OBJECTIVE: To determine the contribution of a blood culture service to the diagnosis of feve
We report on a 58-year-old male diagnosed as having primary myelofibrosis with thrombocytop
A one year study (August 1998-July 1999) of bacteremia in febrile children was carried out in
Hepatic IVC disease (HVD), a disease caused by complete obstruction or stenosis of inferior ve
Persistent fever with pancytopenia and hepatomegaly with negative blood cultures and no obvi
In an infectious process complement activation is necessary for a proper immune and inflamm
ts; Letter
ts; Letter
ts; Journal Article
The literature on the assessment of morbidity due to Schistosoma mansoni infection is update
BACKGROUND: There are no published data for the incidence or etiology of childhood bacter

To determine the etiology of bloodstream infections (BSIs) in hospitalized patients >/=15 year
Our laboratory has previously shown that after immunization with a strain of Salmonella typhim
BACKGROUND: Use of blood culture studies for early diagnosis of Mycobacterium avium comp
Three dogs became lethargic and had poor appetites within 2 months after anticonvulsant trea
HISTORY AND CLINICAL FINDINGS: A 37-year-old chronic alcoholic had increasingly frequer
The HACEK group of fastidious gram-negative organisms is a recognized but unusual cause of
Several laboratories have described the protective effects of interleukin-10 (IL-10) in mouse
Felty's syndrome is characterized by neutropenia, splenomegaly, and recurrent infection in pat
OBJECTIVE: To characterize the pathogenic potential of a unique Borrelia isolate obtained fro
OBJECTIVE: To compare the presentation of bacteremia in young and elderly patients. PATIEN
Disseminated infection with the fungus Penicillium marneffei is one of the most common oppo
A 67-year-old man presented with a high fever and a generalized rash. His extended hospital s
We conducted a retrospective study to characterize the clinical course, microbiologic spectru
Data were collected on 642 preschool children who presented consecutively to casualty with
Multidrug resistant typhoid fever (MDRT) is becoming an alarming public health problem in and a
Haemorrhagic septicaemia (HS) is an infectious disease of cattle and buffalo caused by particul
Prosthetic valve endocarditis may be considered present when two fo the following criteria are
A 37-year-old woman with clinically occult, abscessed uterine myomas presented with fever, a
We prospectively studied 40 patients with prosthetic heart valves and community-acquired feb
The pattern of infection was compared in 139 children with sickle cell-hemoglobin C (SC) disea
Myelomonocytic myeloproliferative disease in a horse was diagnosed on the basis of hematolog
Clinical features presented by a patient with primary macroglobulinemia over a four-year perioc
:icle

Memo	MeSH
eported woı	Bacteremia/*diagnosis; Cough/etiology; Female; Fever/etiology; Gammaproteobacteria/*isola
: from otheı	Adult; Aged; Bacteremia/*epidemiology; Blood Culture; Comorbidity; Cytomegalovirus/*isolatic
S) bacterem	Anemia/diagnosis/*etiology; Animals; Bone Marrow/pathology; CD4 Lymphocyte Count; CD4-
:ing in healtl	Animals; *Bacterial Proteins; Female; HeLa Cells; Humans; Mice; Mice, Inbred BALB C; RAW 26
bacillus ferr	Acetylcholine/pharmacology; Animals; Aorta/drug effects; Bacterial Translocation; Bifidobacte
), and adequ	Abortion, Spontaneous/microbiology; Adolescent; Adult; Bacteremia/epidemiology; Brucella/c
	Abdominal Abscess/diagnostic imaging/*etiology/microbiology/surgery; Aged; Bacteremia/*c
ısformation	Bacteremia/*complications/microbiology; Epidural Abscess/complications/*microbiology; Hu
st immune ı	Animals; Animals, Genetically Modified; Anti-Bacterial Agents/pharmacology; Biocompatible Ma
, bone marr	Ascites/diagnostic imaging; Fatal Outcome; Female; Hepatomegaly/diagnostic imaging; Human:
nclear if thi:	Adolescent; Adult; Child; Cytokines/genetics/metabolism; Endotoxemia/*etiology/*pathology
Typhimuriur	Animals; Bacteremia/epidemiology/prevention & control; Biomedical Research/trends; Clinica
igh fever, w	Adult; Anti-Bacterial Agents/therapeutic use; Antibodies, Helminth/blood; Bacteremia/preve
Saharan Afri	Adolescent; Anthropometry; Bacteremia/*epidemiology/pathology; Child; Child, Preschool; Co
y, and leuco	Acute Kidney Injury/etiology/*therapy; Anticoagulants/therapeutic use; Antiprotozoal Agen
causes maiı	Actinomycetales Infections/*complications/drug therapy/microbiology; Adult; Anti-Bacterial
	Anemia, Hemolytic/diagnosis; Bacteremia/complications/microbiology; Child, Preschool; Citro
USA. Rece	Animals; Antigens, Ly/*immunology; Bacteremia/*immunology; CD11b Antigen/*immunology;
ith active in	Bacteroides Infections/complications/diagnostic imaging/microbiology/*pathology; Embolism,
ısis a compli	Adolescent; Alanine Transaminase/blood; Aspartate Aminotransferases/blood; Bacteremia/*c
imary immur	Adolescent; Adult; Aged; B-Lymphocytes/immunology; Bronchiectasis/blood; C-Reactive Pro
tions. There	Anti-Bacterial Agents/*therapeutic use; Bacteremia/diagnosis/*microbiology/therapy; Brain
tus ulcers a	Aged; Autopsy; Bacteremia/*etiology/microbiology; Elder Abuse/*legislation & jurisprudence;
RF) in humaı	Animals; Antigens/metabolism; Antigens, CD34/biosynthesis; Borrelia/*metabolism; Borrelia I
entation, tr	Adolescent; Animals; Anti-Bacterial Agents/*therapeutic use; Bacteremia/diagnosis/drug th
ıula and egg	Acute Disease; Animals; Female; Humans; Male; *Schistosomiasis mansoni/complications/diag
resulting in	Anemia/diagnosis/genetics/*parasitology; Child, Preschool; Endemic Diseases; Female; Huma
n a 49-yeaı	Abscess/complications/*microbiology; Anti-Bacterial Agents/therapeutic use; Bacteremia/c
of patients '	Adolescent; Adult; Aged; Aged, 80 and over; Anti-Bacterial Agents/pharmacology/therapeutic
severe hen	Adult; Antibodies, Bacterial/blood; Bacterial Proteins/analysis; Bartonella Infections/*microbi
rential diagn	Administration, Oral; Adult; Arthralgia/*etiology; Biopsy; Diagnosis, Differential; Dipeptides/adm
oya fever, tı	Adult; Anemia/etiology; Bacteremia/*microbiology; Bartonella/genetics/*isolation & purificat
germinal ce	Animals; Antibodies, Bacterial/*biosynthesis/physiology; CD40 Ligand/deficiency/genetics; E:
remia in sul	Anemia/complications; Bacteremia/complications/*epidemiology/microbiology/mortality; Chil
:ations seer	Adolescent; Adult; Age Distribution; Aged; Anti-Bacterial Agents/*therapeutic use; Brucella/
in developin	Animals; Blood Pressure; Catheters, Indwelling; *Disease Models, Animal; Endotoxemia/*comp
e blood of a	Animals; Bacteremia/*genetics/metabolism; Bacterial Adhesion/genetics; Bacterial Proteins,
r in a resou	Adolescent; Adult; Aged; Bacteremia/complications; Diagnosis, Differential; Female; Fever/*m
enia, who di	Blood Platelets/*microbiology; Blood Preservation; *Drug Contamination; Fatal Outcome; Hur
the Medica	Age Distribution; Bacteremia/blood/complications/*epidemiology/*microbiology; Bacteriologi
ena cava (IVC) near cava-atrial junction is endemic in Nepal. It is a chronic disease characterized by uppe	
ious focus c	Bacteremia/complications/microbiology; Bone Marrow/microbiology/*pathology; Child, Presc
atory respoı	Adolescent; Adult; Aged; Antigen-Antibody Complex/blood; Bacteremia/complications; Centra
	Adult; Anemia, Hemolytic, Autoimmune/*complications; Bacteremia/complications/*microbio
	Acute Kidney Injury/*microbiology; Adult; Disseminated Intravascular Coagulation/*microbiol
	AIDS-Related Opportunistic Infections/complications/*diagnosis/microbiology; Adult; Bacter
ed. Imaging t	Brazil/epidemiology; Central Nervous System Diseases/diagnosis/parasitology; *Communicabl
emia in Malaı	Adolescent; Africa/epidemiology; Anti-Bacterial Agents/administration & dosage/pharmacolo

s old in Thai	Adolescent; Adult; Aged; Aged, 80 and over; Bacteremia/complications/*epidemiology/mortal
urium, SL32	Animals; Enzyme Inhibitors/pharmacology; Female; Guanidines/*pharmacology; Immune Tolera
lex (MAC) in	AIDS–Related Opportunistic Infections/*diagnostic imaging; Adult; Bacteremia/*diagnostic im
atment was	Anemia/chemically induced/veterinary; Animals; Anticonvulsants/*adverse effects/therapeu
it haemater	Adult; Bacteremia/*complications/*microbiology; Clostridium Infections/*complications/*mi
infective er	Adult; Aged; Bacteremia/diagnosis/mortality/therapy; Bacteriological Techniques; Combined M
models of le	Animals; Antibodies, Monoclonal/administration & dosage/immunology; Cytokines/biosynthesi
tients with r	Aged; Bacteremia/complications; Felty Syndrome/blood/complications/*drug therapy; Follow
m a dog fro	Animals; Bacteremia/pathology/*physiopathology; Borrelia/isolation & purification/*pathogen
NTS AND MI	Aged; Aged, 80 and over; *Aging; Bacteremia/blood/*diagnosis/microbiology/physiopathology
rtunistic inf	AIDS–Related Opportunistic Infections/*diagnosis/drug therapy/microbiology; Antifungal Age
tay was cha	Aged; Angiotensin–Converting Enzyme Inhibitors/*adverse effects; Antihypertensive Agents/
im, and risk	Aged; Anti–Bacterial Agents/therapeutic use; Bacteremia/epidemiology/etiology; Endocarditi
fever and n	Age Factors; Anemia; Bacteremia/*complications/epidemiology; Child, Preschool; Enterobact
iround Ponc	Anti–Bacterial Agents/therapeutic use; Child; Child, Preschool; Ciprofloxacin/therapeutic use
ar serotype	Animals; Buffaloes/immunology/*microbiology; Cattle; Cattle Diseases/immunology/*microbio
e met: (1) tw	Bacterial Infections/*etiology; Endocarditis, Bacterial/*etiology; Heart Valve Prosthesis/*adv
anemia, sple	Abscess/*diagnosis/etiology; Adult; Diagnosis, Differential; Endocarditis, Bacterial/*diagnosis;
orile illness.	Adult; Bioprosthesis; Endocarditis, Bacterial/*etiology; Female; Fever/*etiology; *Heart Valve
ase and in 2	Bacterial Infections/complications/*epidemiology/mortality; Child; Child, Preschool; Hemoglob
gic, enzymat	Animals; Horse Diseases/*blood; Horses; Myeloproliferative Disorders/pathology/*veterinary
d included ca	*Blood Protein Electrophoresis; *Bone Marrow Cells; *Death; *Geriatrics; Humans; *Immuno
	Bacteremia/*microbiology; *Bacteriology; *Fever; Humans; *Joint Diseases; Penicillins/*ther

ation & purification; Gram-Negative Bacterial Infections/*diagnosis; Humans; Infant; Nigeria; Tertiary Care
on & purification; Cytomegalovirus Infections/*epidemiology/virology; Endocarditis/*epidemiology/microbi
-Positive T-Lymphocytes/*immunology/metabolism; Disease Models, Animal; Disease Susceptibility; Immu
64.7 Cells; Salmonella Infections/*prevention & control; Salmonella Vaccines/*immunology; Salmonella ty
erium/isolation & purification; Cytokines/blood; Disease Models, Animal; Dysbiosis/etiology/microbiology/*
drug effects/isolation & purification; Brucellosis/*complications/*epidemiology; Cross-Sectional Studies;
complications; Clostridium/isolation & purification; Clostridium Infections/*complications; Humans; Male; Sh
mans; Male; Middle Aged; Primary Myelofibrosis/*complications; Salmonella/isolation & purification; Salmon
aterials/chemistry; *Biofilms; Disease Models, Animal; Female; Immune System; Immunohistochemistry; Infl
s; Infant, Newborn; Male; Mastocytosis, Cutaneous/*cerebrospinal fluid/congenital/diagnostic imaging; Ma
; Female; Humans; Male; Trypanosomiasis, African/*blood/cerebrospinal fluid/*pathology; Young Adult
al Trials as Topic; Humans; O Antigens/immunology; Pathogen-Associated Molecular Pattern Molecules/im
ntion & control; DNA Primers/metabolism; DNA, Protozoan/analysis; Humans; Immunocompromised Host;
infection/*epidemiology/microbiology; Cough/pathology; Dehydration/pathology; Ghana; Humans; Malaria,
ts/therapeutic use; Bacteremia/diagnosis; Bone Marrow Examination; Combined Modality Therapy; Coryne
Agents/therapeutic use; Antineoplastic Agents/therapeutic use; Diagnosis, Differential; Female; Follow-U
bacter freundii; Diagnosis, Differential; Enterobacter cloacae; Enterobacteriaceae Infections/complication
Cecum/injuries; Disease Models, Animal; HMGB1 Protein/*physiology; Humans; Inflammation/immunology
/complications/diagnostic imaging/*pathology; Endocarditis, Bacterial/complications/diagnostic imaging/m
diagnosis/epidemiology/microbiology; Blood Specimen Collection; Bone Marrow/microbiology; Brucella/*is
otein/immunology/metabolism; Common Variable Immunodeficiency/*blood/*immunology; Endotoxemia/b
Abscess/microbiology/therapy; Combined Modality Therapy; Empyema, Pleural/microbiology/therapy; Hep
Felty Syndrome/*complications; Female; Forensic Pathology; Humans; Neutropenia/complications; Press
nfections/*metabolism/microbiology; Flow Cytometry/methods; Hematopoietic Stem Cell Transplantation
erapy/*epidemiology/microbiology; Brucella/drug effects/*isolation & purification; Brucellosis/diagnosis/
gnosis/drug therapy/transmission; Schistosomicides/therapeutic use
ns; Infant; Kenya/epidemiology; Malaria, Falciparum/*complications/epidemiology/genetics/transmission; I
complications/microbiology; Blood Cell Count; Ceftazidime/therapeutic use; Escherichia coli Infections/co
use; Arthritis, Infectious/etiology; Bacteremia/diagnosis/drug therapy/epidemiology/microbiology; Bruc
ology/pathology/physiopathology; Bartonella bacilliformis/*isolation & purification; Biopsy; Blood/immunolo
inistration & dosage; Dose-Response Relationship, Drug; Drug Administration Schedule; Drug Therapy, Co
ion; Bartonella Infections/*microbiology; DNA, Bacterial/analysis; Electrophoresis; Female; Fever/microbi
xtracellular Space/*immunology/*microbiology; Germinal Center/*immunology/microbiology/*pathology;
ld; Child, Preschool; Female; HIV Infections/complications; Humans; Infant; Kenya/epidemiology; Malaria, Fa
*classification/isolation & purification; Brucellosis/*diagnosis/drug therapy/*epidemiology; Cohort Studie
lications/pathology/*physiopathology; Escherichia coli; Female; Hypertension, Portal/*etiology/pathology
/*genetics/metabolism; Body Weight/physiology; Carrier Proteins/*genetics/metabolism; Genes, Bacteri
icrobiology; HIV Infections/complications; Humans; Malawi; Male; Middle Aged; Parasitemia/complications; F
mans; Male; Middle Aged; Platelet Transfusion/*adverse effects; Pneumococcal Infections/*etiology/micr
cal Techniques; Bradycardia/microbiology; Child; Child, Preschool; Cluster Analysis; Drug Resistance, Bact
r abdominal pain, hepatomegaly, splenomegaly and dilated superficial veins in the body trunk. Ascites comn
hool; Diagnosis, Differential; Down Syndrome/*complications/pathology; Epstein-Barr Virus Infections/*c
al Nervous System Diseases/etiology; *Complement Activation; Complement C3d/analysis; Complement M
logy; Campylobacter Infections/complications/*microbiology; Campylobacter jejuni/drug effects/*isolatio
ogy; Fatal Outcome; Hemorrhagic Septicemia/*microbiology; Hepatomegaly/microbiology; Humans; Male; M
emia/complications/*diagnosis/microbiology; HIV Infections/*complications; Humans; Male; Mycobacteri
le Disease Control; Glomerulonephritis/complications; Hepatitis B/complications; Humans; Hypertension, F
gy; Bacteremia/diagnosis/drug therapy/*epidemiology/microbiology; Child; Child, Preschool; Comorbidity; D

ity; Cohort Studies; Cryptococcus neoformans/isolation & purification; Developing Countries; Female; Fev
ance/drug effects; Macrophages/*immunology; Mice; Mice, Inbred C3H; Neutrophils/*immunology; Nitrate
naging; Female; Humans; Lymphatic Diseases/diagnostic imaging; Male; Mesentery; Middle Aged; Mycobacte
ıtic use; Bacteremia/etiology/veterinary; Blood Cell Count/veterinary; Dog Diseases/*chemically induced
crobiology; Fatal Outcome; *Hemolysis; Humans; Male
Modality Therapy; Echocardiography; Endocarditis, Bacterial/*diagnosis/mortality/therapy; Female; Gram-N
s; Endotoxemia/prevention & control; Humans; Immunization; Interleukin-10/immunology/*pharmacology
-Up Studies; Granulocyte Colony-Stimulating Factor/*administration & dosage/therapeutic use; Herpes
nicity; Borrelia Infections/pathology/*physiopathology; Dogs/*microbiology; Florida; Guinea Pigs; Humans; I
; Bacteria/isolation & purification; Blood Sedimentation; Case-Control Studies; Female; Fever/physiopath
ents/therapeutic use; Child; Child, Preschool; Dermatomycoses/*diagnosis/drug therapy/microbiology; Fe
/*adverse effects; Bacteremia/microbiology; Captopril/*adverse effects; Drug Eruptions/*etiology; Huma
s, Bacterial/*epidemiology/etiology/microbiology; Enterococcus faecalis/isolation & purification; Female;
eriaceae Infections/complications; Fever; Hepatomegaly; Humans; Infant; Malaria, Falciparum/*complicatio
; *Drug Resistance, Microbial; Humans; Infant; Microbial Sensitivity Tests; Typhoid Fever/complications/di
ology/pathology; Disease Models, Animal; Female; Hemorrhagic Septicemia/immunology/*microbiology/pat
verse effects; Humans; Pacemaker, Artificial/*adverse effects
Female; Fistula/etiology; Humans; Leiomyoma/*complications; Sepsis/etiology; Streptococcal Infections/
Prosthesis; Humans; Male; Middle Aged; *Postoperative Complications; Prospective Studies; Time Factors
oin C Disease/*complications/mortality; Humans; Infant; Infant, Newborn; Jamaica; Pneumococcal Infectic

electrophoresis; *Liver; *Lymph Nodes; *Lymphatic Diseases; *Macroglobulins; *Mesenteric Vascular Occ
rapeutic use; Sepsis/*microbiology; *Sodium Salicylate; Splenomegaly/*complications; *Streptococcal In

Centers
ology; Epstein-Barr Virus Infections/*epidemiology/virology; Female; Herpesvirus 4, Human/*isolation & p
nity, Cellular; *Immunocompromised Host; Mice; Myelopoiesis/*immunology; Salmonella Infections/compli
yphimurium/*immunology; Vaccines, Attenuated/immunology
therapy; Endotoxemia/etiology/prevention & control; Female; Gastrointestinal Microbiome; Hypertension,
Female; Fever/epidemiology/microbiology; Humans; Infant, Newborn; Middle Aged; Pregnancy; Pregnancy
hock, Septic/etiology; Splenectomy; Splenic Diseases/diagnostic imaging/*etiology/microbiology/surgery
ella Infections/*diagnosis; Spinal Cord Compression/complications/*etiology
lammation; Interferon-beta/metabolism; Magnesium/*chemistry; Materials Testing; Mice; Mice, Inbred BAL
stocytosis, Systemic/*congenital/diagnostic imaging; Pregnancy; Splenomegaly/diagnostic imaging; Ultras

mmunology; Salmonella Infections/epidemiology/*prevention & control; Salmonella Vaccines/*therapeuti
Leishmaniasis, Visceral/*diagnosis/genetics/immunology; Male; Real-Time Polymerase Chain Reaction; F
Falciparum/*epidemiology/*microbiology; Odds Ratio; Predictive Value of Tests; Prevalence; Regression
bacterium Infections/diagnosis; Critical Care/methods; Cytokines/blood; Delayed Diagnosis; Diagnostic Er
Jp Studies; Hodgkin Disease/*complications/diagnosis/drug therapy; Humans; Magnetic Resonance Imagi
s/microbiology; Female; Hemoglobinuria, Paroxysmal/*etiology; Humans; Leishmania infantum/isolation &
; Ligation; Male; Mice; Mice, Inbred BALB C; Monocytes/*immunology; Punctures/adverse effects; Spleer
nicrobiology/*pathology; Fatal Outcome; Female; Humans; Middle Aged; Mitral Valve/microbiology/patholog
solation & purification; Brucellosis/*diagnosis/epidemiology/microbiology; C-Reactive Protein/analysis; Ch
lood/*immunology; Female; Granuloma/blood; Humans; IgA Deficiency/blood/immunology; Interleukin-2 F
patomegaly/microbiology; Humans; Liver Abscess/microbiology/therapy; Lung Abscess/microbiology/ther
sure Ulcer/*etiology/microbiology; Staphylococcal Infections/*etiology; Staphylococcus aureus
; Hematopoietic Stem Cells/*cytology; Humans; Lymphocytes/cytology; Mice; Mice, Inbred C57BL; Mice,
drug therapy/*epidemiology/microbiology; Chicago/epidemiology; Child; Child, Preschool; Drug Therapy, Co

Male; Pallor/parasitology; Physical Examination/methods; Platelet Count; Reticulocyte Count; Risk Factor
omplications/*microbiology; Humans; Liver Cirrhosis, Alcoholic/*complications; Male; Middle Aged; Splenic
ella abortus/drug effects/*isolation & purification; Brucellosis/complications/diagnosis/drug therapy/*ep
gy/microbiology; Blotting, Western; DNA, Bacterial/chemistry/genetics; DNA, Ribosomal Spacer/chemist
mbination; Female; Fever of Unknown Origin/*etiology; Hepatomegaly/etiology; Humans; Immunosuppress
ology; Humans; Peru; Phylogeny; Polymerase Chain Reaction; Splenomegaly/microbiology; Travel
Immunoglobulin G/biosynthesis; Mice; Mice, Inbred C57BL; Mice, Knockout; Mice, Transgenic; Salmonella
alciparum/complications; Male; Malnutrition/complications; Risk Factors; Salmonella/classification/isolatio
s; Female; Follow-Up Studies; Humans; Incidence; Male; Middle Aged; Retrospective Studies; Risk Assessn
/*physiopathology; Injections, Intravenous; Male; Organ Size; *Rabbits; Spleen/blood supply; Splenomegal
al/genetics; Humans; Lectins; Leukocytes, Mononuclear/physiology; Male; Mutation/genetics; N-Acetyln
Prospective Studies; Sepsis/*complications; Splenomegaly/microbiology; Tuberculosis/complications
robiology/pathology; Primary Myelofibrosis/therapy; Rhabdomyolysis/*etiology/microbiology/pathology; Sh
erial; Female; Fever/*microbiology; Hepatomegaly/microbiology; Hospitals, Pediatric/*statistics & numeric
nonly, with high protein content is a feature of acute and subacute stages and during acute exacerbation
complications/diagnosis/pathology; Granuloma/etiology/*pathology; Hepatomegaly/etiology/pathology; His
Membrane Attack Complex/analysis; Endocarditis, Bacterial/blood/complications/*immunology/mortality; F
n & purification; Clarithromycin/therapeutic use; Drug Resistance; Drug Therapy, Combination/therapeuti
Multiple Organ Failure/*microbiology; Paratyphoid Fever/*complications; Rhabdomyolysis/*microbiology; S
um avium Complex/isolation & purification; Mycobacterium avium-intracellulare Infection/complications/*
Pulmonary/complications/diagnosis; Intestinal Diseases/diagnosis/parasitology; Liver Diseases/diagnosis/
Developing Countries; Female; Fever of Unknown Origin/*epidemiology; Gram-Negative Bacterial Infection

ver/complications/*epidemiology/mortality; Fungemia/complications/*epidemiology/mortality; HIV Infecti
s/metabolism; Nitric Oxide/antagonists & inhibitors/*physiology; Nitric Oxide Synthase/antagonists & inh
rium avium-intracellulare Infection/*diagnostic imaging; *Radiography, Abdominal; Retroperitoneal Space;
d/drug therapy; Dogs; Female; Male; Neutropenia/chemically induced/complications/*veterinary; Phenoba

legative Bacterial Infections/*diagnosis/mortality/therapy; Heart Valve Prosthesis; Humans; Male; Microbi
r; Lipopolysaccharides/antagonists & inhibitors/toxicity; Male; Mice; Mice, Inbred C57BL; Mice, Inbred DBA
Zoster/complications; Humans; Leukocyte Count; Male; Neutropenia/blood/complications/*drug therapy
Hyperplasia; Lymphoid Tissue/pathology; Mice; Myocarditis/*microbiology/pathology/physiopathology; Myo
ology; Humans; Hypothermia/physiopathology; Leukopenia/physiopathology; Logistic Models; Lymphopenia/
male; Fungemia/*diagnosis/drug therapy/microbiology; HIV Infections/congenital; Humans; Infant; Male; F
ns; Hyperpigmentation/chemically induced; Male; Mycosis Fungoides/*chemically induced; Skin Ulcer/che
Follow-Up Studies; Humans; Israel/epidemiology; Klebsiella/isolation & purification; Male; Middle Aged; Pace
ons; Prevalence; Splenomegaly; Staphylococcal Infections/complications
iagnosis/*drug therapy
hology/*veterinary; Immunity, Innate; Kinetics; Mice; Mice, Inbred BALB C; Pasteurella/*pathogenicity; P

/*diagnosis/etiology; Suppuration; Uterine Diseases/*diagnosis/etiology; Uterine Neoplasms/complicatior
;
ons/complications/*epidemiology

clusion; *Mononuclear Phagocyte System; *Necrosis; *Portal System; *Radiography; *Sepsis; *Spleen; *S
fections; *Viridans Streptococci

ɔurification; Hospitals, University; Humans; Malaria, Vivax/*epidemiology/parasitology; Male; Middle Aged; Ori
cations/diagnosis/*immunology/*microbiology; Salmonella typhimurium/immunology; Severity of Illness Ir

/etiology/prevention & control; Kidney/pathology; *Lactobacillus fermentum; Lupus Erythematosus, Syst
Complications, Infectious/epidemiology/*microbiology; Retrospective Studies; Splenomegaly/epidemiolog
; Splenic Rupture/etiology/surgery; Splenomegaly/etiology/surgery; *Tomography, X-Ray Computed

.B C; Microscopy, Electron; Microscopy, Electron, Scanning; Microscopy, Electron, Transmission; *Prosth
onography, Prenatal

ic use; Salmonella enteritidis; Salmonella typhimurium; Vaccines, Subunit/therapeutic use
Remission, Spontaneous
Analysis; Rural Population; Salmonella Infections/*epidemiology/pathology; Sensitivity and Specificity; Soc
rors; False Positive Reactions; Female; Hemofiltration/*instrumentation; Humans; Leishmaniasis/blood/*
ng; Rhodococcus equi/*isolation & purification; Tomography, X-Ray Computed
ɔurification; Leishmaniasis, Visceral/*complications/diagnosis/parasitology; Parasitemia/*complications/d
n/immunology; Splenomegaly/*immunology
y; *Renal Dialysis; Spleen/diagnostic imaging/pathology; Splenic Rupture/complications/diagnostic imagin
nild; Child, Preschool; Fever; Hemoglobins/analysis; Hepatomegaly; Humans; Infant; Male; Retrospective Stu
Receptor alpha Subunit/*blood/immunology; Lipopolysaccharide Receptors/*blood/immunology; Lipopolys
rapy; Male; Middle Aged; Splenomegaly/microbiology; Streptococcal Infections/complications/diagnosis/dr

SCID; Relapsing Fever/*microbiology/pathology; Spirochaetales/metabolism; Spleen/metabolism; Splenon
ɔmbination; Female; Hepatomegaly/microbiology; Humans; Male; Mexico; Milk/microbiology; Recurrence; Re

s
Diseases/complications/*microbiology; Splenomegaly/etiology/pathology; Tomography, X-Ray Computec
idemiology/microbiology; Dairy Products/adverse effects/microbiology; Drug Resistance, Multiple, Bacteri
ry/genetics; Ecuador; Electrophoresis, Polyacrylamide Gel; Fluorescent Antibody Technique; Humans; Mo
ive Agents/administration & dosage; Infusions, Intravenous; Leukocytosis/*etiology; Liver/pathology; Live

Infections, Animal/genetics/*immunology/*prevention & control; Salmonella typhimurium/*immunology;
n & purification; Salmonella Infections/complications/*epidemiology/microbiology/mortality
nent; Saudi Arabia/epidemiology; Severity of Illness Index; Sex Distribution
y/etiology/pathology/physiopathology; Stomach/blood supply; Veins
euraminic Acid/blood; Phagocytosis/physiology; Rats; Rats, Sprague-Dawley; Spleen/physiology; Streptoc

ɪock, Septic/*etiology/microbiology/pathology; Streptococcus pneumoniae/genetics/*isolation & purific
al data; Humans; Incidence; Infant; Lung Diseases/microbiology; Male; Microbial Sensitivity Tests; Myanmar
of the chronic disease. We assessed the occurrence of bacterial peritonitis among patients of HVD with
stiocytosis, Non-Langerhans-Cell/diagnosis/*pathology; Humans; Leukemia/diagnosis; Male; Pancytopeni
Female; Humans; Kidney Diseases/etiology; Lung Diseases/etiology; Male; Middle Aged; Prognosis; Rheuma
ic use; Gastroenteritis/complications/microbiology; Gentamicins/therapeutic use; Humans; Immunocompı
almonella paratyphi B/*isolation & purification; Severity of Illness Index; Splenomegaly/microbiology
diagnosis/microbiology; Spleen/microbiology; Splenic Rupture/*diagnosis/diagnostic imaging; Splenomega
parasitology; Magnetic Resonance Imaging; Morbidity; Salmonella Infections/complications; Schistosomiasi
ıs/diagnosis/drug therapy/*epidemiology/microbiology; Gram-Positive Bacterial Infections/diagnosis/drug

ons/complications/*epidemiology/mortality; Health Status Indicators; Humans; Male; Middle Aged; Mycoba
ibitors; Nitric Oxide Synthase Type II; Nitrites/metabolism; Salmonella Infections, Animal/*immunology/mi
Retrospective Studies; *Tomography, X-Ray Computed
ırbital/*adverse effects/therapeutic use; Primidone/*adverse effects/therapeutic use; Seizures/drug th

ial Sensitivity Tests; Middle Aged; Minnesota; Postoperative Complications/diagnosis/mortality/therapy; Pr
; Propionibacterium acnes/*immunology; Recombinant Proteins/pharmacology; Splenomegaly/immunolog
; Neutrophils/*immunology; Recombinant Proteins/administration & dosage/therapeutic use; Remission I
cardium/pathology; Prospective Studies; Spleen/pathology; Splenomegaly/microbiology; Virulence
/physiopathology; Male; Mental Disorders/physiopathology; Middle Aged; Prospective Studies; Sensitivity ar
Penicillium/drug effects/*isolation & purification; Prospective Studies; Thailand
emically induced; Staphylococcal Infections; Urticaria/chemically induced
emaker, Artificial/*adverse effects; Pseudomonas aeruginosa/isolation & purification; Retrospective Studi

asteurella Infections/*microbiology/*veterinary

ns

Splenomegaly; *Ultracentrifugation; *Waldenstrom Macroglobulinemia

entia tsutsugamushi/*isolation & purification; Plasmodium vivax/*isolation & purification; Republic of Kore
ıdex; Splenomegaly/pathology

emic/complications/immunology/*therapy; Lupus Nephritis/etiology/prevention & control; Mice; Mice, Ir
y/microbiology; Turkey/epidemiology; Young Adult

eses and Implants; Prosthesis-Related Infections/*immunology; Pseudomonas Infections; Pseudomonas

cioeconomic Factors; Splenomegaly/pathology; Streptococcal Infections/*epidemiology/pathology
complications/diagnosis/drug therapy; *Membranes, Artificial; Middle Aged; Molecular Weight; Pancytopenia

iagnosis/parasitology; Pharyngitis/complications/microbiology; Splenomegaly/etiology; Streptococcal Infe

g/*pathology; Ultrasonography; Uremia/pathology/therapy
ıdies; Splenomegaly; Turkey/epidemiology
saccharides/blood/immunology; Liver Diseases/blood; Lymphocyte Activation; Male; Middle Aged; Splenom
ug ; *Streptococcus anginosus/isolation & purification/pathogenicity; Streptococcus milleri Group/isolat

negaly
trospective Studies; Risk Factors; Splenomegaly/microbiology; Travel

l
al; Female; Food Microbiology; Humans; Male; Middle Aged; Occupational Diseases/epidemiology/microbiolo
lecular Sequence Data; Peru; Polymerase Chain Reaction; Sequence Analysis, DNA; Serum/immunology/
er Failure, Acute/*etiology; Liver Function Tests; Methylprednisolone/administration & dosage; Prednisolc

Spleen/abnormalities/immunology/microbiology; Splenomegaly/genetics/immunology/microbiology

occal Infections/*genetics/metabolism; Streptococcus mutans/*genetics; Virulence

ation; Thrombocytopenia/therapy
/epidemiology; Needs Assessment; Patient Admission/*statistics & numerical data; Residence Characteri
ı ascites. One hundred and sixty seven consecutive patients with ascites, which included 91 patients witl
a/*etiology; Salmonella typhi/*isolation & purification; Splenomegaly/etiology/pathology; Staphylococcal I
toid Factor/blood; Splenomegaly/etiology
romised Host; Jaundice/etiology; Male; Splenectomy; Splenomegaly/etiology

aly/*diagnosis; Tomography, X-Ray Computed
s mansoni/complications/diagnosis/*epidemiology; Splenic Diseases/diagnosis/parasitology; Staphylococ
g therapy/*epidemiology/microbiology; Hospitalization; Humans; Incidence; Infant; Infant, Newborn; Male; M

cterium Infections/complications/*epidemiology/mortality; Mycobacterium tuberculosis/isolation & purifi
crobiology; Salmonella typhimurium/growth & development/*immunology; Spleen/immunology; Splenomeg

ıerapy/veterinary; Thrombocytopenia/chemically induced/*veterinary

ognosis; Survival Rate; Treatment Outcome
ʒy; Time Factors; Tumor Necrosis Factor-alpha/antagonists & inhibitors/immunology/toxicity
nduction

ıd Specificity; Splenomegaly/physiopathology; Sweating/physiology

es; Risk Factors; Sepsis/epidemiology/etiology; Sex Factors; Staphylococcus aureus/isolation & purificat

a; Retrospective Studies; Risk Factors; Scrub Typhus/*epidemiology/microbiology; Splenic Infarction/*ep

ıbred NZB; Myocardium/pathology; Organ Size; *Probiotics; Signal Transduction; T-Lymphocytes/immuno

aeruginosa/metabolism

a/etiology/pathology; Permeability; Respiration, Artificial; Shock, Septic/etiology; Vasoconstrictor Agents/

ctions/complications; Streptococcus pyogenes

egaly/blood; T-Lymphocytes/immunology; Young Adult
ion & purification/pathogenicity; *Suction; Treatment Outcome

gy; Retrospective Studies; Ribotyping; Splenomegaly/etiology; Tunisia/epidemiology; Young Adult
/microbiology; Spleen/microbiology/pathology; Travel; United States
one/administration & dosage; Splenomegaly/etiology; Still's Disease, Adult-Onset/*diagnosis/drug therapy

stics/statistics & numerical data; Sentinel Surveillance; Sex Distribution; Splenomegaly/microbiology
h HVD were examined for the presence of bacterial peritonitis. The ascitic fluids were examined for total
Infections/complications/microbiology; Typhoid Fever/complications/microbiology/*pathology; Urinary Tr

cal Infections/complications; Tomography; Ultrasonography
licrobial Sensitivity Tests; Prospective Studies; Risk Factors; Survival Rate

cation; Thailand/epidemiology
galy; Vaccination

ion; Staphylococcus epidermidis/isolation & purification

›idemiology

logy/metabolism; Vascular Diseases/etiology/*prevention & control

'therapeutic use

'; Ursodeoxycholic Acid/administration & dosage

and differential WBC count. The fluid and the blood were cultured for aerobic microorganisms by bedside
act Infections/complications/microbiology

inoculation in blood culture bottles. HVD is a common cause of non-cirrhotic high protein content ascit

es in Nepal. It was uniquely associated with high incidence of bacteremia (61%) and

ID	Language	Authors	Title	Journal	Year

Volume	Pages	Pub. Type	Abstract	Memo

ID	Language	Authors	Title

Journal	Year	Volume	Pages	Pub. Type	Abstract	Memo

ID	Langu	Authors	Title	Journal	Year	Volume

Pages	Pub. Type	Abstract	Memo

CQ番号	CQ名	検索式	文献数	検索DB
	splenomegaly and bacteremia	("Splenomegaly"[Text Word] OR "Enlarged Spleen"[Text Word]) AND ("Bacteremia"[MeSH Terms] OR "bacteremi*"[Text Word] OR "bacteraemi*"[Text Word])	71	PubMed
			0	
			0	
			0	
	14	#1 AND #2	71	案2
	13	#11 AND #12	0	
	12	"Splenic Infarction"[Mesh] OR "splenic infarct*"	1697	
	11	#1 AND #6 AND #9	22	
	10	#1 AND #2 AND #6 AND #9	0	案1
	9	#7 OR #8	3379240	
	8	infection*[TIAB]	1545072	
	7	"Infections"[Mesh]	2794654	
	6	#3 OR #4 OR #5	129024	
	5	"aortic graft"[TIAB] OR "Aortic endograft"[TIAE	1680	
	4	"Aorta/surgery"[Mesh]	25864	
	3	"Vascular Grafting"[Mesh]	108762	
	2	"Bacteremia"[Mesh] OR bacteremi*[TW] OR bac	50644	
	1	"Splenomegaly"[TW] OR "Enlarged Spleen"[TW]	21507	

検索担当者	検索実行日	保存ファイル名	メモ
河合	2021/9/1		

PubMed 医中誌 Cochrane | JMEDPlus
